# Development of Novel Membrane Disrupting Lipoguanidine Compounds Sensitizing Gram-Negative Bacteria to Antibiotics

**DOI:** 10.1021/acsmedchemlett.3c00460

**Published:** 2024-01-09

**Authors:** Seong-Heun Kim, Charlotte K. Hind, Guilherme F. S. Fernandes, Jingyue Wu, Dorothy Semenya, Melanie Clifford, Caleb Marsh, Silvia Anselmi, A. James Mason, Kenneth D. Bruce, J. Mark Sutton, Daniele Castagnolo

**Affiliations:** † Department of Chemistry, University College London, 20 Gordon Street, London WC1H 0AJ, United Kingdom; ‡ Institute of Pharmaceutical Science, School of Cancer & Pharmaceutical Science, King’s College London, 150 Stamford Street, London SE1 9NH, United Kingdom; § Antimicrobial Discovery, Development and Diagnostics, Vaccine Development and Evaluation Centre, UKHSA Porton Down, Salisbury SP4 0JG, United Kingdom

**Keywords:** Lipoguanidine, Guanidine, Antibacterials, Antibiotic, Synergistic Activity

## Abstract

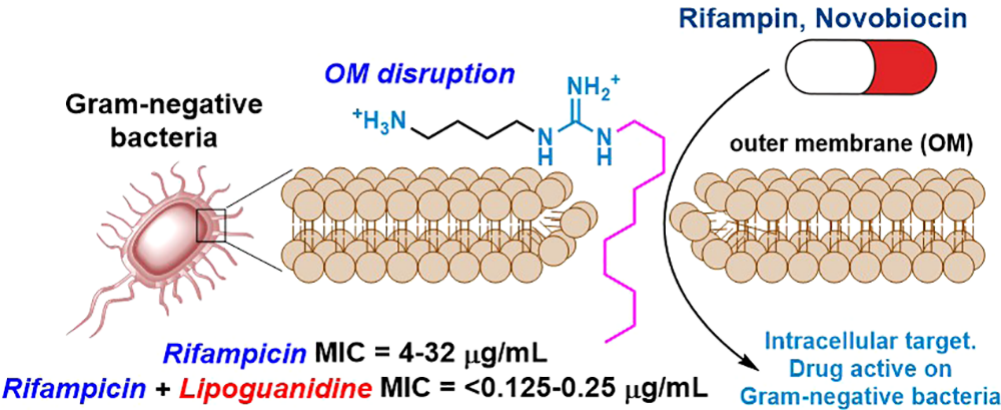

A new class of amphiphilic molecules, the lipoguanidines, designed as hybrids of guanidine and fatty acid compounds, has been synthesized and developed. The new molecules present both a guanidine polar head and a lipophilic tail that allow them to disrupt bacterial membranes and to sensitize Gram-negative bacteria to the action of the narrow-spectrum antibiotics rifampicin and novobiocin. The lipoguanidine **5g** sensitizes *Klebsiella pneumonia*, *Acinetobacter baumannii*, *Pseudomonas aeruginosa*, and *Escherichia coli* to rifampicin, thereby reducing the antibiotic minimum inhibitory concentrations (MIC) up to 256-fold. Similarly, **5g** is able to potentiate novobiocin up to 64-fold, thereby showing a broad spectrum of antibiotic potentiating activity. Toxicity and mechanism studies revealed the potential of **5g** to work synergistically with rifampicin through the disruption of bacterial membranes without affecting eukaryotic cells.

Antibiotics have long been a powerful tool in modern medicine, but their effectiveness is increasingly being undermined by the emergence of antibiotic-resistant bacteria.^1^ The overuse and misuse of antibiotics have led to the alarming situation where pathogens are becoming resistant to many commonly used antibiotic regimes.^2^ The scarcity of new antibiotic development is a significant contributing factor to the rise of antibiotic resistance,^3,4^ and thus, the development of new treatments for bacterial infections is considered critical by the World Health Organization (WHO). While some new antibiotic classes targeting Gram-positive bacteria have been introduced to the market within the last 20 years,^5−8^ only a few antibiotics that target Gram-negative bacteria are currently in the clinical pipeline.^9−13^ Such failure is largely attributed to the inability of many drugs to cross both the Gram-negative outer membrane (OM) and the inner membrane (IM) and to accumulate within these bacteria. Indeed, even though the molecular targets of many Gram-positive active antibiotics are also present in Gram-negative bacteria, the Gram-negative OM acts as a further barrier preventing the entry of such drugs to the cell.^14,15^ OM disrupting or perturbing agents, such as peptides,^16^ the chelating agent ethylenediaminetetraacetic acid (EDTA),^17^ the antiprotozoal drug pentamidine,^18^ the antifungal agent amphotericin B,^19^ or the cationic antibiotics,^20−22^ have shown their potential in sensitizing Gram-negative bacteria to the action of Gram-positive active antibiotics. The development of drugs able to disrupt bacterial membranes and to sensitize Gram-negative bacteria to antibiotics offers the possibility of turning narrow-spectrum antibiotics, like rifampicin or novobiocin, into broad-spectrum antibacterials.

Herein, we report the design and discovery of a new class of membrane-disrupting molecules, which we named lipoguanidines, presenting a general amphiphilic structure, **A** (Figure 1). The new molecules have been designed as hybrids of guanidines and fatty acids and they present both a guanidine polar head and a lipophilic tail.

**Figure 1:**
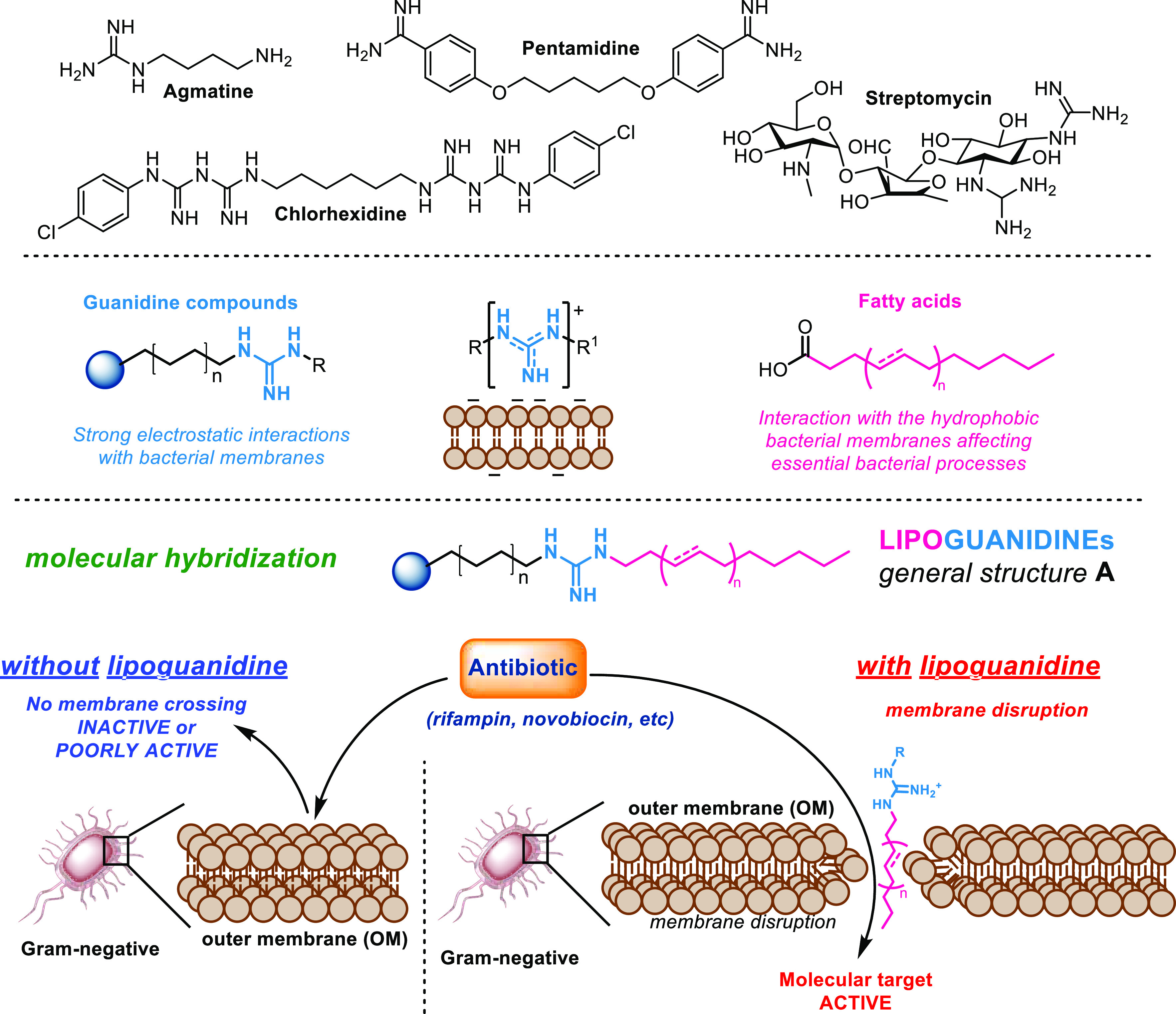
Guanidine antimicrobials and lipoguanidine design.

Many antimicrobial agents, including the cholic acid derivatives,^20^ contain a guanidine functional group.^23−29^ Guanidines can be protonated and positively charged at physiological pH in a similar way to the antibacterial quaternary ammonium compounds (QAC).^30,31^ Thus, a positively charged guanidine compound can facilitate the disruption of bacterial cell membranes through electrostatic interactions with the membrane anionic lipids.^32^ Since mammalian cells possess mainly neutral and uncharged lipids, guanidine compounds can selectively disrupt bacteria over mammalian cell membranes. Similarly, fatty acids exhibit antibacterial activity^33−35^ by interacting with hydrophobic bacterial membranes and impacting essential bacterial processes. The merging of hydrophilic guanidines with lipophilic fatty acids led to the design of novel lipoguanidine compounds that are able to sensitize Gram-negative bacteria to narrow-spectrum antibiotics.

A series of lipoguanidine derivatives **5a**–**j** was designed and synthesized according to Table 1. Agmatine, a natural guanidine compound bearing two protonable moieties, an amine, and a guanidine, was chosen as the template for the synthesis of lipoguanidine derivatives.^26^ Previous literature^36−39^ showed that agmatine and guanidines derivatives possess good antibacterial properties and that the guanidine group is essential for their activity. The 1,3-bis(*tert*-butoxycarbonyl)-2-methyl-2-thiopseudourea **1** was first reacted with different saturated and unsaturated fatty alcohols affording the alkyl-thiourea derivatives **2a**–**g**. Thioureas **2a**–**g** were then reacted with the diamines **3** and **4** to give, after acid-mediated Boc deprotection, the desired lipoguanidines **5a**–**j**. The derivative **6** bearing a second alkylated guanidine moiety was prepared by reacting **4** with 2 equiv of **2a**. A series of diguanidine compounds **8a**–**f** equipped with a single lipophilic chain was then synthesized from the Boc-amino-guanidines **7a** and **7b** through guanylation with **2a**–**g** followed by Boc deprotection. The derivatives **5k** and **8f** bearing no lipophilic chains were also prepared to evaluate the importance of the fatty chains for the lipoguanidine membrane disrupting activity. Finally, a branched triamine-lipoguanidine compound **10** was synthesized from the tetra-amine **9** with the aim to evaluate the role that multiple protonable amino groups might play on the lipoguanidines activity.

**Table 1:**
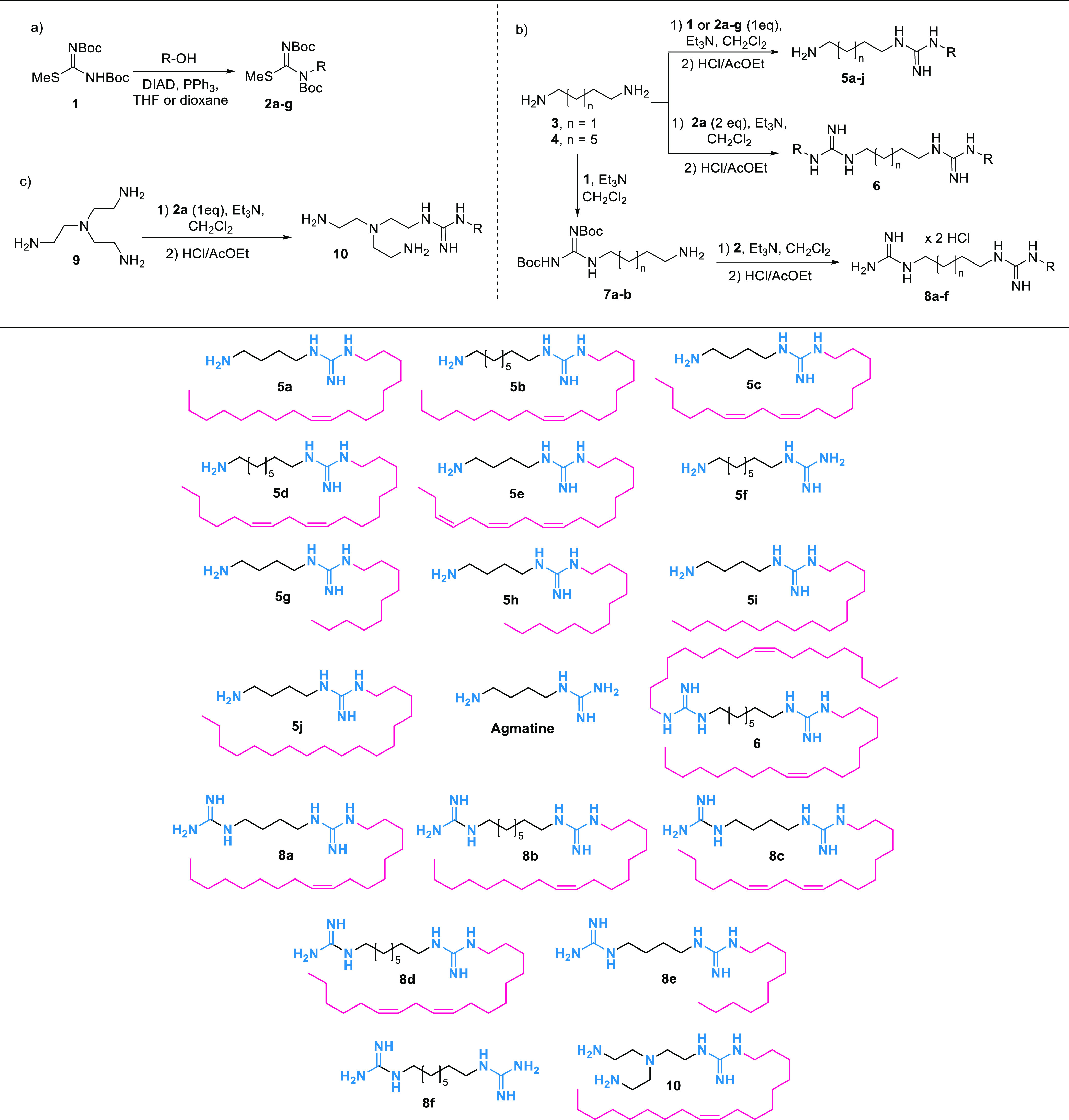
First Series of Lipoguanidine Compounds

The antibacterial activity of compounds **5a**–**j**, **6**, **8a**–**f**, and **10** was first evaluated against a panel of drug-susceptible and drug-resistant Gram-positive and Gram-negative bacterial strains (Table 2). All lipoguanidines showed good activity against Gram-positive bacteria. A significant structure–activity correlation was observed for lipoguanidines **5a**–**j**, which exhibited good activity against wild-type and drug-resistant *Staphylococcus aureus* and *Enterococci* strains (*Enterococci faecalis* and *Enterococci faecium*) with minimum inhibitory concentrations (MICs) ranging between 2 and 8 μg/mL. Compounds **5g** (containing a shorter side chain) and **5f** (no lipophilic chain) were found to be inactive against Gram-positive bacteria, which suggests a correlation between the lipophilicity of the chain and the antibacterial activity. Compound **6**, which contains two guanidine groups and two lipophilic chains, was found to be poorly active, while derivatives **8a**–**d** and **10** showed a biological profile similar to lipoguanidines **5a**–**j**. Such data indicate that the replacement of the amine group in **5a**–**j** with a second guanidine moiety does not affect their antibacterial profile, whereas the introduction of a second lipophilic chain in **6** is not tolerated. Compound **8f** containing no lipophilic chain proved to be inactive against all strains of bacteria, thereby confirming the key role of the fatty chain. As a general trend, the compounds bearing a guanidine moiety, a saturated or unsaturated lipophilic chain, and a second protonable group (guanidine or amine) showed the best activities against Gram-positive bacteria. However, most lipoguanidines proved to be moderately or poorly active against Gram-negative bacteria. Compounds **5a** and **5b** containing an oleyl chain showed activity against *Acinetobacter baumannii*, *Klebsiella pneumoniae*, and *Escherichia coli* strains with MIC = 8–16 μg/mL. A similar antibacterial profile was also observed for compounds **5c** and **5d**, which incorporate a linoleyl chain.

**Table 2:** Evaluation of the Antibacterial Activity of Lipoguanidines

compd	MIC (μg/mL)													
	Gram-positive							Gram-negative						
	*S. aureus*				*E. faecalis*		*E. faecium*	*K. pneumoniae*		*A. baumannii*		*P. aeruginosa*		*E. coli*
	MSSA ATCC 9144	MRSA NCTC 13616	MRSA USA 300	MRSA SA 1199B	VSE NCTC 775	VRE NCTC 12201	VRE NCTC 12204	NCTC 13368	M6	AYE	ATCC 17978	PAO1	NCTC 13437	NCTC 12923
**5a**	4	4	2	4	4	4	4	128	8	16	8	128	>128	8
**5b**	4	4	2	4	4–8	2	4	32–128	>128	32	8–16	>128	>128	16
**5c**	8	16	8	16	32	16	16	16	8	8	8	16	>64	4
**5d**	2–4	4	2–4	4	8	4	4	32	16	16	8	>128	>128	8
**5e**	8	8	4	8	8	8	8	>128	128	128	128	>128	>128	32
**5f**	128	>128	128	>128	>128	>128	>128	>128	>128	>128	>128	>128	>128	128
**5g**	16	32	32	32	128	>128	>128	>128	>128	>128	>128	>128	>128	128
**5h**	2	4	4	4	8	16	16	>128	>128	>128	128	>128	64	16
**5i**	2	2–4	2	4	2	2	2	>128	8–64	32→128	4–8	>128	>128	32–64
**5j**	8	4	2	4	2	2	2	>128	>128	128	16–32	>128	>128	>128
**6**	64	>64	64	32–64	64	>64	32	>128	>128	>128	128	>128	>128	128
**8a**	4	2	2	2	2	2	2	128	16	32	8	128	>128	8
**8b**	4–8	4–8	2–4	4–8	4–8	4	4	128	64	16–64	16	>128	>128	32
**8c**	4	4	4	4	8	8	8	>64	32	>64	32	64	64	8
**8d**	2–4	4	2	2–4	4	4	2	32–64	16–32	16–64	4–8	>128	>128	8
**8e**	ND^a^	ND^a^	ND^a^	ND^a^	ND^a^	ND^a^	ND^a^	>128	>128	>128	>128	64	>128	64
**8f**	128	>128	128	>128	>128	>128	>128	>128	>128	>128	>128	>128	>128	>128
**10**	8	4	4	4	4	4	4	>128	>128	128	64	64	32–64	8–16
agmatine	ND^a^	ND^a^	ND^a^	ND^a^	ND^a^	ND^a^	ND^a^	>128	>128	>128	>128	>128	>128	>128

a Not determined.

In contrast, compounds **5g** and **5h** bearing a saturated lipophilic chain were inactive against the Gram-negative strains, though the exception of **5i** and **5j** showed moderate activity against *A. baumannii* ATCC 17978 (MIC = 4–8 and 16–32 μg/mL, respectively). The lipoguanidines **8a**–**e** bearing two guanidine groups showed a biological profile similar to that of their corresponding amino-lipoguanidines **5a**–**j**. As observed in Gram-positive bacteria, both the aminoguanidines **5f** and **8f** bearing no lipophilic side chain, as well as agmatine, were completely inactive against all the Gram-negative strains, which confirmed the key role of the alkyl chain in improving the antibacterial profile of the lipoguanidines.

The polyamino-lipoguanidine **10** also showed no activity against most of the Gram-negative strains apart from *E. coli* (MIC = 8–16 μg/mL). The derivatives **5a**, **5d**, **5g**, **5h**, **5j**, **8a**, and **8d** were also screened against Gram-negative bacteria in combination with the membrane permeabilizing agent polymyxin B nonapeptide (PMBN) to determine if the lower activity observed against Gram-negative bacteria was ascribable to their inability to enter the cells by crossing their OM (Table S1). Gram-negative bacteria were treated with a range of concentrations of lipoguanidine compounds in the presence of PMBN at a fixed concentration of 30 μg/mL, which was able to permeabilize the OM without causing more than 20% growth inhibition for each strain tested. The observed MICs with and without PMBN were identical, and no improvement in activity was observed, which suggested that the lipoguanidines had already achieved the maximum OM penetration and bacterial inhibition.

## Sensitization of Gram-Negative Bacteria to Rifampicin by Lipoguanidines

The ability of lipoguanidine compounds to sensitize Gram-negative bacteria to the action of the narrow-spectrum antibiotic rifampicin through membrane disruption was then evaluated (Table 3). Compounds **5a**–**j** and **8a**–**f** were tested against a panel of Gram-negative bacteria in combination with rifampicin. Rifampicin is poorly active against Gram-negative bacteria (entry 1, Table 3) because of its poor ability to cross the OM and, as a consequence, to reach its molecular target, as confirmed by the addition of the membrane permeabilizer PMBN (entry 17, Table 3).

**Table 3:**
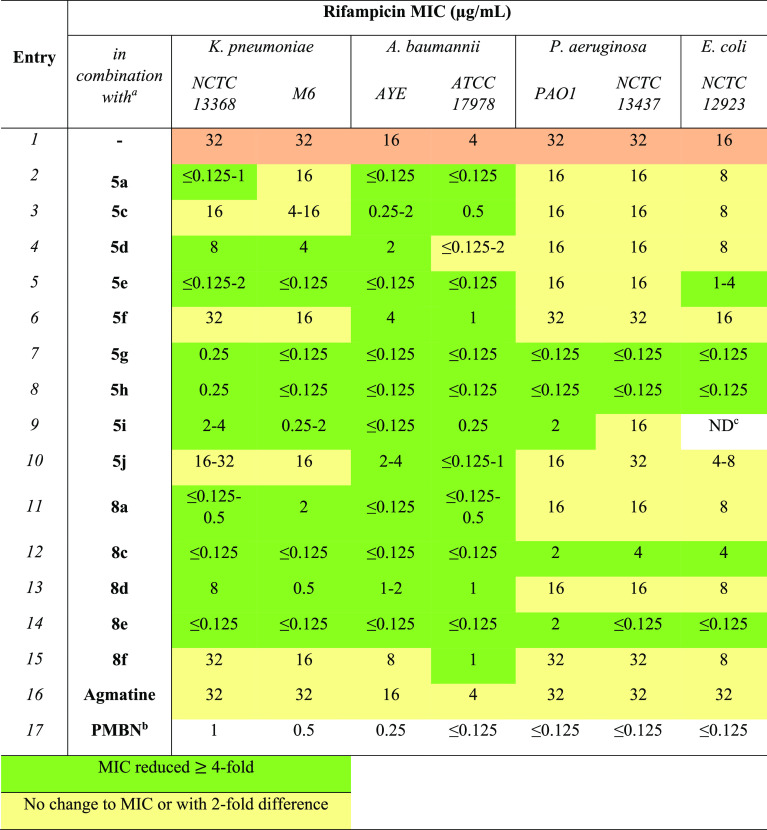
Synergistic Activity of Rifampicin in Combination with Lipoguanidines

a Lipoguanidine was added at a concentration of ≤0.25 × MIC. The exact concentration of lipoguanidines **5** and **8** used with each different bacterial strain for the synergistic study with rifampicin is reported in the Supporting Information Table S2.

b PMBN (30 μg/mL) was added.

c Not determined.

Gram-negative bacteria were treated with a range of concentrations of rifampicin in the presence of the lipoguanidine compounds **5a**–**j** and **8a**–**f** at a concentration of ≤0.25 × MIC. A ≥4-fold decrease in rifampicin MIC in the presence of a lipoguanidine derivative indicates that the bacteria are sensitized to the antibiotic. Lipoguanidines **5a**, **5d**–**j**, and **8a**–**e** exhibited strong rifampicin potentiating activity against some or all *K. pneumoniae* and *A. baumannii* strains with a >4-fold reduction in rifampicin MIC. The oleyl lipoguanidine **5a** demonstrated excellent rifampicin potentiating activity with rifampicin MIC reduced to ≤0.125 μg/mL against both drug-sensitive (ATCC 17978) and drug-resistant (AYE) *A. baumannii* strains and *K. pneumoniae* NCTC 13368 (entry 2, Table 3). A similar profile was observed for the linoleyl derivatives **5c** and **5d** (entries 3–4, Table 3), while the linolenyl **5e** reduced the rifampin MIC to ≤0.125 μg/mL in both *K. pneumoniae* strains (entry 5, Table 3). None of the lipoguanidines **5a**–**e** bearing an unsaturated lipophilic chain were able to improve the rifampicin activity against *Pseudomonas aeruginosa*, and only **5e** showed a sensitizing action on *E. coli*. Remarkably, lipoguanidines **5g** and **5h** bearing, respectively, a decyl- and a dodecyl-saturated lipophilic chain exhibited an excellent sensitizing effect on all Gram-negative strains, including the *P. aeruginosa* strains, with rifampicin MIC ≤ 0.125 μg/mL (entries 6 and 7, Table 3). Interestingly, even though **5g** and **5h** did not have any activity against any Gram-negative bacteria, they showed a remarkable sensitizing effect on all Gram-negative strains, including *P. aeruginosa* and *E. coli*. Such data are remarkable, especially when compared with those of the lipoguanidines **5a**, **5d**, and **5e**, which showed good Gram-negative inhibitory activity but no sensitizing activity on *P. aeruginosa* and *E. coli* species, thereby suggesting a correlation between the antibacterial activity/sensitizing effect and the unsaturated/saturated lipophilic chains. Interestingly, an increase in the length of the saturated lipophilic chain in **5i** and **5j** led to a decrease of the sensitizing action of lipoguanidines, especially against *P. aeruginosa* (entries 8 and 9, Table 3).

Derivatives **8** bearing two guanidine groups also showed, in many cases, a strong sensitizing effect against Gram-negative bacteria. The lipoguanidine **8e** bearing a decyl chain like **5g** showed the best sensitizing activity with a remarkable improvement of rifampicin MIC also against *P. aeruginosa* (entry 14, Table 3). As observed for compounds **5**, the derivatives **8a**–**d** bearing an unsaturated lipophilic chain showed good-to-moderate activity against Gram-negative bacteria and sensitizing activity against *K. pneumoniae* and *A. baumannii* species but were unable to improve the rifampicin MIC against *P. aeruginosa* and *E. coli*. Conversely, compound **8e** bearing a fully saturated lipophilic chain like **5g** and **5h** showed no activity against Gram-negative bacteria but a strong sensitizing effect on *P. aeruginosa* and *E. coli*, thereby confirming the correlation between the sensitizing activity and the lipophilic chain degree of saturation. Notably, guanidines **5f** and **8f** did not show any rifampicin MIC reduction except for some mild improvement on *A. baumannii*, which clearly indicates the essential role of a lipophilic chain in the sensitization of Gram-negative bacteria (entries 10 and 15, Table 3).

## Assessment of the Hemolytic Activity and Toxicity of Lipoguanidines **5** and **8**

To evaluate the potential of lipoguanidines as therapeutic agents, the hemolytic activity of the most promising compounds was evaluated. All tested compounds demonstrated a noticeable hemolytic effect, with **5c** being the most hemolytic (100.0%) and **5g** the least (8.2%). Compounds **5a**, **5c**, **5e**, and **8a** bearing unsaturated lipophilic chains were found toxic to erythrocytes, which suggests a correlation between the degree of unsaturation in the lipophilic chain and hemolysis. Saturated derivatives **5h**, **5i**, and **8e**, also exhibited high hemolytic activity, while **5g** showed a better cytotoxic profile and a large difference between its antibiotic potentiation activity and hemolytic effect because it potentiates the activity of rifampicin against all strains of Gram-negative bacteria when used at 32 μg/mL and only causes 8.2% hemolysis at 512 μg/mL. Therefore, **5g** emerged as the most promising lipoguanidine hit.

An *in vivo* toxicity assay using *Galleria mellonella* was then carried out to evaluate the toxicity of **5g** in a nonanimal infection model.^40,41^ Ten wax moth larvae were injected with **5g** at concentrations of 50 mg/kg, 20 mg/kg, and 10 mg/kg (Table S5, Supporting Information). By the end point of the assay at 120 h, 10 of 10 larvae were still alive at all doses. Such data are in line with the *in vitro* hemolysis assay data, which indicates that **5g** is not toxic on eukaryotic cells and in *Galleria* models.

## Assessment of the Antibiotic Synergism between Rifampicin and **5g** by Checkerboard Assay

**Figure 2:**
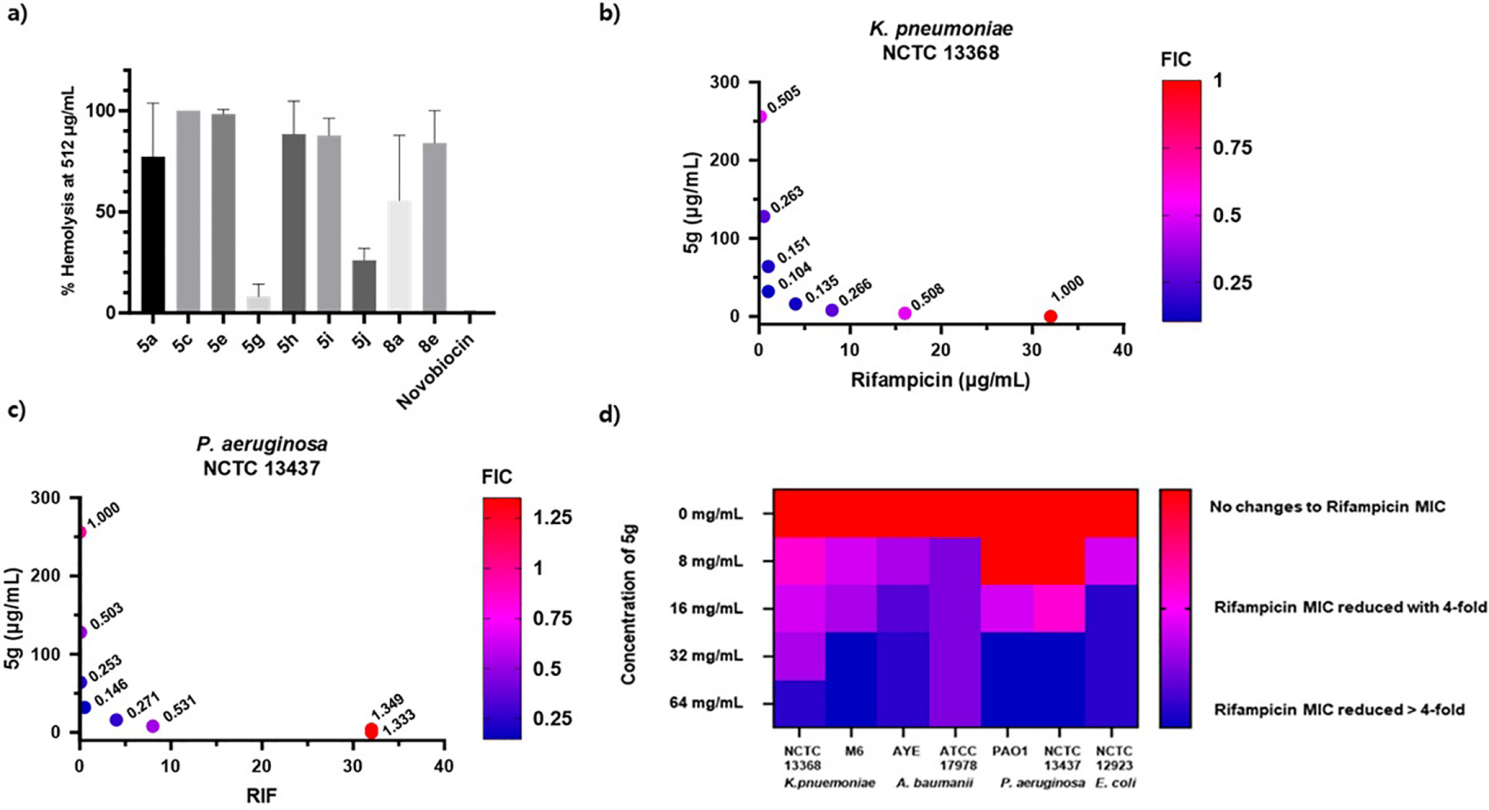
(a) % Hemolysis of lipoguanidines at 512 μg/mL; (b) synergism of **5g** with rifampicin on *K. pneumoniae* NCTC 13368 and FIC value; (c) synergism of **5g** with rifampicin on *P. aeruginosa* NCTC 13437 and FIC value; and (d) evaluation of the Gram-negative bacteria sensitizing activity of **5g** at different concentrations.

In the initial synergy screening, the sensitizing activity of lipoguanidines was assessed using a single concentration of lipoguanidine (generally corresponding to ≤0.25 × MIC). In the case of **5g**, which showed no activity against most Gram-negative strains (>128 μg/mL) and moderate activity against *A. baumannii* ATCC 17978 (16–32 μg/mL), concentrations around 32 μg/mL were used across the panel. The different concentrations of **5g** at which a sensitizing effect of Gram-negative bacteria to rifampicin can be observed were then evaluated (Figure 2d). When **5g** was used at 32 μg/mL, a significant reduction in rifampicin MIC in all Gram-negative strains was observed. Although higher concentrations of **5g** (64 μg/mL) showed an improved rifampicin potentiation activity in *K. pneumoniae* NCTC 13368, the concentration of 32 μg/mL was more efficient in producing significant changes in rifampicin MIC. At the lower concentration of **5g** (8–16 μg/mL) a reduction in the rifampicin potentiation activity was still observed in *K. pneumonia* and *P. aeruginosa* strains. The optimal concentration of **5g** for synergism activity was set at 32 μg/mL. Since **5g** showed antimicrobial activity against *A. baumannii* ATCC 17978 at 32 μg/mL, it is not possible to unambiguously conclude that, for this strain, the concentration was potentiating.

A checkerboard assay was then carried out to assess the efficacy of the combination of rifampicin and **5g**. The synergistic activity of the two agents was measured using the fractional inhibitory concentration (FIC) index.^42^ A FIC value of ≤0.5 indicates a strong synergistic effect, while values of 0.5 to <1 indicate weak synergism. The synergistic activity of the two compounds was tested against the drug-resistant strains *K. pneumoniae* NCTC 13368 and *P. aeruginosa* NCTC 13437. These strains were selected because previous research in antimicrobial evaluation studies has repeatedly suggested that they have less permeable membranes and, thus, were ideal worst-case models to evaluate the synergistic effect of membrane-disrupting agent **5g**. A strong synergy between **5g** and rifampicin was observed against both *K. pneumoniae* strain NCTC 13368 and *P. aeruginosa* strain NCTC 13437. The MIC values of both compounds were reduced when they were used in combination. FIC values of 0.10 for NCTC 13368 and 0.15 for NCTC 13437 indicate a strong synergistic effect between the two compounds (Figure 2b,c).

## Mechanism of Action of **5g**

In order to confirm the mode of action of **5g**, a 1-*N*-phenylnaphthylamine (NPN) uptake assay was carried out. NPN is a fluorescent probe that is unable to cross the Gram-negative bacteria OM because of its hydrophobic nature. When the outer membrane is damaged, the NPN gains access to the bacterial periplasmatic space, which results in a prominent fluorescent signal.

**Figure 3:**
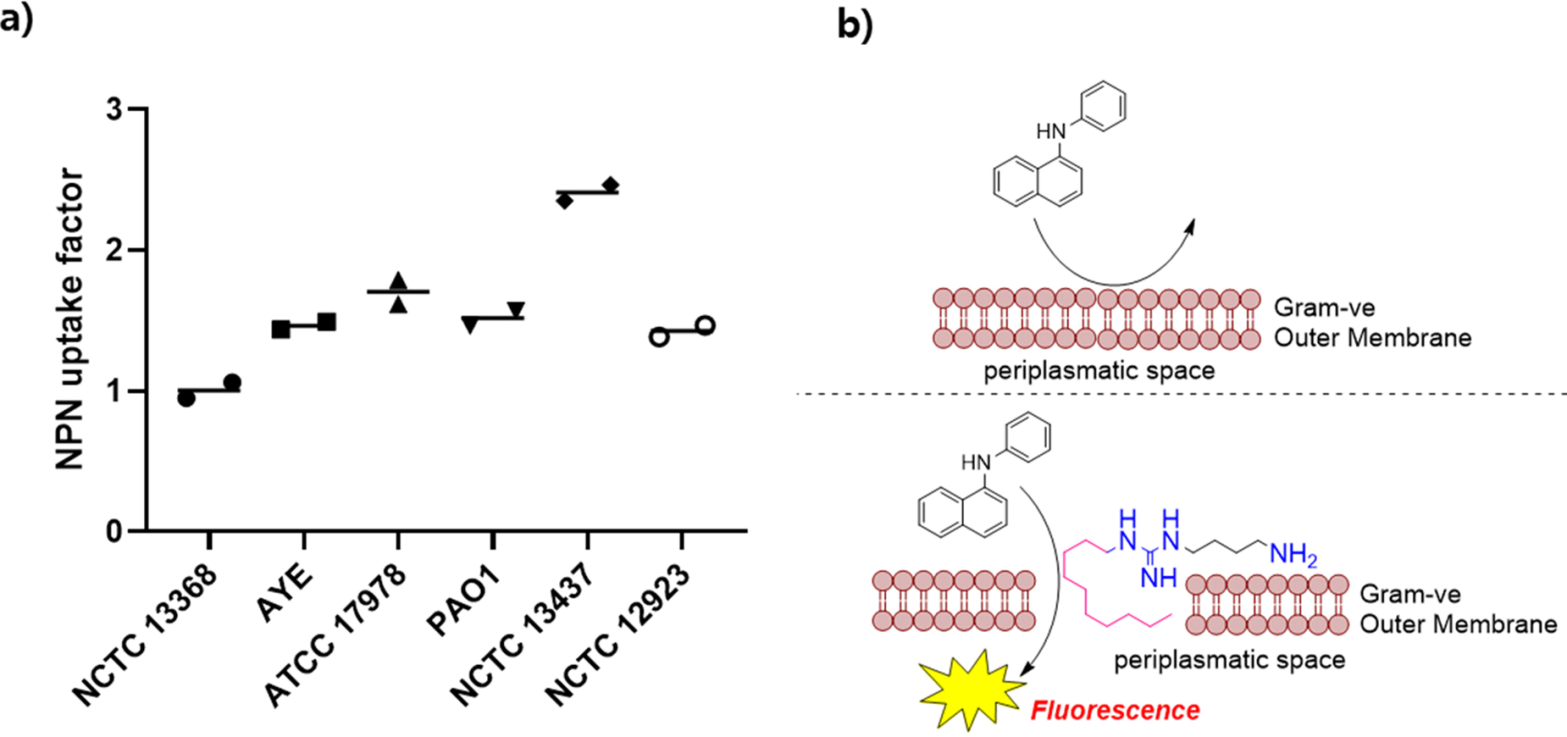
Evaluation of the mechanism of action of **5g** through the NPN assay. (A) OM permeabilization of Gram-negative bacteria by **5g**, as measured through NPN uptake. (B) Cartoon describing the suspected mechanism of action of **5g** on the OM of Gram-negative bacteria. For the time point, the NPN uptake was calculated using the following equations where *F*_obs_ is the observed fluorescence of the sample, *F*_0_ is the initial fluorescence of NPN with bacteria in the absence of any compound, and *F*_100_ is the fluorescence of NPN with bacteria upon the addition of 10 μg/mL of PMB. NPN uptake factor = (*F*_obs_*– F*_0_)/(*F*_100_*– F*_0_).

The NPN uptake factor for **5g** at 128 μg/mL was calculated in relation to the quantity of NPN taken up by the cells when treated with 10 μg/mL of the outer membrane permeabilizer polymyxin B (PMB).^43^ An NPN uptake factor of 1 indicates that the bacteria took up the same amount of NPN as when they were treated with PMB, thereby suggesting that NPN gained access to the bacteria because of the membrane-permeabilizing activity of **5g**. As shown in Figure 3, an NPN factor > 1 was observed against all Gram-negative strains, including *K. pneumoniae* NCTC 13368 and *P. aeruginosa* NCTC 13437, which are understood to have a more robust outer membrane. These data confirmed a membrane-disrupting activity for lipoguanidine **5g**.

## Synthesis, Biological Evaluation, and Structure–Activity Relationships (SARs) of Lipoguanidine Analogues

With the aim to further elucidate the SAR of the new lipoguanidine compounds and to identify key features requisite for synergistic activity, a series of derivatives was designed and synthesized (Table 4). The derivatives **11a** and **11b** bearing a tertiary amine group were synthesized through reaction of the decyl-thiourea **2d** with 4-dimethylaminobutylamine and pyrrolidine-butylamine. Derivative **11d**, which was designed as a zwitterionic lipoguanidine incorporating a carboxylate moiety, was obtained through the reaction of **2g** with 4-aminobutanoic acid. Lipoguanidines **11c**, **11e**, and **11f** lacking an amine group were synthesized by reacting **2g** with butyl-, decyl-, and oleylamine. Lastly, butylamine and diaminobutane were reacted with dodecyl-chloride and oleyl-chloride, respectively, to afford the amides **13a**–**c**, while the *N-*Boc-diamine **14** was reacted with octadecyl-isocyanate followed by Boc deprotection to obtain the urea **15**.

**Table 4:**
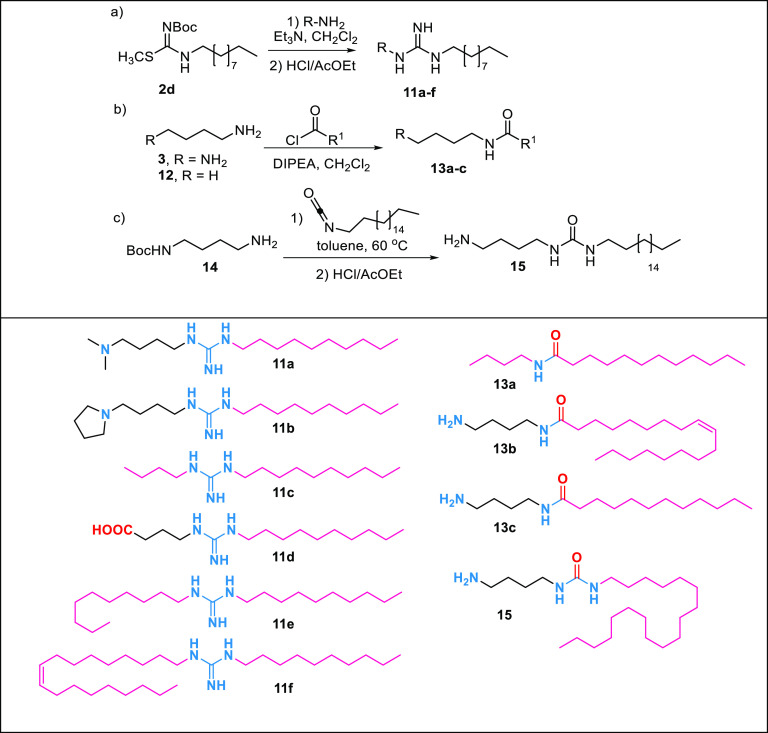
Synthesis of Lipoguanidine Analogues **11**, **13**, and **15**

All derivatives **11**, **13**, and **15** were first assessed for their activity against Gram-negative bacteria (Table S3). Only the dialkyl-lipoguanidines **11c** and **11e** showed moderate activity against strains of *A. baumannii*, *K. pneumoniae*, and *E. coli* with MICs of 16–32 μg/mL.

Derivatives **11**, **13**, and **15** were then tested at a concentration of ≤0.25 × MIC in combination with rifampicin to assess their ability to sensitize Gram-negative bacteria to the antibiotic (Table 5). Lipoguanidines **11a** and **11b** were able to sensitize all Gram-negative bacteria to rifampicin with a decrease of the rifampicin MIC > 4 fold. However, compared with the lipoguanidine **5g** bearing a primary amine as a second protonable group, **11a** and **11b** showed a lower sensitizing effect against *P. aeruginosa* strains PAO1 and NCTC 13437 (entries 2 and 3, Table 5). Guanidine **11c** showed excellent activity with rifampicin against *K. pneumoniae*, *A. baumannii*, and *E. coli* but no sensitizing effect on *P. aeruginosa* (entry 4, Table 5).

**Table 5:**
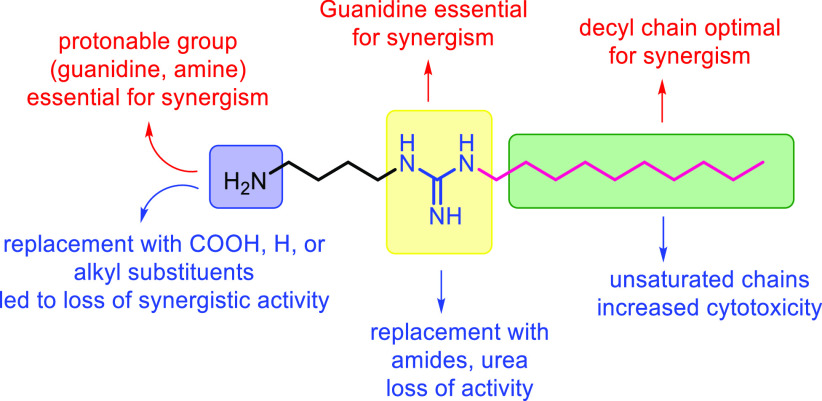

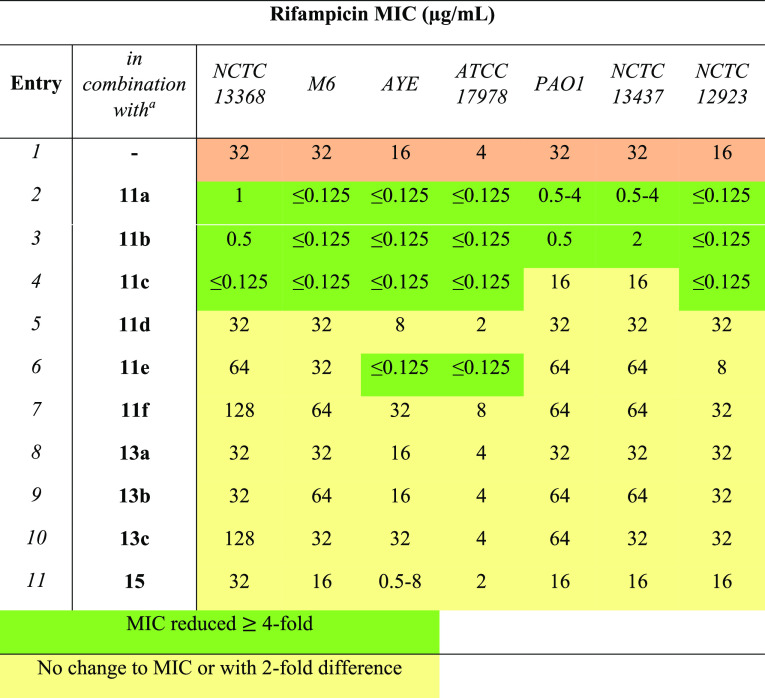
Synergistic Activity of Rifampicin in Combination with Lipoguanidines and SARs

a Lipoguanidine/guanidine/amide/urea was added at a concentration of ≤0.25 × MIC. The exact concentration of lipoguanidine/guanidine/amide/urea used with each different bacterial strain for the synergistic study with rifampicin is reported in the Supp. Information in Table S4.

Guanidine **11e** showed sensitizing activity only on *A. baumannii*, while no activity was observed on *K. pneumoniae* and *E. coli*. (entry 6, Table 5). Such data confirm that the presence of a second hydrophilic/protonable moiety in the lipoguanidine compounds is crucial in improving the sensitization of bacteria to rifampicin. Finally, the derivatives **11f** and **11d**, as well as the amides **13a**–**c** and urea **15**, showed no reduction of the rifampicin MIC, thereby confirming the crucial role of the guanidine group for the activity.

## Sensitization of Gram-Negative Bacteria to Novobiocin by Lipoguanidines

Lipoguanidines were finally assessed in combination with novobiocin against Gram-negative bacteria (Table 6). Compounds **5a**, **5e**, and **8a** effectively sensitized *A. baumannii* strains and *K. pneumoniae* M6 to novobiocin (entries 1, 3, and 6, Table 6). Remarkably, the less cytotoxic lipoguanidine **5g** and compound **8e** broadened novobiocin’s activity across various Gram-negative species (entries 5 and 7, Table 6). The lower potentiating effect of **5g** on *P. aeruginosa* with novobiocin than with rifampicin may be attributed to underlying mutations in these strains (i.e., DNA gyrase or efflux pump mutations) that may impact the novobiocin activity.

**Table 6:**
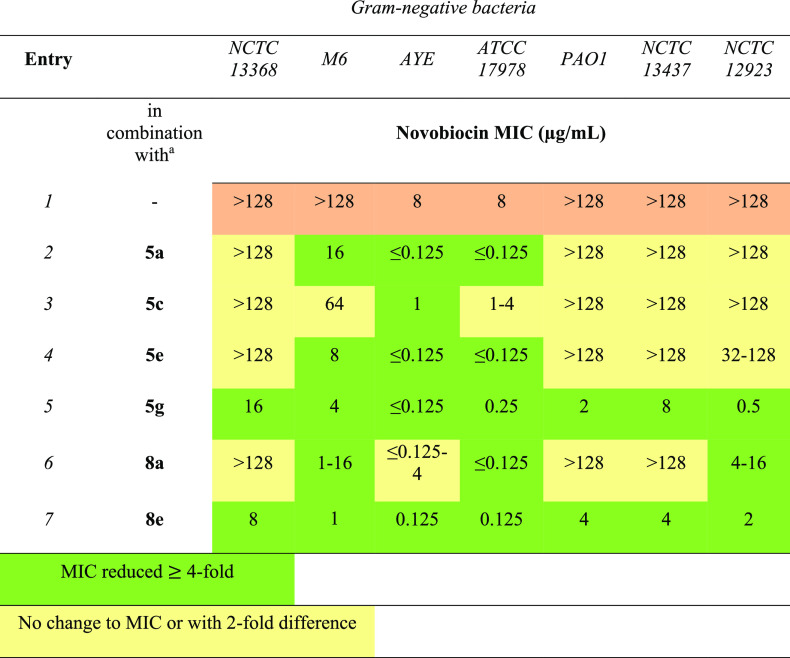
Sensitization of Gram-Negative Bacteria to Novobiocin by Lipoguanidines^33^

a Lipoguanidine was added at concentration of ≤0.25 × MIC. The exact concentration of lipoguanidines **5** and **8** used with each different bacterial strain for the synergistic study with rifampicin is reported in the Supporting Information Table S2.

The potentiating effect of lipoguanidine **5g** in combination with the antibiotics ampicillin, ceftazidime, doxycycline, and tobramycin was also investigated (Table S5). Only a potentiating effect of **5g** on doxycycline on *Klebsiella*, *Pseudomonas*, and *E. coli* species was observed.

In conclusion, we have designed and developed a novel class of structurally simple amphiphilic lipoguanidine compounds through the hybridization of aminoalkylguanidine derivatives and fatty acids. While lipoguanidines are not highly effective against Gram-negative bacteria, they exhibit synergy in combination with narrow-spectrum antibiotics, such as rifampicin and novobiocin. Lipoguanidine **5g** significantly lowered the MIC of rifampicin and novobiocin against both wild-type and drug-resistant Gram-negative bacteria (up to ≤0.125 μg/mL) through the permeabilization of the bacterial membranes, as confirmed through an NPN assay. Finally, **5g** demonstrated low toxicity to eukaryotic cells and living organisms, as indicated by hemolysis and *in vivo* toxicity assays.

## Supporting Information Available

Experimental procedures, characterization of chemical compounds, microbiological procedures and data, analytical data, HPLC traces, and NMR spectra (PDF)

## Abbreviations

OM: outer membrane
IM: inner membrane
MIC: minimum inhibitory concentration
QAC: quaternary ammonium compounds
PMBN: polymyxin B nonapeptide
FIC: fractional inhibitory concentration
NPN: 1-*N*-phenylnaphthylamine
SARs: structure–activity relationships
