# Supplementary material for: Development of Novel Membrane Disrupting Lipoguanidine Compounds Sensitizing Gram-Negative Bacteria to Antibiotics
Source: ACS Med Chem Lett. 2024 Jan 9;15(2):239–49. doi: 10.1021/acsmedchemlett.3c00460 (PMC10860194; doi:10.1021/acsmedchemlett.3c00460)
Supplement: Supplementary file 1 — ml3c00460_si_001.pdf [file ml3c00460_si_001.pdf]

Supplementary Information

Development of novel membrane disrupting lipoguanidine compounds sensitizing Gram-negative bacteria to antibiotics.

Seong-Heun Kim,[1,2] Charlotte K. Hind,[3] Guilherme F. S. Fernandes,[1] Jingyue Wu,[1] Dorothy Semenya,[2] Melanie Clifford,[3] Caleb Marsh,[3] Silvia Anselmi,[1] A. James Mason,[2] Kenneth D. Bruce,[2] J. Mark Sutton,[2,3,*] Daniele Castagnolo,[1,2,*]

[1] Department of Chemistry, University College London, 20 Gordon Street, London WC1H 0AJ, United Kingdom. [2] Institute of Pharmaceutical Science, School of Cancer & Pharmaceutical Science, King's College London, 150 Stamford Street, SE1 9NH, London, United Kingdom. [3] Antimicrobial Discovery, Development and Diagnostics, Vaccine Development and Evaluation Centre, UKHSA Porton Down, Salisbury SP4 0JG, United Kingdom.

*Corresponding author: d.castagnolo@ucl.ac.uk; mark.sutton@ukhsa.gov.uk.

1. Experiment section S2
2. Microbiological data S13
3. Biological data S18
4. ^{1}H and ^{13}C NMR spectra S20
5. HPLC Chromatograms S56

1. Experiment section

Chemistry. Commercially available reagents, chemicals, and solvents were used without further purification. The reactions were monitored through thin-layer chromatography (TLC) using commercially available precoated plates and visualized using UV light at 254 nm. Flash column chromatography was carried out using Sigma Aldrich silica gel (pore size 60 Å, particle size 40-63 μm). The ^{1}H NMR and ^{13}C NMR spectra were obtained with a Bruker® Ascend 400 MHz spectrometer at room temperature (rt) using deuterated chloroform ($CDCl_3$) or methanol (MeOD) as solvents. The chemical shifts were expressed in parts-per-million (ppm) relative to tetramethylsilane (TMS), and the coupling constants (*J*) were denoted in hertz (Hz). The spin multiplicities were reported as singlet (s), doublet (d), triplet (t), quartet (q), multiplet (m), doublet of doublet (dd), doublet of quartet (dq), doublet of doublet of triplet (ddt), pentet (p), heptept (h), multiplet (m). The HPLC purity analysis for the final compounds confirmed their purity to be greater than 95%. HRMS measurements were recorded on a Thermo Fisher LTQ Orbitrap XL Mass Spectrometer using low-resolution ESI or high-resolution nano ESI mass spectrometer. Compounds **1**, **3**, **4**, **9** and **12** were obtained commercially. No unexpected or unusually high safety hazards were encountered.

General procedure for the synthesis of S-methylisothioureas ***2a-g***. To a stirred solution of 1,3-bis(*tert*-butoxycarbonyl)-2-methyl-2-thiopseudourea (**1**) (1 mmol) in dry THF, Ph_3P (1.5 mmol) and the appropriate alcohol (1.3 mmol) were added. DIAD (1.5 mmol) was then added dropwise, and the reaction mixture was then stirred at reflux overnight. The crude mixture was concentrated in vacuum and then diluted with H_2O and CH_2Cl_2. The mixture was then extracted with CH_2Cl_2 (3 x 10 mL). The combined organic phases were washed with brine, dried over $MgSO_4$, and concentrated under

reduced pressure. The crude products were purified by flash column chromatography (SiO_2) using 1:9 Hexane/EtOAc, as the eluent to yield the *S*-methylisothiourea derivatives.

Compound (*2a*). Oil; yield 36%. ^{1}H NMR (400 MHz, $CDCl_3$) δ: 5.33 (q, J = 3.6, 1.9 Hz, 2H), 3.51 – 3.44 (m, 2H), 2.37 (s, 3H), 2.00 (d, J = 6.1 Hz, 4H), 1.65 (d, J = 5.3 Hz, 2H), 1.48 (d, J = 11.8 Hz, 18H), 1.27 (q, J = 9.2, 5.2 Hz, 22H), 0.87 (t, J = 6.6 Hz, 3H). ^{13}C NMR (101 MHz, $CDCl_3$) δ: 163.1, 157.9, 151.9, 129.9, 129.8, 82.1, 81.7, 49.1, 31.9, 29.8, 29.7, 29.7, 29.6, 29.5, 29.5, 29.4, 29.3, 29.2, 29.2, 28.9, 28.1, 28.0, 27.2, 26.8, 22.7, 21.6, 15.6, 14.1. LRMS m/z (ES+) m/z: 541.

Compound (2b). Oil; yield 25%. ^{1}H NMR (400 MHz, $CDCl_3$) δ: 5.33 (qd, *J* = 11.2, 9.2, 3.2 Hz, 4H), 3.52 – 3.41 (m, 2H), 2.75 (t, *J* = 6.5 Hz, 2H), 2.36 (s, 3H), 2.03 (q, *J* = 7.2 Hz, 4H), 1.68 – 1.58 (m, 2H), 1.47 (d, *J* = 11.5 Hz, 18H), 1.31 (d, *J* = 23.9 Hz, 16H), 0.87 (t, *J* = 6.7 Hz, 3H). ^{13}C NMR (101 MHz, $CDCl_3$) δ: 163.1, 158.0, 152.0, 130.3, 130.2, 128.1, 128.0, 82.1, 81.8, 49.1, 31.6, 29.7, 29.6, 29.4, 29.3, 29.3, 28.9, 28.2, 28.1, 27.3, 27.3, 26.9, 25.7, 22.7, 15.6, 14.1. LRMS m/z (ES+) m/z: 539.

Compound (2c). Oil; yield 63%. ^{1}H NMR (400 MHz, $CDCl_3$) δ: 5.36 – 5.18 (m, 6H), 3.45 – 3.37 (m, 2H), 2.73 (t, *J* = 6.1 Hz, 4H), 2.30 (s, 3H), 1.98 (p, *J* = 7.3 Hz, 4H), 1.64 – 1.53 (m, 2H), 1.42 (d, *J* = 11.4 Hz, 18H), 1.25 – 1.17 (m, 10H), 0.90 (t, *J* = 7.6 Hz, 3H). ^{13}C NMR (101 MHz, $CDCl_3$) δ: 163.0, 157.8, 151.8, 131.9, 130.3, 128.2, 127.7, 127.1, 82.0, 81.6, 53.4, 49.0, 31.5, 29.6, 29.6, 29.5, 29.2, 29.2, 28.8, 28.1, 28.0, 27.2, 27.2, 26.8, 25.6, 25.6, 25.5, 22.6, 20.5, 15.5, 14.3. LRMS m/z (ES+) m/z: 537.

Compound (2d). Oil; yield 51%. ^{1}H NMR (400 MHz, $CDCl_3$) δ: 3.47 – 3.40 (m, 2H), 2.33 (s, 3H), 1.64 – 1.56 (m, 2H), 1.44 (d, J = 11.4 Hz, 18H), 1.27 – 1.18 (m, 14H), 0.85 – 0.79 (m, 3H). ^{13}C NMR

(101 MHz, $CDCl_3$) δ: 163.0, 157.9, 151.8, 82.0, 81.7, 49.0, 31.9, 29.7, 29.6, 29.6, 29.6, 29.5, 29.3, 29.2, 28.8, 28.0, 28.0, 26.7, 22.6, 21.6, 15.5, 14.4, 14.1. HRMS (ESI): calcd for $C_{22}H_{42}O_4N_2{}^{32}S$ $(M + H)^+$ 420.28653, found 430.2851.

Compound (2e). Oil; yield 53%. ^1H NMR (400 MHz, $CDCl_3$) δ: 3.50 – 3.37 (m, 2H), 2.34 (s, 3H), 1.67 – 1.54 (m, 2H), 1.45 (d, J = 11.6 Hz, 18H), 1.23 (d, J = 10.0 Hz, 18H), 0.90 – 0.77 (m, 3H). ^{13}C NMR (101 MHz, $CDCl_3$) δ: 163.1, 157.9, 151.9, 82.1, 81.7, 49.1, 32.0, 29.7, 29.7, 29.7, 29.7, 29.6, 29.4, 29.3, 28.9, 28.1, 28.1, 26.8, 22.7, 15.6, 14.2. LRMS m/z (ES+) m/z: 459.

Compound (2f). Oil; yield 88%. ^1H NMR (400 MHz, $CDCl_3$) δ: 3.49 – 3.43 (m, 2H), 2.36 (s, 3H), 1.63 (dq, J = 11.3, 7.2 Hz, 2H), 1.47 (d, J = 11.7 Hz, 18H), 1.23 (d, J = 1.7 Hz, 26H), 0.88 – 0.83 (m, 3H). ^{13}C NMR (101 MHz, $CDCl_3$) δ: 163.1, 158.0, 152.0, 82.1, 81.8, 49.2, 32.0, 29.8, 29.8, 29.8, 29.7, 29.7, 29.5, 29.3, 28.9, 28.2, 28.1, 26.9, 22.8, 15.6, 14.2. LRMS m/z (ES+) m/z: 515.

Compound (2g). Oil; yield 81%. ^1H NMR (400 MHz, $CDCl_3$) δ: 3.51 – 3.44 (m, 2H), 2.37 (s, 3H), 1.67 – 1.61 (m, 2H), 1.50 (s, 9H), 1.47 (s, 9H), 1.24 (d, J = 1.8 Hz, 30H), 0.89 – 0.85 (m, 3H).^{13}C NMR (101 MHz, $CDCl_3$) δ: 13C NMR (101 MHz, CDCl3) δ 163.2, 160.1, 158.0, 152.0, 82.2, 81.8, 74.5, 49.2, 32.1, 29.8, 29.8, 29.8, 29.8, 29.7, 29.5, 29.3, 29.0, 28.2, 28.1, 26.9, 22.8, 21.7, 15.7, 14.5, 14.2. LRMS m/z (ES+) m/z: 543.

General procedure for the synthesis of the lipoguanidines. The appropriate amine **3**, **4**, **7a-b**, **9** (0.18 mmol) was dissolved in CH_2Cl_2 with Et_3N and a solution of the appropriate thiopseudourea **2a-g** (0.18 mmol) in CH_2Cl_2 was added dropwise. The mixture was stirred for 24-72 h. The solvent was evaporated under reduce pressure, and the residue was filtered on a pad of silica gel (SiO_2) using 8.5:1:0.5 EtOAc/MeOH/Et_3N to provide the desired di-Boc-lipoguanidine. The lipoguanidine was

then placed in a sealed vial and added with 3 mL of a freshly prepared HCl/AcOEt solution. The mixture was stirred for 48h and then the solvent was removed under reduced pressure. The residue was washed several times with small portions of cold Et_2O affording the desired lipoguanidine compound as HCl salt.

(Z)-1-(4-aminobutyl)-3-(octadec-9-en-1-yl)guanidine ***(5a)***. Oil; yield 93%. ^{1}H NMR (400 MHz, CD_3OD) δ: 5.34 (t, J = 4.8 Hz, 2H), 3.27 (t, J = 6.5 Hz, 2H), 3.20 (t, J = 7.2 Hz, 2H), 2.98 (t, J = 7.3 Hz, 2H), 2.09 – 1.96 (m, 4H), 1.78 – 1.66 (m, 4H), 1.59 (d, J = 7.3 Hz, 2H), 1.38 – 1.28 (m, 22H), 0.90 (t, J = 6.6 Hz, 3H). ^{13}C NMR (101 MHz, CD_3OD) δ: 157.4, 130.9, 130.8, 45.3, 42.7, 41.9, 40.3, 33.0, 30.8, 30.8, 30.8, 30.6, 30.6, 30.4, 30.4, 30.3, 30.3, 30.2, 30.0, 28.5, 28.1, 28.1, 27.8, 27.5, 26.9, 25.7, 23.7, 14.5. LRMS m/z (ES+) m/z: 381.

(Z)-1-(8-aminooctyl)-3-(octadec-9-en-1-yl)guanidine ***(5b)***. Oil; yield 57%. ^{1}H NMR (400 MHz, CD_3OD) δ: 5.34 (t, J = 4.9 Hz, 2H), 3.19 (dt, J = 7.2, 3.6 Hz, 4H), 2.93 (t, J = 7.7 Hz, 2H), 2.07 – 1.97 (m, 4H), 1.70 – 1.65 (m, 2H), 1.59 (d, J = 8.1 Hz, 4H), 1.40 – 1.29 (m, 30H), 0.91 (d, J = 6.2 Hz, 3H). ^{13}C NMR (101 MHz, CD_3OD) δ: 157.4, 130.9, 130.8, 65.1, 42.6, 40.8, 39.7, 33.0, 30.8, 30.7, 30.6, 30.5, 30.4, 30.3, 30.3, 30.0, 30.0, 29.9, 28.5, 28.1, 28.1, 27.7, 27.6, 27.3, 23.7, 14.5. LRMS m/z (ES+) m/z: 437.

1-(4-aminobutyl)-3-((9Z,12Z)-octadeca-9,12-dien-1-yl)guanidine ***(5c)***. Oil; yield 94%. ^{1}H NMR (400 MHz, CD_3OD) δ: 5.42 – 5.27 (m, 4H), 3.26 (q, J = 5.3 Hz, 2H), 3.19 (t, J = 7.2 Hz, 2H), 2.98 (t, J = 7.3 Hz, 2H), 2.78 (t, J = 6.4 Hz, 2H), 2.06 (q, J = 6.6 Hz, 4H), 1.76 – 1.67 (m, 4H), 1.60 (t, J = 6.9 Hz, 2H), 1.41 – 1.31 (m, 16H), 0.93 – 0.88 (m, 3H). ^{13}C NMR (101 MHz, CD_3OD) δ: 157.5,

130.9, 130.9, 129.1, 129.0, 45.3, 42.7, 41.9, 40.3, 32.7, 30.8, 30.6, 30.5, 30.3, 30.0, 28.5, 28.2, 27.8, 27.0, 26.5, 25.8, 23.6, 14.4. LRMS m/z (ES+) $C_{23}H_{47}N_4$ m/z: 379.

1-(8-aminooctyl)-3-((9Z,12Z)-octadeca-9,12-dien-1-yl)guanidine ***(5d).*** Oil; yield 56%. ^{1}H NMR (400 MHz, CD_3OD) δ: 5.31 (hept, J = 4.7 Hz, 4H), 3.18 (s, 4H), 2.91 (s, 2H), 2.75 (d, J = 6.2 Hz, 2H), 2.03 (p, J = 8.1, 7.2 Hz, 4H), 1.67 (s, 2H), 1.59 (s, 4H), 1.41 – 1.26 (m, 24H), 0.87 (d, J = 6.3 Hz, 3H). ^{13}C NMR (101 MHz, CD_3OD) δ: 157.5, 130.8, 130.7, 129.0, 128.9, 42.7, 40.2, 32.5, 30.6, 30.4, 30.3, 30.3, 30.2, 30.1, 30.0, 28.6, 28.1, 28.0, 27.7, 27.4, 26.4, 23.5, 14.3. HRMS (ESI): calcd for $C_{27}H_{56}N_4$ $(M + 2H)^+$ 218.2247, found 218.2245.

1-(4-aminobutyl)-3-((9Z,12Z,15Z)-octadeca-9,12,15-trien-1-yl)guanidine ***(5e).*** Oil; yield 47%. ^{1}H NMR (400 MHz, CD_3OD) δ: 5.43 – 5.25 (m, 6H), 3.29 – 3.23 (m, 4H), 3.19 (t, *J* = 7.2 Hz, 2H), 2.97 (t, *J* = 7.3 Hz, 2H), 2.83 – 2.79 (m, 2H), 2.13 – 2.04 (m, 4H), 1.77 – 1.67 (m, 6H), 1.39 – 1.33 (m, 12H), 0.98 (t, *J* = 7.5 Hz, 3H). ^{13}C NMR (101 MHz, CD_3OD) δ: 57.5, 132.7, 131.1, 130.9, 129.2, 128.9, 128.2, 45.3, 42.7, 41.9, 40.3, 32.7, 30.7, 30.6, 30.4, 30.4, 30.3, 30.3, 30.0, 28.5, 28.2, 27.8, 27.5, 27.0, 26.5, 26.4, 25.8, 23.7, 21.5, 14.6. HRMS (ESI): calcd for $C_{23}H_{45}N_4$ $(M + H)^+$ 377.3638, found 377.3623.

1-(8-aminooctyl)guanidine ***(5f).*** Oil; yield 44%. ^{1}H NMR (400 MHz, MeOD) δ: 3.19 (t, J = 7.0 Hz, 2H), 2.94 (q, J = 7.8, 5.8 Hz, 2H), 1.69 (t, J = 7.3 Hz, 2H), 1.60 (t, J = 6.9 Hz, 2H), 1.45 – 1.36 (m, 8H). ^{13}C NMR (101 MHz, MeOD) δ: 158.6, 42.4, 40.7, 29.9, 29.8, 29.7, 28.3, 27.4, 27.2. HRMS (ESI): calcd for $C_9H_{23}N_4$ $(M + H)^+$ 187.1917, found 187.1920.

1-(4-aminobutyl)-3-(9-aminononyl)guanidine ***(5g).*** Oil; yield 70%. ^{1}H NMR (400 MHz, CD_3OD) δ: 3.27 (t, *J* = 6.8 Hz, 2H), 3.20 (t, *J* = 7.2 Hz, 2H), 2.98 (t, *J* = 7.4 Hz, 2H), 1.83 – 1.63 (m, 4H), 1.66

– 1.54 (m, 2H), 1.43 – 1.25 (m, 14H), 0.94 – 0.86 (m, 3H). ^{13}C NMR (101 MHz, CD_3OD) δ: 157.3, 42.6, 41.9, 40.2, 33.0, 30.6, 30.6, 30.4, 30.3, 29.9, 27.7, 26.9, 25.6, 23.6, 14.4. HRMS (ESI): calcd for $C_{15}H_{35}N_4$ $(M + H)^+$ 271.28562, found 271.2855.

1-(4-aminobutyl)-3-(11-aminoundecyl)guanidine ***(5h).*** Oil; yield 88%. ^1H NMR (400 MHz, CD_3OD) δ: 3.28 (d, J = 6.3 Hz, 2H), 3.20 (t, J = 6.8 Hz, 2H), 2.98 (t, J = 6.7 Hz, 2H), 1.74 (dt, J = 25.7, 7.4 Hz, 4H), 1.60 (t, J = 6.9 Hz, 2H), 1.30 (d, J = 10.5 Hz, 18H), 0.92 – 0.87 (m, 3H). ^{13}C NMR (101 MHz, CD_3OD) δ: 157.3, 42.6, 41.9, 40.3, 32.9, 30.7, 30.6, 30.6, 30.6, 30.4, 30.3, 29.9, 27.7, 26.9, 25.6, 23.6, 14.4. HRMS (ESI): calcd for $C_{17}H_{39}N_4$ $(M + H)^+$ 299.31692, found 299.3169.

1-(4-aminobutyl)-3-hexadecylguanidine ***(5i).*** Oil; yield 69%. ^1H NMR (400 MHz, CD_3OD) δ: 3.27 (t, J = 6.8 Hz, 2H), 3.20 (t, J = 7.2 Hz, 2H), 3.02 – 2.96 (m, 2H), 1.81 – 1.65 (m, 4H), 1.65 – 1.56 (m, 2H), 1.30 (s, 26H), 0.94 – 0.88 (m, 3H) ppm. ^{13}C NMR (101 MHz, CD_3OD) δ: 157.4, 42.6, 41.9, 40.3, 33.0, 30.7, 30.7, 30.7, 30.7, 30.6, 30.4, 30.3, 29.9, 27.7, 26.9, 25.7, 23.7, 14.4. HRMS (ESI): calcd for $C_{21}H_{47}N_4$ $(M + H)^+$ 355.37952, found 355.3787.

1-(4-aminobutyl)-3-octadecylguanidine ***(5j).*** Oil; yield 68%. ^1H NMR (400 MHz, CD_3OD) δ: 3.26 (t, J = 6.7 Hz, 2H), 3.19 (t, J = 7.2 Hz, 2H), 2.98 (dd, J = 8.0, 6.8 Hz, 2H), 1.80 – 1.64 (m, 4H), 1.59 (q, J = 6.8 Hz, 2H), 1.29 (s, 30H), 0.93 – 0.87 (m, 3H). ^{13}C NMR (101 MHz, CD_3OD) δ: 157.4, 42.7, 41.9, 40.3, 33.1, 30.8, 30.8, 30.7, 30.7, 30.5, 30.4, 30.0, 27.8, 27.0, 25.7, 23.7, 14.5. HRMS (ESI): calcd for $C_{23}H_{51}N_4$ $(M + H)^+$ 383.41082, found 383.4105.

1-(8-(3-((Z)-octadec-8-en-1-yl)guanidino)octyl)-3-((Z)-octadec-9-en-1-yl)guanidine ***(6).*** Oil; yield 30%. ^1H NMR (400 MHz, $CDCl_3$) δ: 5.34 (t, J = 4.8 Hz, 4H), 3.21 – 3.17 (m, 8H), 2.11 – 1.96 (m, 8H), 1.59 (t, J = 6.9 Hz, 8H), 1.37 – 1.28 (m, 52H), 0.90 (s, 6H). ^{13}C NMR (101 MHz, $CDCl_3$) δ:

130.0, 129.8, 82.0, 79.2, 47.7, 43.9, 32.0, 29.8, 29.8, 29.7, 29.7, 29.6, 29.4, 29.4, 29.3, 29.2, 29.2, 28.3, 28.3, 27.3, 27.0, 26.9, 22.7, 14.2. HRMS (ESI): calcd for $C_{46}H_{93}N_6$ $(M + H)^+$ 728.7383, found 729.7439

Compound (7a). Oil; yield 57%. ^{1}H NMR (400 MHz, $CDCl_3$) δ: 8.19 (s, 1H), 3.27 (t, J = 7.5 Hz, 2H), 2.60 (t, J = 6.9 Hz, 2H), 1.52 – 1.43 (m, 2H), 1.38 (d, J = 7.1 Hz, 2H), 1.34 (s, 18H). ^{13}C NMR (101 MHz, $CDCl_3$) δ: 163.2, 155.8, 152.9, 82.6, 78.7, 41.2, 40.3, 30.1, 28.0, 27.8, 26.1.

Compound (7b). Oil; yield 76%. ^{1}H NMR (400 MHz, $CDCl_3$) δ: 11.48 (s, 1H), 8.27 (t, J = 5.2 Hz, 1H), 3.38 (td, J = 7.3, 5.2 Hz, 2H), 2.67 (t, J = 7.0 Hz, 2H), 1.54 (t, J = 7.0 Hz, 2H), 1.48 (d, J = 3.5 Hz, 18H), 1.46 – 1.41 (m, 2H), 1.37 – 1.27 (m, 8H). ^{13}C NMR (101 MHz, $CDCl_3$) δ: 163.8, 156.2, 153.5, 83.1, 79.3, 42.3, 41.1, 33.8, 29.4, 29.3, 29.1, 28.4, 28.2, 26.9.

(Z)-1-(4-guanidinobutyl)-3-(octadec-9-en-1-yl)guanidine (8a). Oil; yield 36%. ^{1}H NMR (400 MHz, CD_3OD) δ: 5.39 – 5.30 (m, 2H), 3.24 (d, J = 5.7 Hz, 4H), 3.19 (t, J = 7.1 Hz, 2H), 2.03 (tt, J = 8.5, 4.4 Hz, 4H), 1.67 (p, J = 3.2 Hz, 4H), 1.60 (t, J = 7.0 Hz, 2H), 1.39 – 1.27 (m, 22H), 0.94 – 0.86 (m, 3H).^{13}C NMR (101 MHz, CD_3OD) δ: 158.6, 157.4, 130.8, 130.8, 42.7, 42.1, 42.1, 33.0, 30.8, 30.8, 30.8, 30.6, 30.5, 30.4, 30.3, 30.3, 30.3, 30.0, 28.1, 28.1, 27.7, 27.1, 27.0, 23.7, 14.5. HRMS (ESI): calcd for $C_{24}H_{51}N_6$ $(M + H)^+$ 423.4169, found 423.4149

(Z)-1-(8-guanidinooctyl)-3-(octadec-9-en-1-yl)guanidine (8b). Oil; yield 56%. ^{1}H NMR (400 MHz, CD_3OD) δ: 5.34 (t, J = 4.8 Hz, 2H), 3.18 (q, J = 6.4 Hz, 6H), 2.06 – 2.00 (m, 2H), 1.58 (d, J = 7.5 Hz, 6H), 1.42 – 1.28 (m, 32H), 0.89 (t, J = 6.6 Hz, 3H). ^{13}C NMR (101 MHz, CD_3OD) δ: 159.2, 156.8, 130.9, 130.8, 42.6, 42.5, 33.0, 30.8, 30.7, 30.6, 30.5, 30.4, 30.3, 30.3, 30.2, 30.0, 29.9, 29.8, 28.1, 27.7, 27.6, 23.7, 14.4. HRMS (ESI): calcd for $C_{28}H_{59}N_6$ $(M + H)^+$ 479.47957, found 479.4789.

1-(4-guanidinobutyl)-3-((9Z,12Z)-octadeca-9,12-dien-1-yl)guanidine ***(8c).*** Oil; yield 55%. ^{1}H NMR (400 MHz, CD_3OD) δ: 5.43 – 5.24 (m, 4H), 3.24 (d, J = 5.6 Hz, 4H), 3.19 (t, J = 7.1 Hz, 2H), 2.77 (t, J = 6.3 Hz, 2H), 2.07 (tt, J = 9.8, 4.6 Hz, 4H), 1.67 (p, J = 3.0 Hz, 4H), 1.60 (t, J = 6.4 Hz, 2H), 1.35 (td, J = 14.7, 14.2, 6.1 Hz, 16H), 0.94 – 0.86 (m, 3H).^{13}C NMR (101 MHz, CD_3OD) δ: 158.6, 157.4, 130.9, 130.8, 129.1, 129.0, 42.7, 42.2, 42.1, 32.6, 30.7, 30.5, 30.5, 30.3, 30.3, 30.0, 28.2, 27.8, 27.2, 27.1, 26.5, 23.6, 14.4. LRMS m/z (ES+) m/z: 421.

1-(8-guanidinooctyl)-3-((9Z,12Z)-octadeca-9,12-dien-1-yl)guanidine ***(8d).*** Oil; yield 24%. ^{1}H NMR (400 MHz, CD_3OD) δ: 5.41 – 5.27 (m, 4H), 3.18 (td, J = 7.4, 6.7, 4.7 Hz, 6H), 2.78 (t, J = 6.3 Hz, 2H), 2.07 (dq, J = 9.5, 6.4, 5.2 Hz, 4H), 1.59 (d, J = 7.6 Hz, 6H), 1.38 (t, J = 10.8 Hz, 24H), 0.90 (q, J = 4.8, 3.2 Hz, 3H).^{13}C NMR (101 MHz, CD_3OD) δ: 157.5, 130.9, 130.8, 129.1, 129.0, 42.6, 42.5, 32.7, 30.7, 30.5, 30.5, 30.3, 30.3, 30.2, 30.0, 29.9, 28.2, 27.7, 27.7, 26.5, 23.6, 14.4. HRMS (ESI): calcd for $C_{28}H_{59}N_6$ $(M + 2H)^2$ 479.4796, found 479.4789.

1-decyl-3-(4-guanidinobutyl)guanidine ***(8e).*** Oil; yield 70%. ^{1}H NMR (400 MHz, CD_3OD) δ: 3.24 (q, J = 6.4, 5.7 Hz, 4H), 3.19 (t, J = 7.2 Hz, 2H), 1.67 (p, J = 3.2 Hz, 4H), 1.60 (t, J = 7.2 Hz, 2H), 1.42 – 1.26 (m, 14H), 0.90 (t, J = 6.8 Hz, 3H).^{13}C NMR (101 MHz, CD_3OD) δ 158.7, 157.5, 42.7, 42.2, 42.1, 33.0, 30.6, 30.4, 30.3, 30.0, 27.7, 27.2, 27.1, 23.7, 14.4. HRMS (ESI): calcd for $C_{16}H_{37}N_6$ $(M + H)^+$ 313.3074, found 313.3071.

1,1'-(octane-1,8-diyl)diguanidine ***(8f).*** Oil; yield 66%. ^{1}H NMR (400 MHz, MeOD) δ: 3.18 (t, J = 7.0 Hz, 4H), 1.60 (t, J = 7.0 Hz, 4H), 1.40 (d, J = 11.3 Hz, 8H). ^{13}C NMR (101 MHz, MeOD) δ: 158.6, 42.5, 30.0, 29.8, 27.5. HRMS (ESI): calcd for $C_{10}H_{25}N_6$ $(M + H)^+$ 229.2135, found 229.2132.

(Z)-1-(2-(bis(2-aminoethyl)amino)ethyl)-3-(octadec-9-en-1-yl)guanidine ***(10)***. Oil; yield 70%. ^{1}H NMR (400 MHz, $CDCl_3$) δ: 5.34 (td, *J* = 4.5, 2.2 Hz, 2H), 3.57 (q, *J* = 6.4, 5.9 Hz, 2H), 3.30 (s, 4H), 3.22 (t, *J* = 7.1 Hz, 2H), 3.10 (d, *J* = 32.5 Hz, 4H), 2.12 – 1.95 (m, 4H), 1.62 (p, *J* = 7.6 Hz, 2H), 1.44 – 1.27 (m, 24H), 0.93 – 0.86 (m, 3H). ^{13}C NMR (101 MHz, $CDCl_3$) δ: 130.9, 130.8, 53.4, 52.2, 42.5, 39.1, 37.2, 33.4, 30.9, 30.8, 30.8, 30.6, 30.4, 30.4, 30.3, 29.9, 28.1, 28.1, 27.8, 23.7, 14.4. HRMS (ESI): calcd for $C_{25}H_{55}N_6$ $(M + H)^+$ 439.4482, found 439.4476.

1-decyl-3-(4-(dimethylamino)butyl)guanidine ***(11a)***. Oil; yield 24%. ^{1}H NMR (400 MHz, CD_3OD) δ: 3.28 (s, 2H), 3.19 (d, *J* = 6.9 Hz, 4H), 2.90 (s, 6H), 1.83 (s, 2H), 1.73 – 1.51 (m, 4H), 1.45 – 1.19 (m, 14H), 0.93 – 0.83 (m, 3H). ^{13}C NMR (101 MHz, CD_3OD) δ: 157.2, 58.4, 43.7, 43.6, 42.6, 41.8, 32.9, 30.5, 30.3, 30.2, 29.8, 27.6, 26.8, 23.5, 22.8, 14.3. HRMS (ESI): calcd for $C_{17}H_{39}N_4^+$ $(M + H)^+$ 299.31692, found 299.3165.

1-decyl-3-(4-(pyrrolidin-1-yl)butyl)guanidine ***(11b)***. Oil; yield 84%. ^{1}H NMR (400 MHz, CD_3OD) δ: 3.67 (dt, *J* = 11.1, 5.8 Hz, 2H), 3.30 – 3.17 (m, 5H), 3.12 (qd, *J* = 7.4, 4.3 Hz, 2H), 2.14 (tt, *J* = 14.9, 7.4 Hz, 2H), 2.04 (pd, *J* = 8.5, 5.7 Hz, 2H), 1.84 (td, *J* = 11.0, 10.0, 6.0 Hz, 2H), 1.69 (p, *J* = 7.1 Hz, 2H), 1.60 (h, *J* = 7.1 Hz, 2H), 1.44 – 1.24 (m, 14H), 0.95 – 0.86 (m, 3H). ^{13}C NMR (101 MHz, CD_3OD) δ: 157.3, 55.5, 55.0, 42.6, 41.8, 33.0, 30.6, 30.6, 30.3, 30.3, 29.9, 27.7, 27.0, 24.1, 24.0, 23.6, 14.4. HRMS (ESI): calcd for $C_{19}H_{41}N_4$ $(M + H)^+$ 325.3326, found 325.3314

1-butyl-3-decylguanidine ***(11c)***. Oil; yield 85%. ^{1}H NMR (400 MHz, CD_3OD) δ: 3.19 (td, *J* = 7.1, 3.4 Hz, 4H), 1.58 (p, *J* = 7.2 Hz, 4H), 1.45 – 1.39 (m, 2H), 1.39 – 1.26 (m, 14H), 0.97 (t, *J* = 7.4 Hz, 3H), 0.90 (t, *J* = 6.8 Hz, 3H). ^{13}C NMR (101 MHz, CD_3OD) δ: 157.5, 42.5, 42.3, 33.0, 32.0, 30.6,

30.3, 30.3, 29.9, 27.6, 23.6, 20.8, 14.3, 13.9. HRMS (ESI): calcd for $C_{15}H_{34}N_3$ $(M + H)^+$ 256.2747, found 256.2744.

4-(3-decylguanidino)butanoic acid ***(11d)***. Oil; yield 43%. ^1H NMR (400 MHz, CD_3OD) δ: 3.24 (t, *J* = 7.2 Hz, 2H), 3.19 (t, *J* = 7.2 Hz, 2H), 2.40 (t, *J* = 7.1 Hz, 2H), 1.87 (p, *J* = 7.1 Hz, 2H), 1.60 (p, *J* = 7.2 Hz, 2H), 1.42 – 1.27 (m, 14H), 0.90 (t, *J* = 6.8 Hz, 3H). ^{13}C NMR (101 MHz, CD_3OD) δ: 176.7, 157.5, 42.6, 41.8, 33.0, 31.5, 30.6, 30.6, 30.3, 30.3, 29.9, 27.7, 25.2, 23.6, 14.3. HRMS (ESI): calcd for $C_{51}H_{32}O_2N_3$ $(M + H)^+$ 286.2489, found 286.2485.

1,3-didecylguanidine ***(11e)***. Oil; yield 85%. ^1H NMR (400 MHz, DMSO-d_6) δ: 7.62 (s, *J* = 5.1 Hz, 1H), 7.40 (s, 1H), 3.10 (dd, *J* = 12.6, 6.4 Hz, 1H). ^{13}C NMR (101 MHz, DMSO-d_6) δ: 155.7, 40.7, 31.3, 28.9, 28.7, 28.5, 26.0, 22.1, 13.9. HRMS (ESI): calcd for $C_{21}H_{46}N_3$ $(M + H)^+$ 340.36862, found 340.3679. HRMS (ESI): calcd for $C_{21}H_{46}N_3$ $(M + H)^+$ 340.3679, found 340.36862.

(Z)-1-decyl-3-(octadec-9-en-1-yl)guanidine ***(11f)***. Oil; yield 99%. ^1H NMR (400 MHz, DMSO-d_6) δ: 7.63 (t, *J* = 5.7 Hz, 2H), 7.40 (s, 2H), 5.32 (t, *J* = 5.0 Hz, 2H), 3.11 (t, *J* = 6.5 Hz, 5H), 1.97 (q, *J* = 6.4 Hz, 5H), 1.44 (t, *J* = 7.0 Hz, 5H), 1.24 (d, *J* = 4.9 Hz, 46H), 0.86 (d, *J* = 6.1 Hz, 6H). ^{13}C NMR (101 MHz, DMSO-d_6) δ: 155.7, 129.6, 40.7, 31.3, 29.1, 29.1, 29.0, 28.8, 28.7, 28.6, 28.6, 28.5, 26.5, 26.0, 22.1, 13.9. LRMS m/z (ES+) m/z: 450.

General procedure for the synthesis of the amides. To a solution of the appropriate amine (**3** or **12**) (1 mmol) and *N,N*-diisopropylethylamine (1.3 mmol) in CH_2Cl_2, was added dodecanoyl chloride or oleyl chloride (1 mmol) dropwise at 0 °C. The resulting reaction mixture was allowed to warm to

room temperature and stirred for 6 h. Saturated aqueous $NaHCO_3$ solution was then added, and the biphasic system was separated. The aqueous phase was extracted with CH_2Cl_2 (3 × 20 mL), and the organic phases were combined and dried over anhydrous $MgSO_4$, filtered and concentrated under reduced pressure. The residue was purified by flash column chromatography (SiO_2).

N-butyldodecanamide ***(13a)****.* Off-white solid; yield 99%. ^{1}H NMR (400 MHz, $CDCl_3$) δ: 7.74 (s, 1H), 3.00 (q, J = 6.4 Hz, 2H), 2.02 (t, J = 7.3 Hz, 2H), 1.46 (p, J = 7.0 Hz, 2H), 1.35 (p, J = 7.0 Hz, 2H), 1.27 (d, J = 7.5 Hz, 2H), 1.23 (s, 16H), 0.85 (t, J = 7.1 Hz, 6H). ^{13}C NMR (101 MHz, $CDCl_3$) δ: 171.8, 38.0, 35.4, 31.3, 29.0, 28.7, 28.6, 25.3, 22.1, 19.5, 13.9, 13.6. HRMS (ESI): calcd for $C_{16}H_{34}ON$ $(M + H)^+$ 256.26349, found 256.2633.

N-(4-aminobutyl)oleamide ***(13b)****.* Off-white solid; yield 99%. ^{1}H NMR (400 MHz, $CDCl_3$) δ: 5.32 (s, 2H), 3.00 (s, 2H), 2.69 – 2.66 (m, 2H), 2.06 – 1.94 (m, 6H), 1.46 (s, 4H), 1.23 (s, 18H), 0.85 (t, *J* = 6.7 Hz, 3H). HRMS (ESI): calcd for $C_{22}H_{51}ON_2$ $(M + H)^+$ 353.3526, found 353.3518.

N-(4-aminobutyl)dodecanamide ***(13c)****.* Off-white solid; yield 99%. ^{1}H NMR (400 MHz, $CDCl_3$) δ: 7.74 (t, 1H), 3.00 (s, 2H), 2.67 (m, 2H), 2.33 (m, 2H), 2.01 (t, *J* = 7.4 Hz, 2H), 1.40 (s, 6H), 1.23 (s, 16H), 0.85 (t, *J* = 6.4 Hz, 3H). HRMS (ESI): calcd for $C_{16}H_{35}ON_2$ $(M + H)^+$ 271.2744, found 271.2740.

General procedure for the synthesis of the urea. Under anhydrous conditions the appropriate isocyanate (0.53 mmol) and *N*-(tert-butoxycarbonyl)-1,4-butanediamine (**14**) (0.53 mmol) were mixed in toluene (5 mL) and heated at 60 °C. The reaction mixture was stirred for 6h in a N_2 atmosphere. Once the reaction was finished as indicated by TLC, the mixture was concentrated in vacuo and then purified by flash chromatography using hexane/EtOAc 9:1 as eluent.

*1-(4-aminobutyl)-3-octadecylurea (**15**).* Off-white solid; yield 18%. ^{1}H NMR (400 MHz, DMSO-d_6) δ: 7.81 (s, 2H), 5.85 (dd, J = 10.1, 5.1 Hz, 2H), 2.96 (dq, J = 12.9, 6.5 Hz, 4H), 2.81 – 2.72 (m, 2H), 1.51 (p, J = 8.0, 7.4 Hz, 2H), 1.42 – 1.30 (m, 4H), 1.23 (s, 30H), 0.87 – 0.83 (m, 3H). HRMS (ESI): calcd for $C_{23}H_{50}ON_3$ $(M + H)^+$ 384.39484, found 384.3946. The molecule recrystallised in all available deuterated solvents (CD_3OD, DMSO-d6) and thus it was not possible to record a ^{13}C NMR spectrum.

Minimum Inhibitory Concentration (MIC) determination. MICs of compounds were performed by microbroth dilution against a panel of multidrug-resistant Gram-positive (*Staphylococcus aureus* ATCC 9144 (MSSA), *Staphylococcus aureus* NCTC 13616 (EMRSA-15), *Staphylococcus aureus* NCTC 8325 (USA300), *Staphylococcus aureus* (SA1199B) *Enterococcus faecalis* NCTC 775 (VSE), *Enterococcus faecalis* NCTC 12201 (VRE), *Enterococcus faecium* NCTC 12204 (VRE)) and Gram-negative (*Klebsiella pneumoniae* NCTC 13368 (ESBL), *Klebsiella pneumoniae* M6, *Acinetobacter baumannii* AYE (ATCC BAA 1710), *Acinetobacter baumannii* ATCC 17978, *Pseudomonas aeruginosa* PAO1, *Pseudomonas aeruginosa* NCTC 13437, *Escherichia coli* NCTC 12923) bacteria in Tryptic Soy Broth (TSB). Compounds were made up in DMSO stocks, diluted down in TSB and added to the plate in 2-fold dilution series, 100 μL per well. Bacteria were grown overnight in TSB and back diluted to an optical density (OD) of 0.01.100 μL of bacterial culture was added to 100 μL of compound in a 96 well plate and incubated overnight at 37 °C. OD readings were measured with a FLUOstar Omega plate reader, and the MIC defined as the lowest concentration where visible growth was not observed (<0.1 OD_{600}). Equivalent concentrations of DMSO caused no inhibition of growth.

Synergistic assay using a fixed concentration of the lipoguanidine. Synergy was measured using an adapted MIC method where 2 compounds are tested in combination, with a second compound at a fixed concentration added to the first compound. In case of PMBN, it was used at a fixed concentration of 30 μg/mL. The first compounds were diluted down in TSB and added to the plate in 2-fold dilution series as before, 100 μL per well. Then, 50 μL per well of the second compound at a fixed concentration was added. 50 μL of bacterial culture at OD 0.02 was added to 150 μL of compounds in a 96 well plate and incubated overnight at 37 ˚C as before.

Synergism evaluation: checkboard assay. Synergy was measured using standard microdilution checkerboard assays under the same conditions as the MICs. Two-fold dilution series of each antibiotic were prepared in separate 96 well plates and then combined into one before addition of bacteria. The growth/no growth interface was determined using the same definition as the MIC. The fractional inhibitory concentration was calculated from the most synergistic well on the plate for three independent repeats and presented as the average +/− standard deviation. FIC is calculated as (MIC of compound A in combination with B/MIC of compound A alone) + (MIC of compound B in combination with A/MIC of compound B alone). MICs were determined on the same plates as the FICs to increase reproducibility. FIC values ≤0.5 were considered strongly synergistic and, consistent with a recent re-evaluation of FIC which stresses the importance of also measuring the MIC in the same microarray plate, values of 0.5 - <1 were weakly synergistic.

Haemolysis assay. Haemolysis was tested by incubating titrations of lipoguanidines in PBS with freshly collected human red blood cells for 1 h at 37 ˚C. Control wells were treated with 0.1% Triton-X-100 to ensure complete lysis, or PBS-only to represent no lysis. Samples were spun down to remove non-lysed cells and the OD_{550} of the supernatant was measured using a CLARIOstar plate

reader. The percentage of haemolysis was calculated as % haemolysis = (AP − AB) / (AC − AB) × 100, where AP is the absorbance value for a known peptide concentration, AC is the absorbance of the 0.1% Triton-X-100 control, AB is the absorbance of the PBS control. Data presented are averages and standard error from three biological replicate experiments.

Galleria mellonella toxicity assay. Galleria mellonella were purchased from Livefood UK Ltd. (Rooks Bridge, UK) and were stored in plastic boxes, containing sufficient sawdust, in fridge at 4 °C. *G. mellonella* were removed from fridge an hour prior to experimentation to allow time to acclimatize to room temperature. For experiments, it was considered that *Galleria mellonella* have an average weight of 300mg and an average haemolymph volume of 50 µL. The toxicity of the compounds was measured *in vivo* using the wax moth larvae, *Galleria mellonella* as previously described. In each sample, 10 larvae were injected through the front proleg with 10 µL of compound (50 mg/kg, 20 mg/kg, and 10 mg/kg) or PBS only. Larvae were incubated at 37 °C for 5 days, and at each 24 hours interval, the number of live and dead larvae were recorded. All 10 PBS-only larvae survived. Healthy larvae refer to larvae that don't look sick (*i.e.*, not significantly melanised). A larvae was considered dead when it failed to respond to physical stimuli.

NPN assay. The permeability of the outer membrane was analysed by using the NPN uptake assay. Bacteria were grown overnight in TSB at 37 °C with shaking at 200 rpm. Subculture from overnight cultures were prepared 1:20 for 2 hours at 37 °C with shaking at 200 rpm, allowing cultures to reach mid-logarithmic growth. The cells were collected by centrifugation (5-10 min, at 4500 rpm), resuspended in 5mL buffer A (5 mM HEPES buffer, 5 mM glucose, pH 7.2) and cells were again collected by centrifugation. Cells were resuspended in 2.5 mL buffer A. Then 100 µL cells and 50 µL of assay buffer containing 10 µM NPN were added to a 96-well optical-bottom black plate. Either

50 μL of a chemical compound, the positive control polymyxin b (PMB) or buffer A was added to each well and fluorescence was immediately monitored with a FLUOstar plate reader (ex 350nm, em 420nm) for 2 hours. For the time point, the NPN uptake was calculated using the following equations where: F_{obs} is the observed fluorescence of the sample. F_0 is the initial fluorescence of NPN with bacteria in the absence of any compound. F_{100} is the fluorescence of NPN with bacteria upon addition of 10 μg/mL PMB. NPN uptake factor = $(F_{obs} - F_0) / (F_{100} - F_0)$. The data resented is from the first time point measure, which is within 10 minutes of addition compounds and NPN to bacteria.

2. Microbiology data

Table S1. Evaluation of the antibacterial activity of lipoguanidines in combination with PMBN

	**MIC µg/mL**						
	K. pneumoniae		*A. baumannii*		*P. aeruginosa*		*E. coli*
	NCTC 13368	*M6*	*AYE*	*ATCC 17978*	*PAO1*	*NCTC 13437*	*NCTC 12923*
**5a**	>128	8	16	8	32-128	64	4
**5d**	32	16	16	8	16	32	4
**5g**	>128	>128	>128	>128	>128	>128	128
**5h**	>128	>128	>128	>128	>128	32	16
**5j**	>128	>128	32-64	32-64	>128	128	32-128
**8a**	128	8	16	8	32-128	64	4
**8d**	16	8	16	8	16	32	8

Table S2. Concentration of lipoguanidines **5** and **8** used on different bacterial strains in combination with rifampicin.

	**Concentration of lipoguanidines 5 and 8 (μg/mL) used in combination with rifampicin**						
	K. pneumoniae		*A. baumannii*		*P. aeruginosa*		*E. coli*
	NCTC 13368	*M6*	*AYE*	*ATCC 17978*	*PAO1*	*NCTC 13437*	*NCTC 12923*
**5a**	32	2	4	2	32	32	2
**5c**	4	2	2	2	4	32	1
**5d**	8	4	2	2	32	32	2
**5e**	32	32	32	32	32	32	8
**5f**	64	64	64	64	64	64	32
**5g**	64	64	64	64	64	64	32
**5h**	32	32	32	32	32	16	4
**5i**	32	8	16	2	32	32	16
**5j**	32	32	32	8	32	32	32
**8a**	32	4	8	2	32	32	2
**8c**	16	8	16	8	16	16	2
**8d**	8	8	4	2	32	32	2
**8e**	64	64	64	64	16	64	16
**8f**	64	64	64	64	64	64	64

Table S3. Evaluation of the antibacterial activity of lipoguanidines **11**, **13**, **15**

	MIC µg/mL						
	K. pneumoniae		*A. baumannii*		*P. aeruginosa*		*E. coli*
	NCTC 13368	*M6*	*AYE*	*ATCC 17978*	*PAO1*	*NCTC 13437*	*NCTC 12923*
**11a**	>128	>128	>128	>128	>128	>128	>128
**11b**	>128	>128	>128	>128	>128	>128	128
**11c**	32	32	16	16	128	>128	16
**11d**	>128	>128	>128	>128	>128	>128	>128
**11e**	>128	128	16	32	>128	>128	64
**11f**	>128	>128	>128	>128	>128	>128	>128
**13a**	>128	>128	>128	>128	>128	>128	>128
**13b**	>128	>128	>128	>128	>128	>128	>128
**13c**	>128	>128	>128	>128	>128	>128	>128
**15**	>128	>128	>128	>128	>128	>128	>128

Table S4. Concentration of lipoguanidines/amides/urea **11**, **13** and **15** used on different bacterial strains in combination with rifampicin

	**Concentration of lipoguanidines/amides/urea 11, 13 and 15 (μg/mL) used in combination with rifampicin**						
	K. pneumoniae		*A. baumannii*		*P. aeruginosa*		*E. coli*
	NCTC 13368	*M6*	*AYE*	*ATCC 17978*	*PAO1*	*NCTC 13437*	*NCTC 12923*
**11a**	64	64	64	64	64	64	64
**11b**	64	64	64	64	64	64	32
**11c**	32	32	32	32	32	32	32
**11d**	32	32	32	32	32	32	32
**11e**	32	32	32	32	32	32	32
**11f**	32	32	32	32	32	32	32
**13a**	32	32	32	32	32	32	32
**13b**	32	32	32	32	32	32	32
**13c**	32	32	32	32	32	32	32
**15**	64	64	64	64	64	64	64

Table S5. Synergistic study of **5g** with other antibiotics

Rifampicin MIC (μg/mL)							
	K. pneumoniae		*A. baumanii*		*P. aeruginosa*		*E. coli*
	NCTC 13368	*M6*	*AYE*	*ATCC 17978*	*PAO1*	*NCTC 13437*	*NCTC 12923*
Alone							
**Amplicillin**	>128	>128	>128	16	>128	>128	4
**Ceftazidime**	>128	0.25	>128	4	4	>128	0.25
**Doxycycline**	64	4	2	≤0.125	32	>128	2
**Tobramycin**	32	4	>128	4	2	>128	8
In combination with **5g**[a]							
**Amplicillin**	>128	>128	>128	8	>128	>128	4
**Ceftazidime**	>128	0.25	>128	4	2	>128	0.25
**Doxycycline**	2	0.5	1	≤0.125	2	16	≤0.125
**Tobramycin**	32	1	>128	4	4	>128	8

MIC reduced ≥ 4-fold (green)

No change to MIC or with 2-fold difference (yellow)

[a] 32 μg/mL of lipoguanidine was added

3. Biological data

Table S4. Haemolytic activity of lipoguanidines

	Haemolytic activity (µg/mL)			
	HC_{10}		HC_{50}	
	rep 1	*rep 2*	*rep 1*	*rep 2*
**5a**	~40	~38	~165	~105
**5c**	~48	~45	~90	~85
**5e**	~5	~5	~15	~10
**5g**	~380	~450	>512	>512
**5h**	~55	~55	100	~175
**5i**	~25	~25	~80	~160
**5j**	~175	~75	>512	>512
**8a**	~145	~115	>512	~315
**8e**	128-256	128-256	256-512	256-512
**Novobiocin**	>512	>512	>512	>512

HC_{10}, HC_{50} - Concentration inducing 10% or 50% haemolysis, respectively

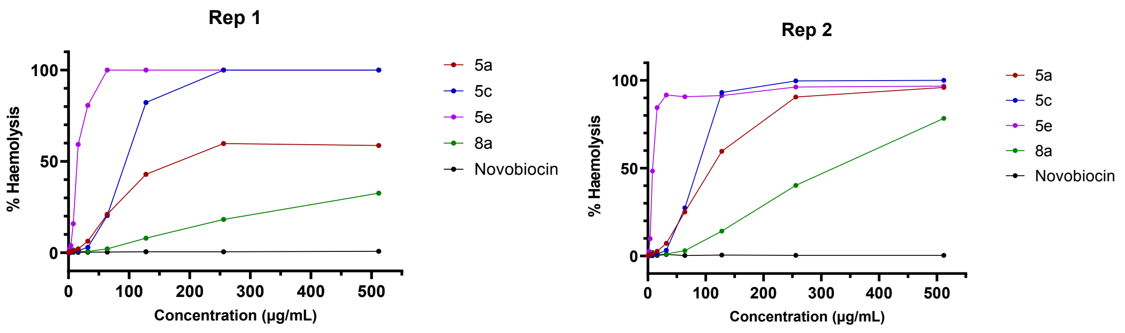

Figure S1. Dose-response curve of haemolytic activity of lipoguanidine compounds **5a**, **5c**, **5e**, **8a**.

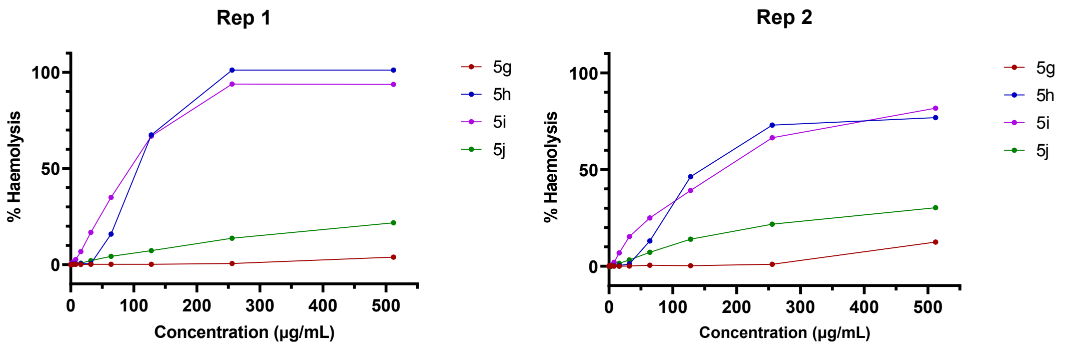

Figure S2. Dose-response curve of haemolytic activity of lipoguanidine compounds **5g**, **5h**, **5i** and **5j**.

Table S5. Toxicity of **5g** in *Galleria mellonella.* Survival counts of *Galleria mellonella* larvae post injection with **5g**

	24h	48h	72h	96h	120h
**PBS**	10	10	10	10	10
**5g 50mg/kg**	10	10	10	10	10
**5g 20mg/kg**	10	10	10	10	10
**5g 10mg/kg**	10	10	10	10	10

4. ^{1}H and ^{13}C NMR spectra

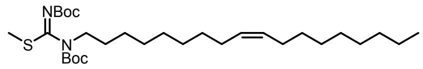

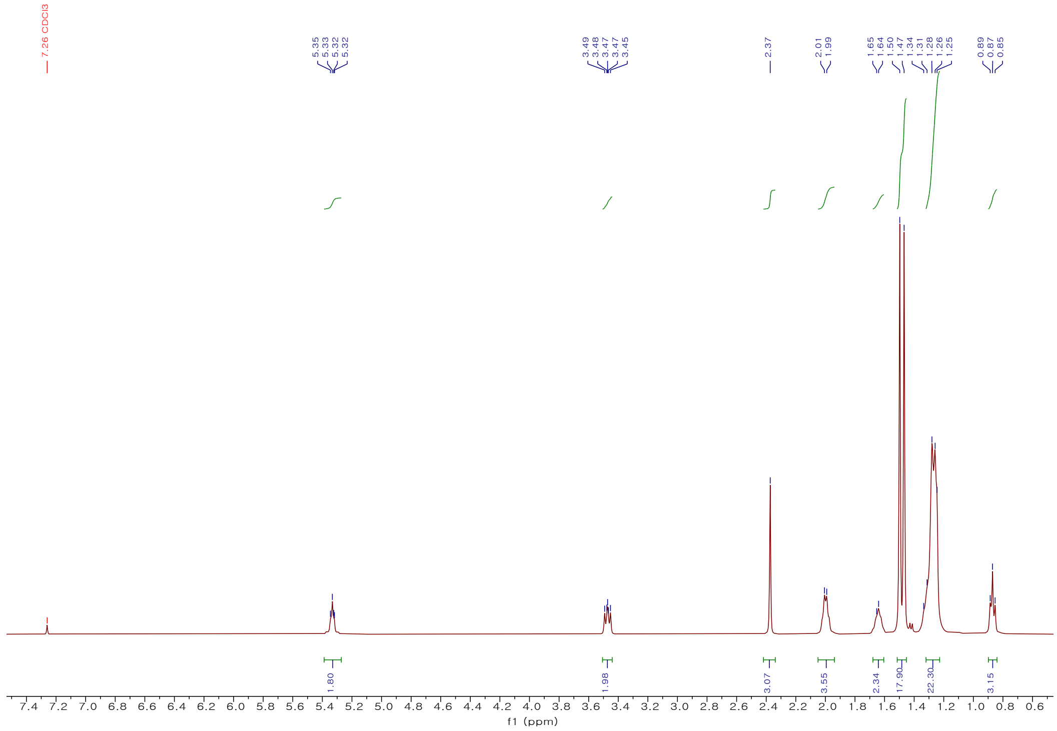

Figure S3. ^{1}H NMR spectrum of compound **2a** (400 MHz, $CDCl_3$)

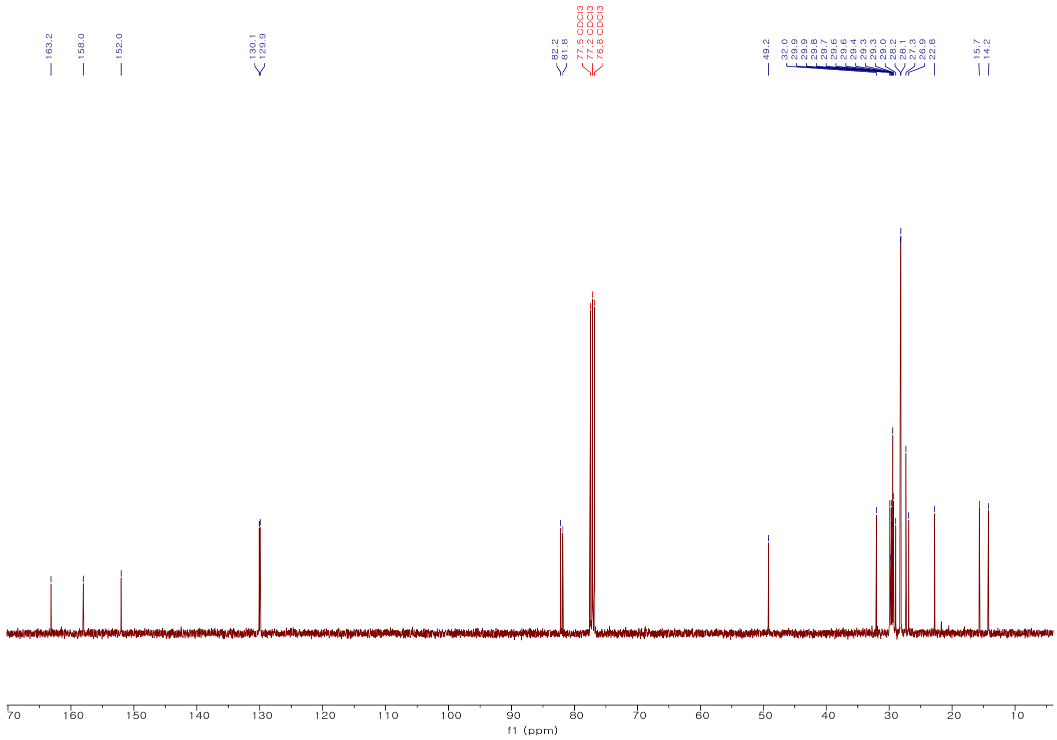

Figure S4. ^{13}C NMR spectrum of compound **2a** (101 MHz, $CDCl_3$)

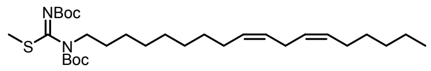

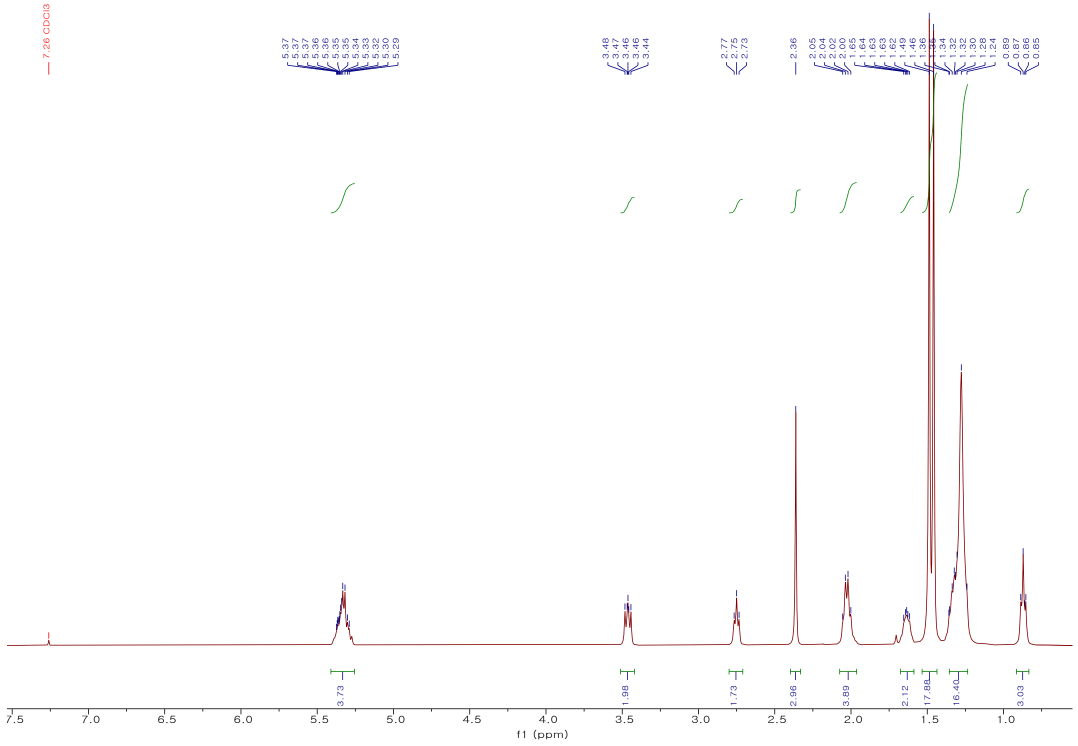

Figure S5. ^{1}H NMR spectrum of compound **2b** (400 MHz, $CDCl_3$)

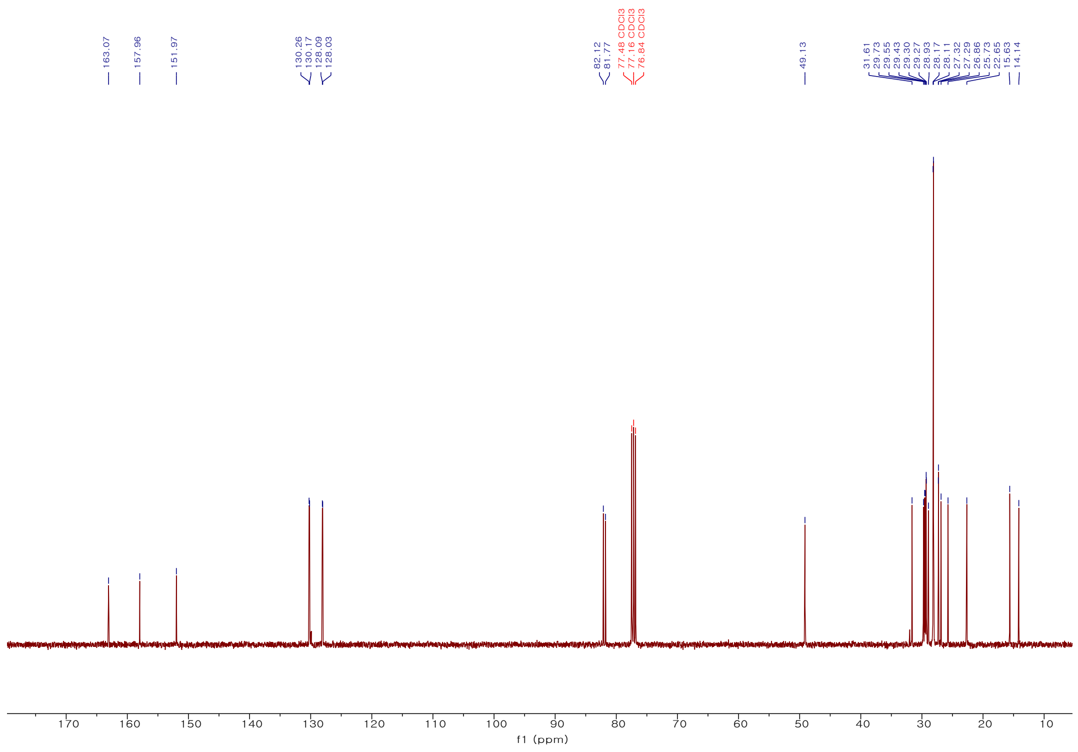

Figure S6. ^{13}C NMR spectrum of compound **2b** (101 MHz, $CDCl_3$)

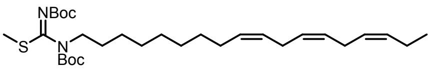

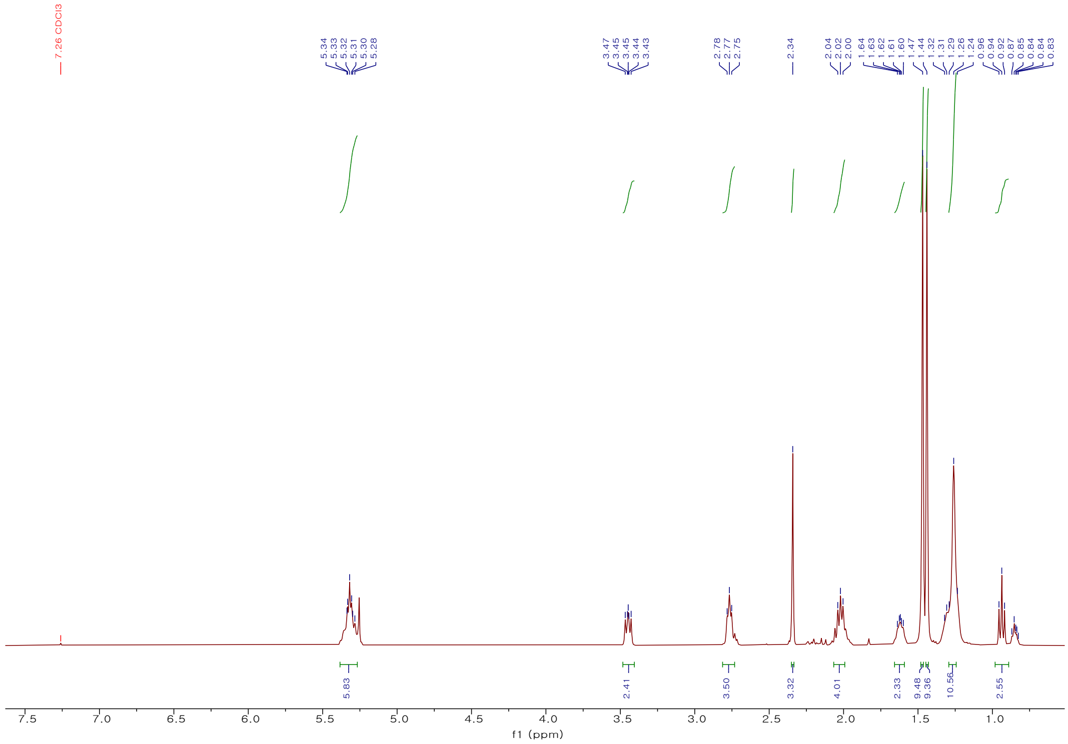

Figure S7. ^{1}H NMR spectrum of compound **2c** (400 MHz, $CDCl_3$)

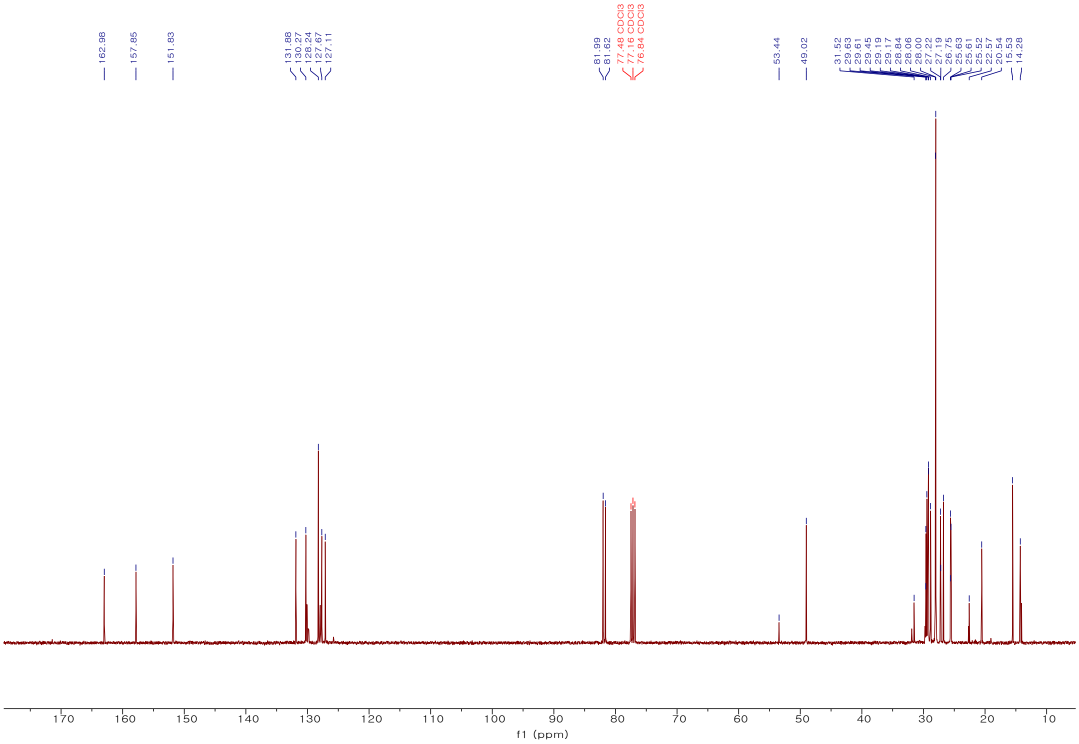

Figure S8. ^{13}C NMR spectrum of compound **2c** (101 MHz, $CDCl_3$)

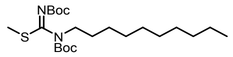

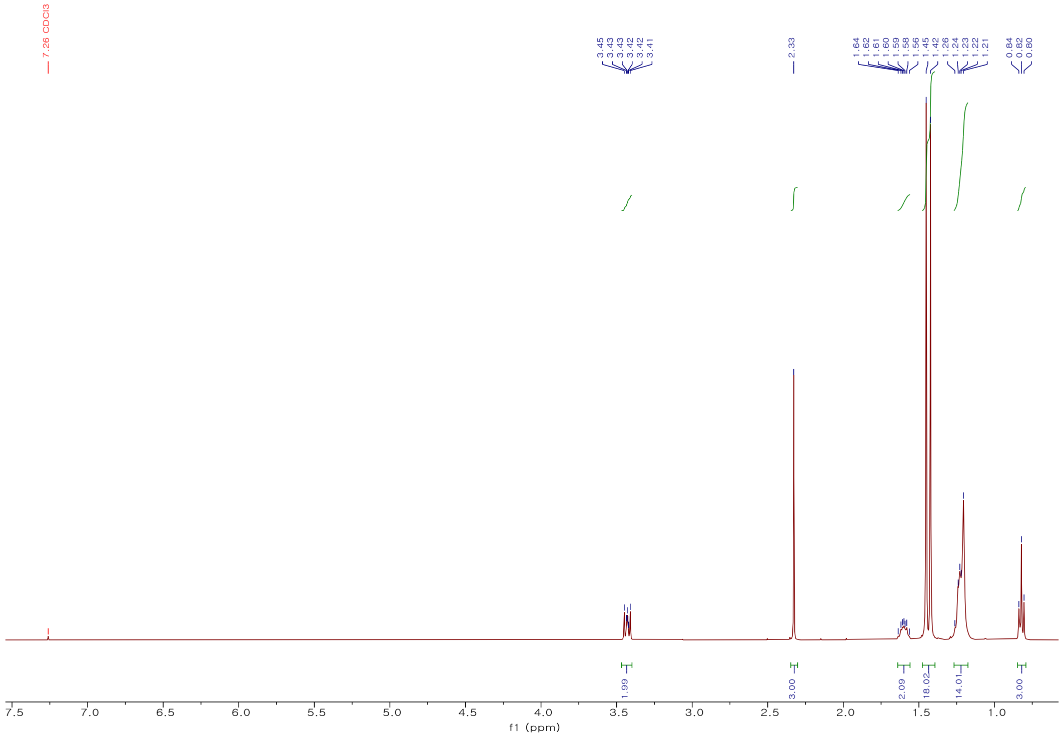

Figure S9. ^{1}H NMR spectrum of compound **2d** (400 MHz, $CDCl_3$)

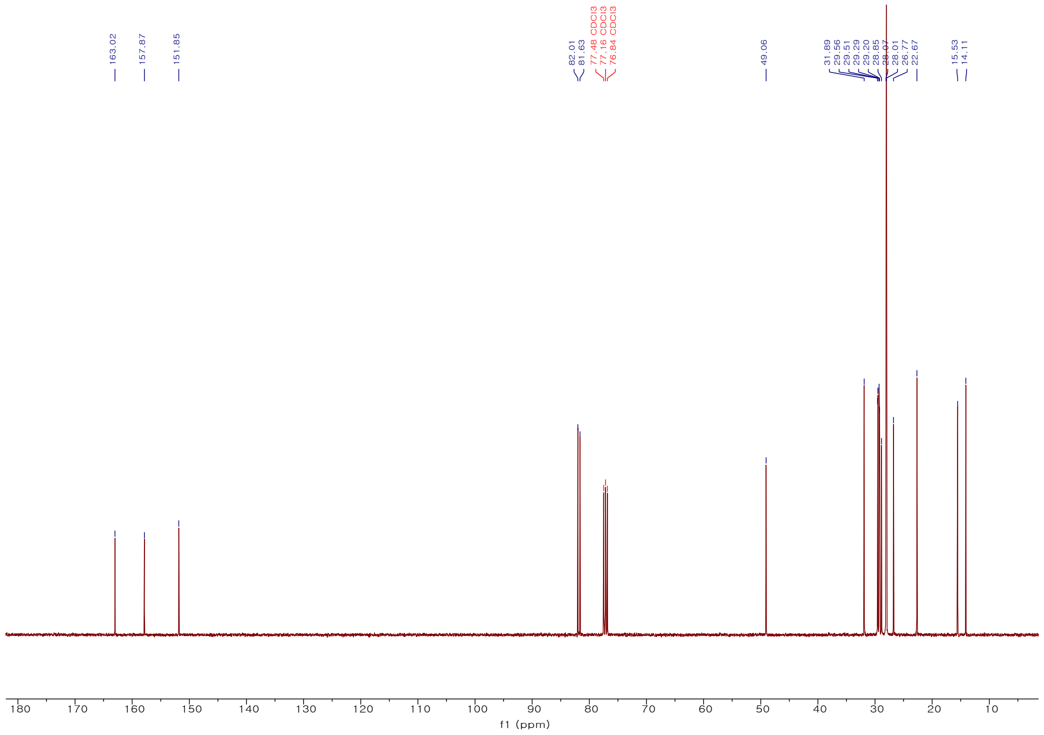

Figure S10. ^{13}C NMR spectrum of compound **2d** (101 MHz, $CDCl_3$)

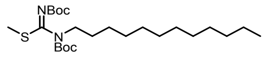

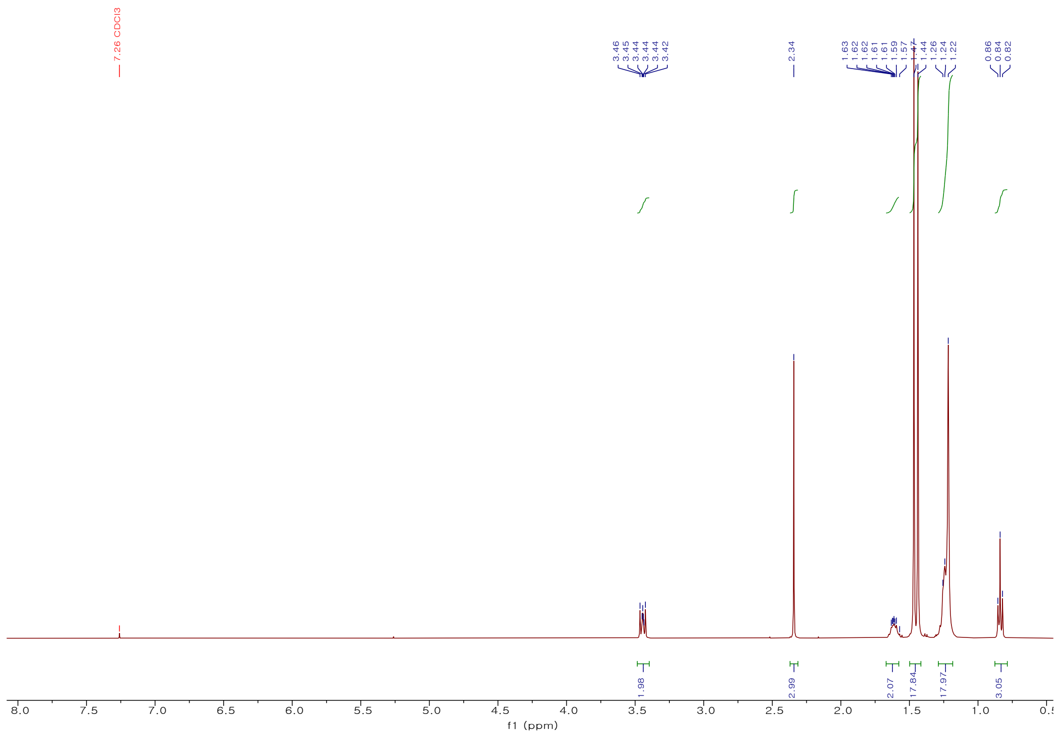

Figure S11. ^{1}H NMR spectrum of compound **2e** (400 MHz, $CDCl_3$)

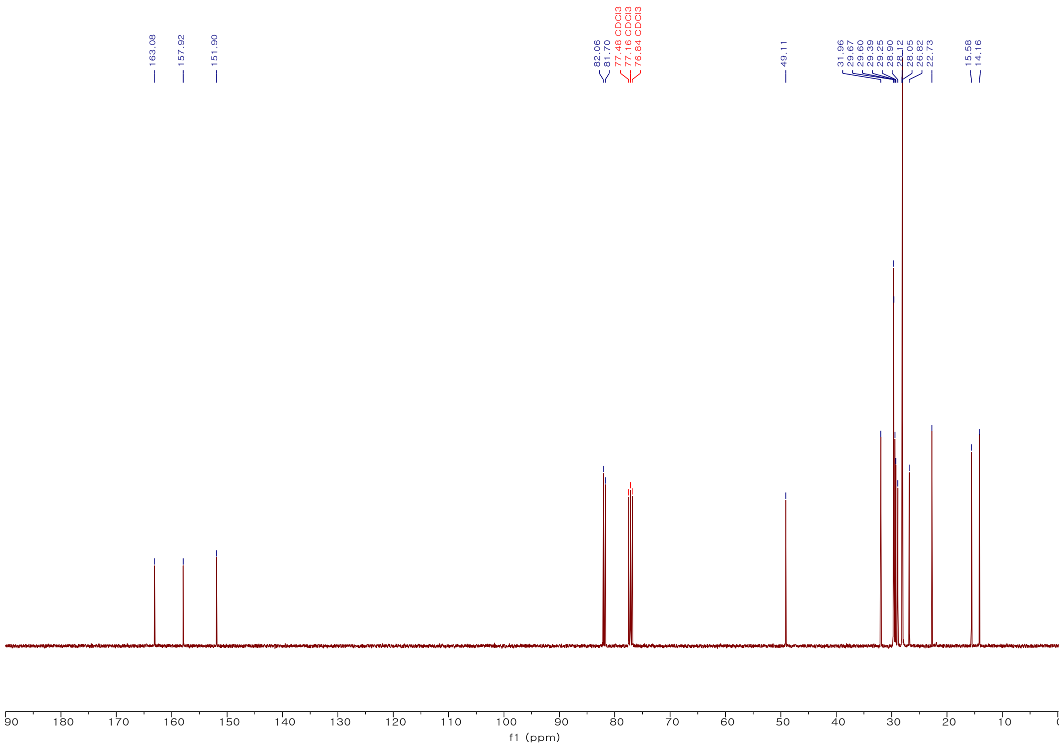

Figure S12. ^{13}C NMR spectrum of compound **2e** (101 MHz, $CDCl_3$)

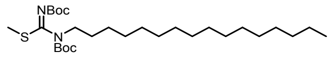

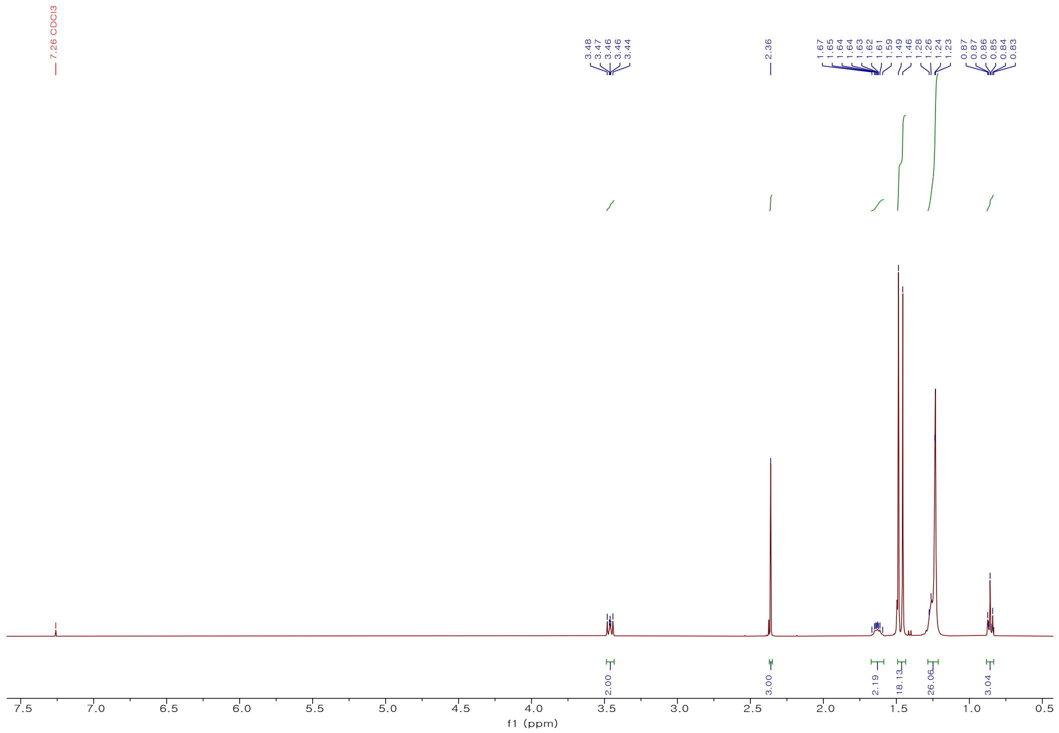

Figure S13. ^{1}H NMR spectrum of compound **2f** (400 MHz, $CDCl_3$)

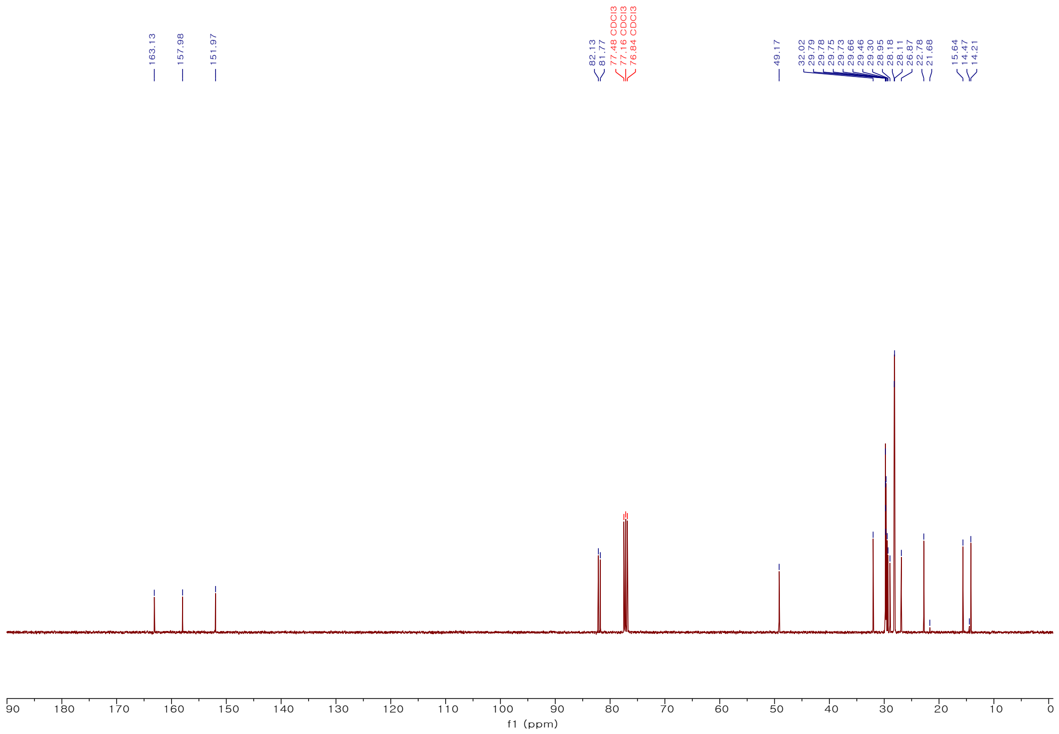

Figure S14. ^{13}C NMR spectrum of compound **2f** (101 MHz, $CDCl_3$)

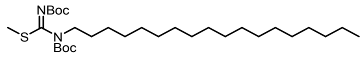

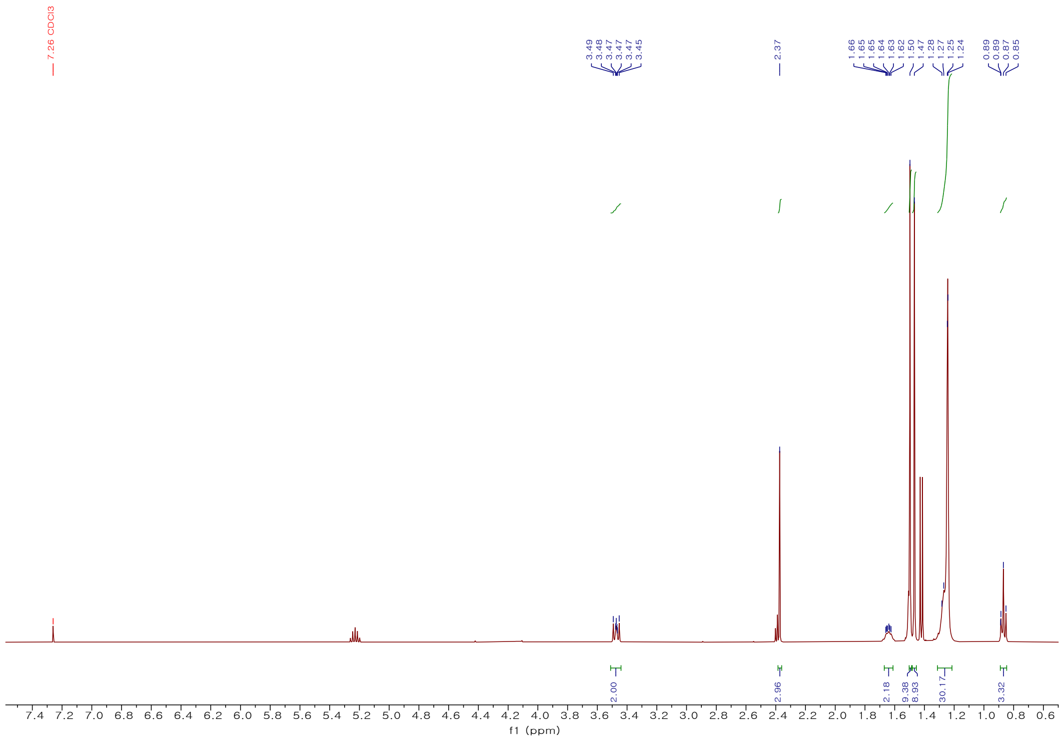

Figure S15. ^{1}H NMR spectrum of compound **2g** (400 MHz, $CDCl_3$)

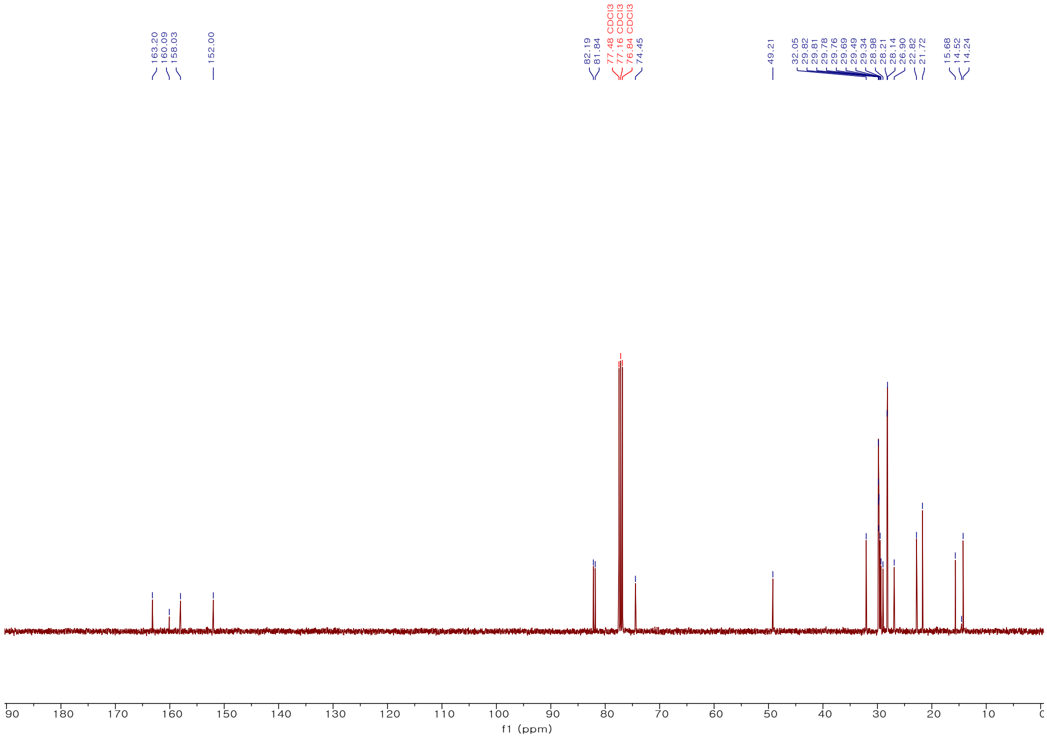

Figure S16. ^{13}C NMR spectrum of compound **2g** (101 MHz, $CDCl_3$)

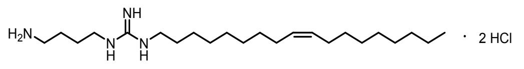

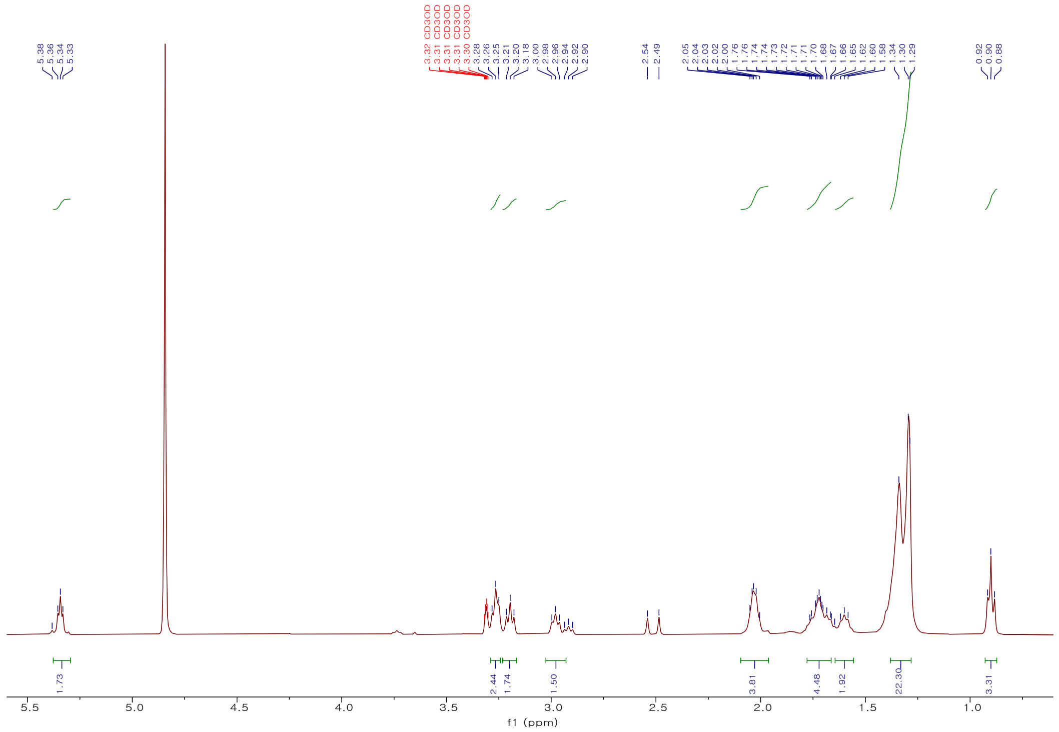

Figure S17. ^{1}H NMR spectrum of compound **5a** (400 MHz, CD_3OD)

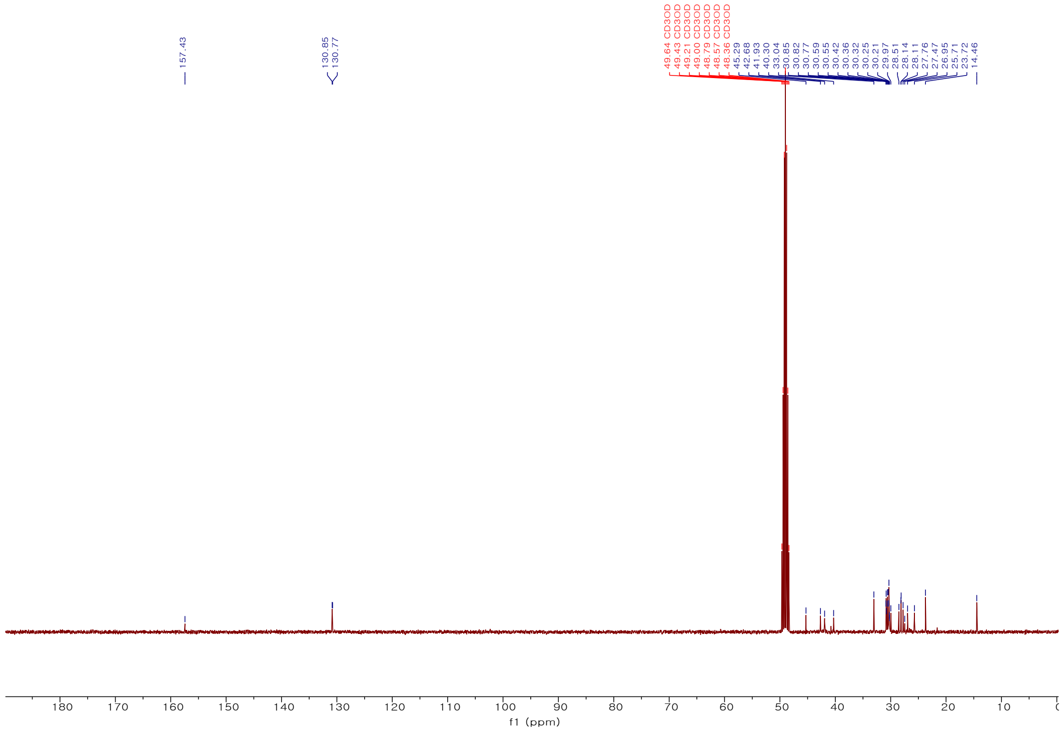

Figure S18. ^{13}C NMR spectrum of compound **5a** (101 MHz, CD_3OD)

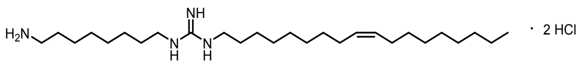

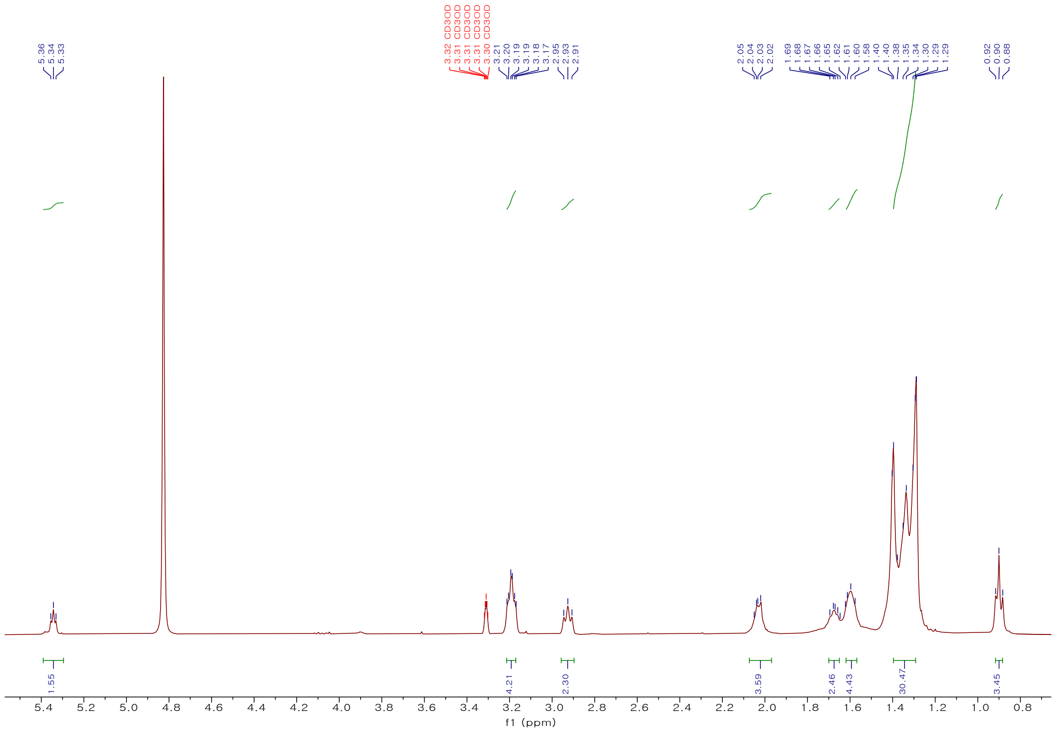

Figure S19. ^{1}H NMR spectrum of compound **5b** (400 MHz, CD_3OD)

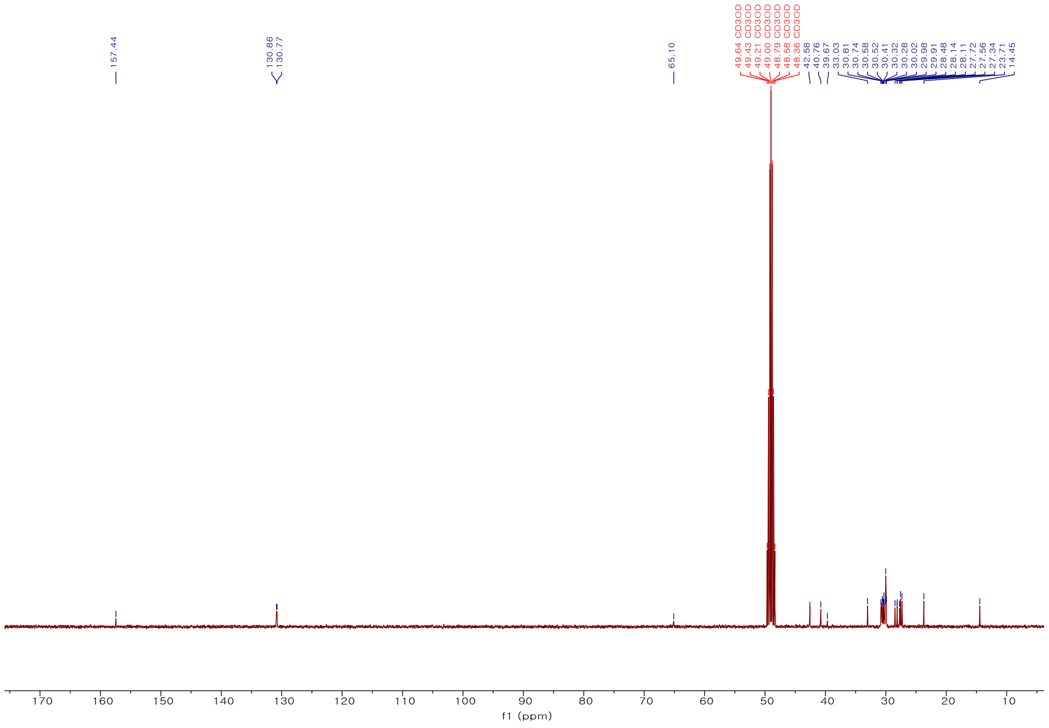

Figure S20. ^{13}C NMR spectrum of compound **5b** (101 MHz, CD_3OD)

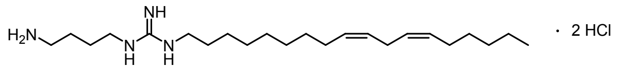

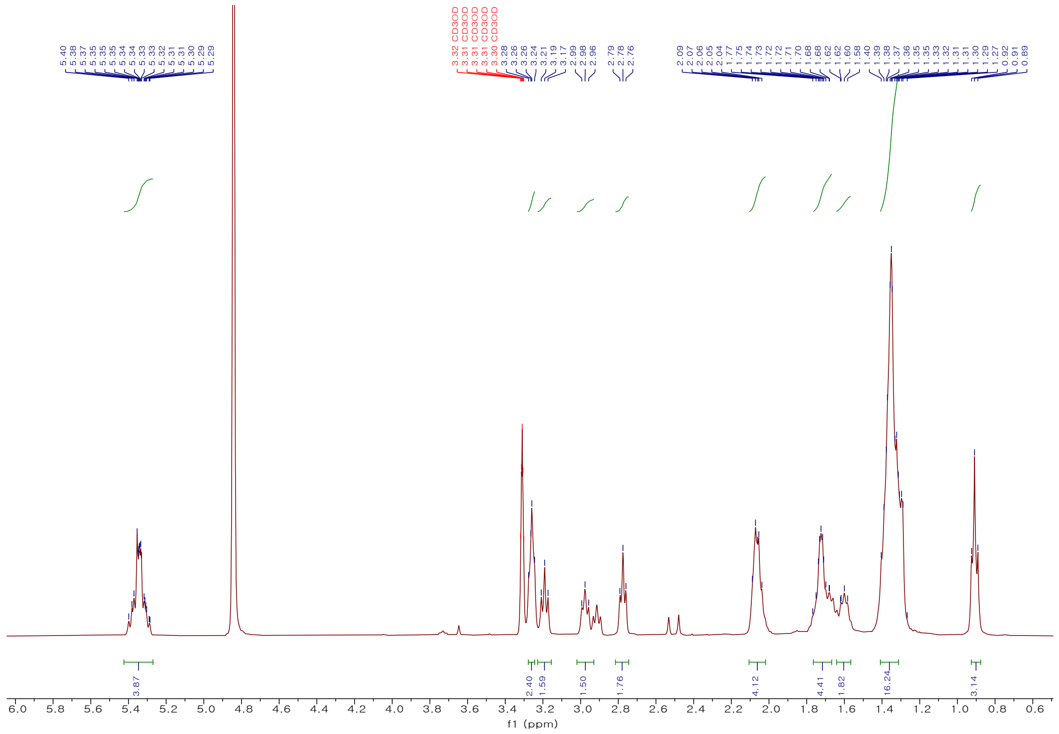

Figure S21. ^{1}H NMR spectrum of compound **5c** (400 MHz, CD_3OD)

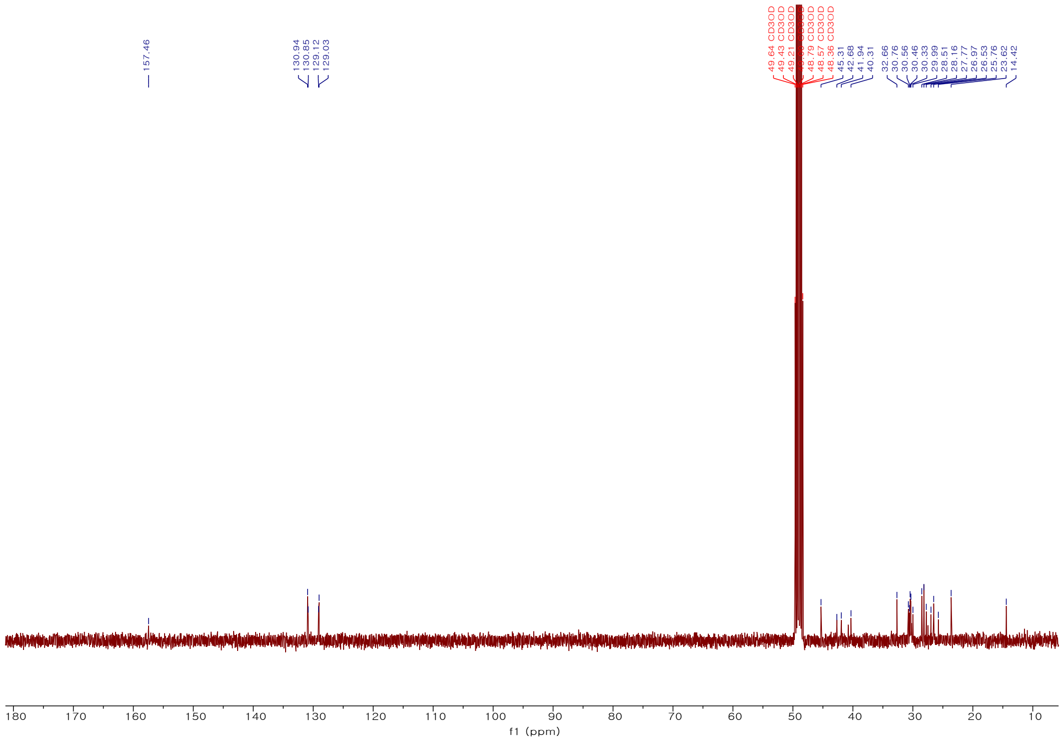

Figure S22. ^{13}C NMR spectrum of compound **5c** (101 MHz, CD_3OD)

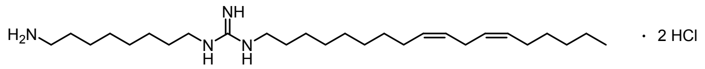

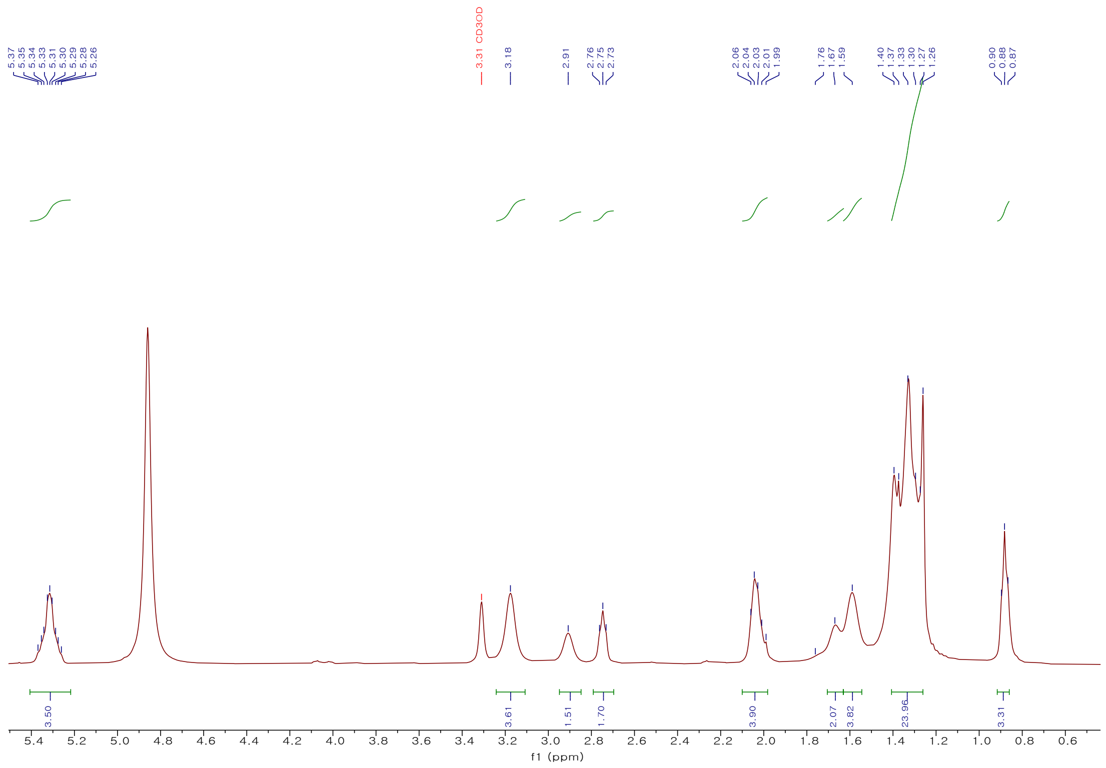

Figure S23. ^{1}H NMR spectrum of compound **5d** (400 MHz, CD_3OD)

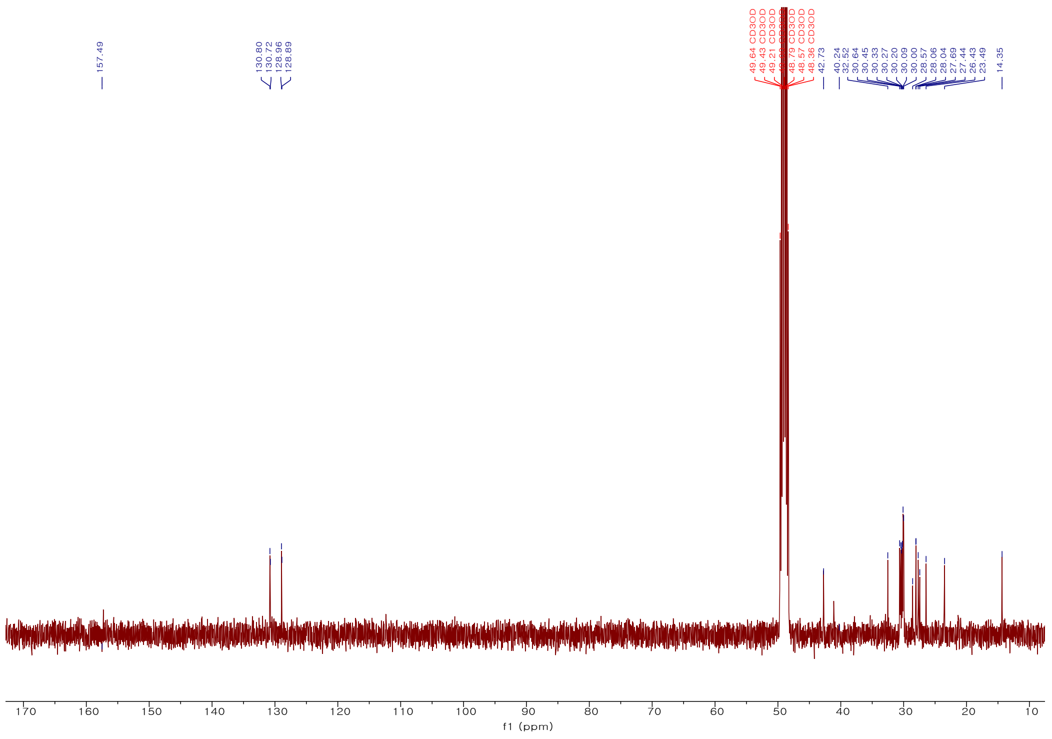

Figure S24. ^{13}C NMR spectrum of compound **5d** (101 MHz, CD_3OD)

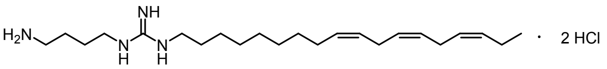

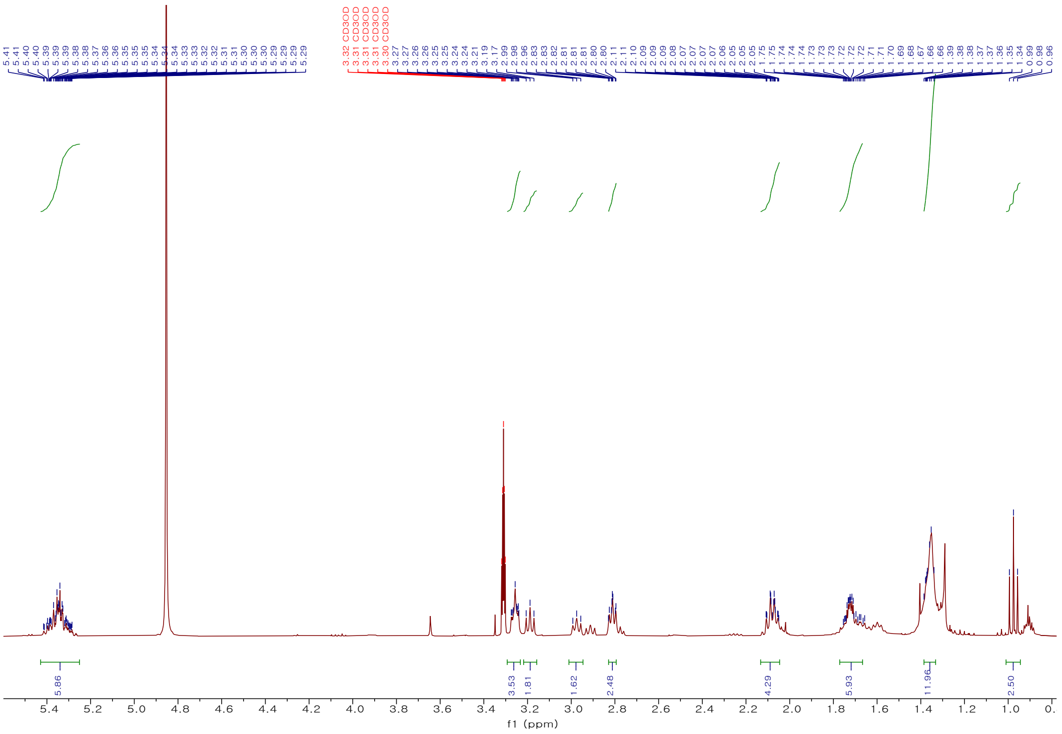

Figure S25. ^{1}H NMR spectrum of compound **5e** (400 MHz, CD_3OD)

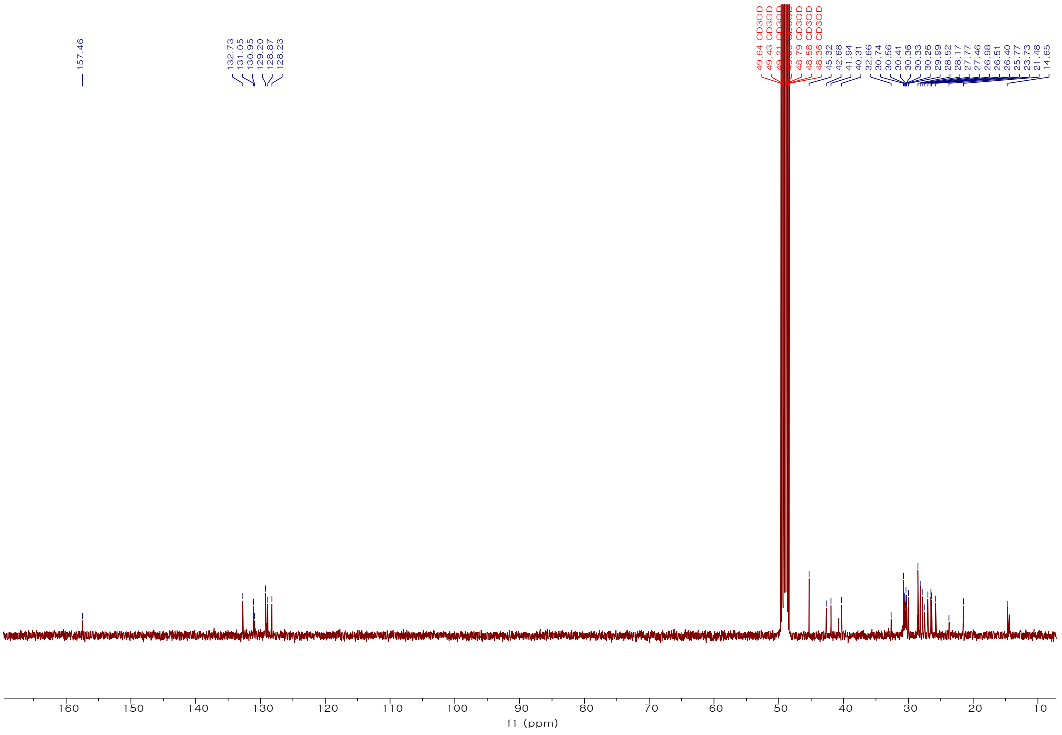

Figure S26. ^{13}C NMR spectrum of compound **5e** (101 MHz, CD_3OD)

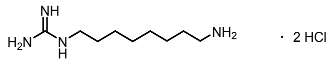

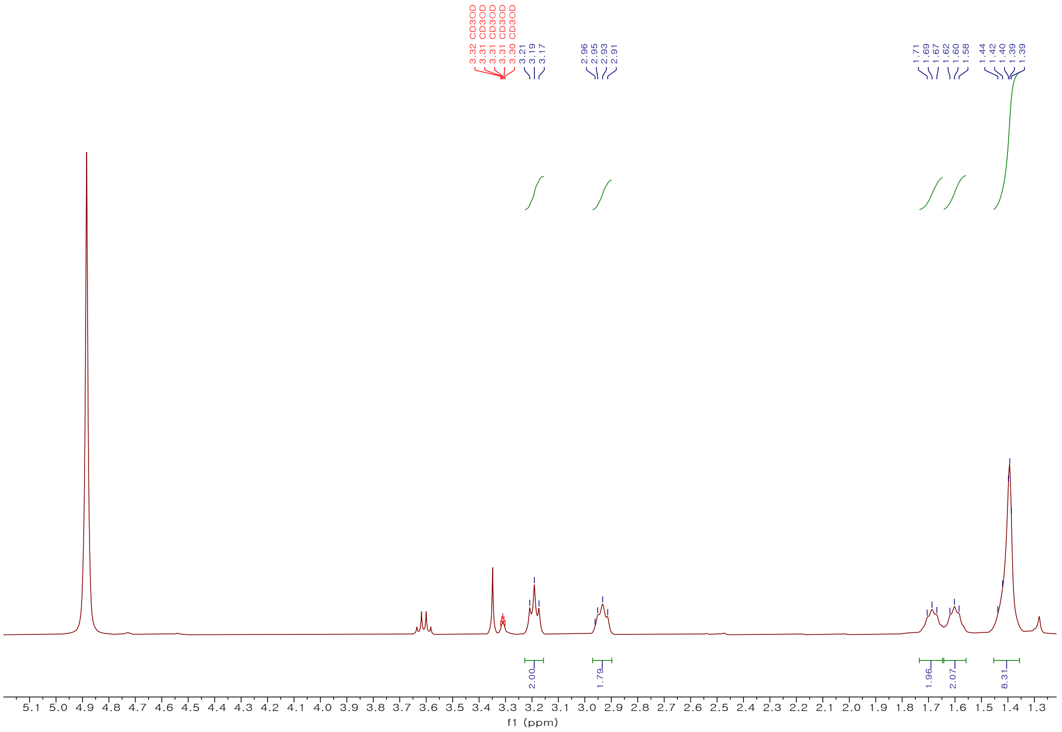

Figure S27. ^{1}H NMR spectrum of compound **5f** (400 MHz, CD_3OD)

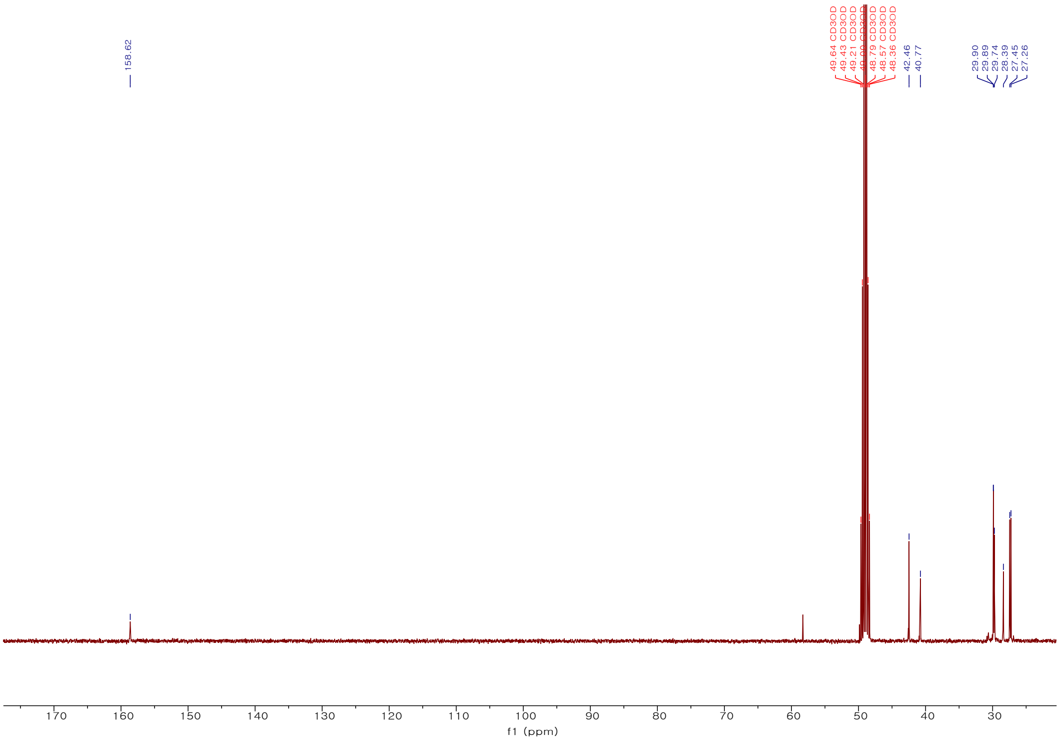

Figure S28. ^{13}C NMR spectrum of compound **5f** (101 MHz, MeOD)

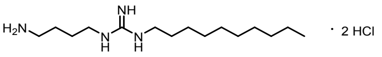

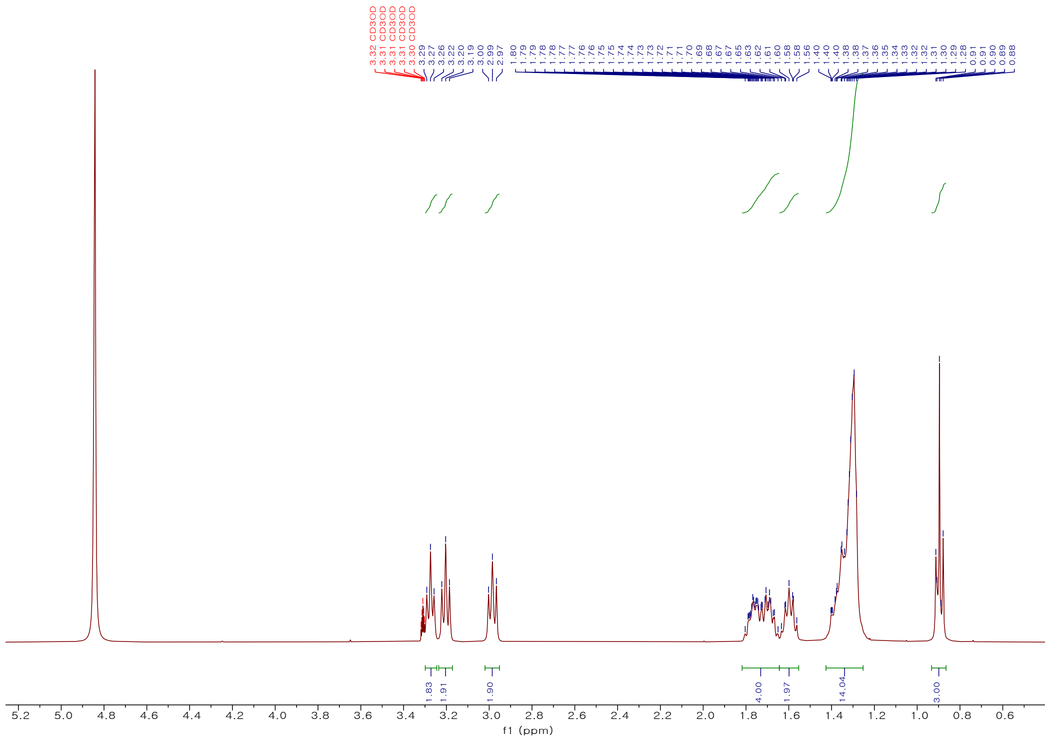

Figure S29. ^{1}H NMR spectrum of compound **5g** (400 MHz, CD_3OD)

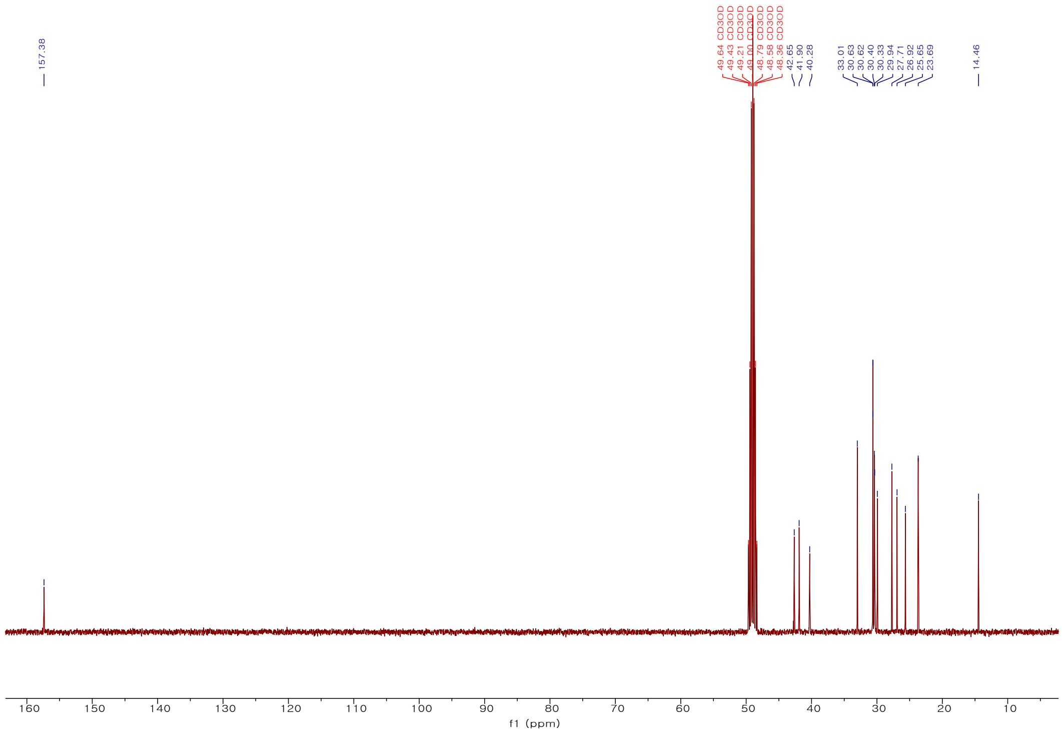

Figure S30. ^{13}C NMR spectrum of compound **5g** (101 MHz, CD_3OD)

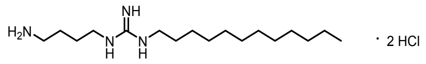

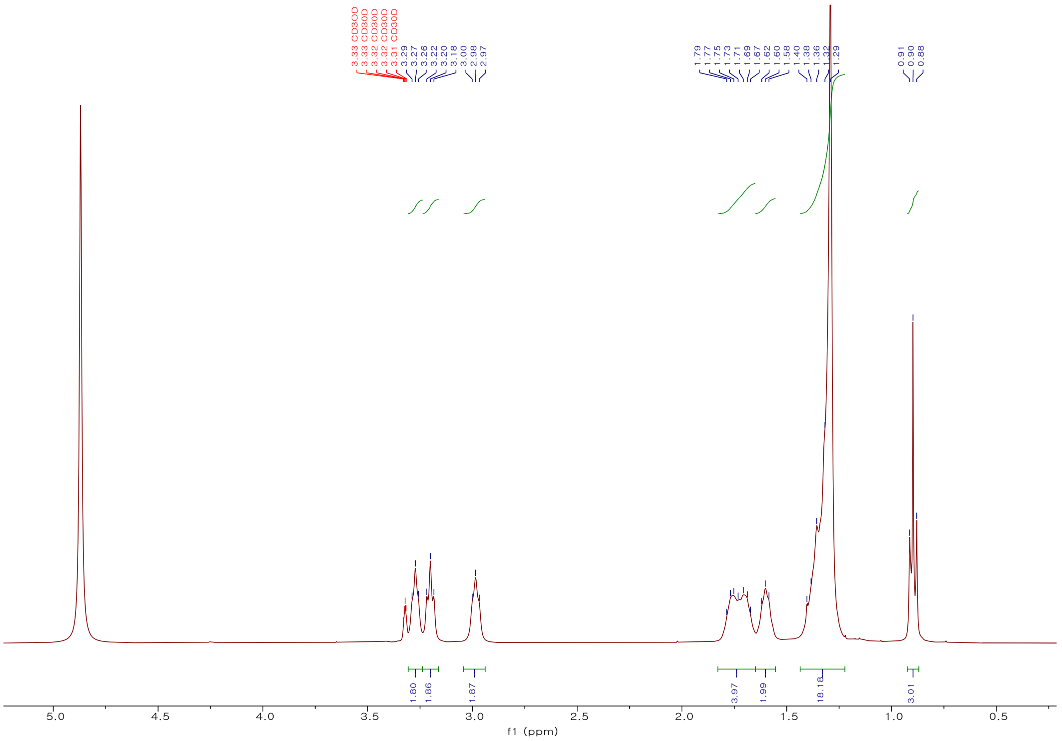

Figure S31. ^{1}H NMR spectrum of compound **5h** (400 MHz, CD_3OD)

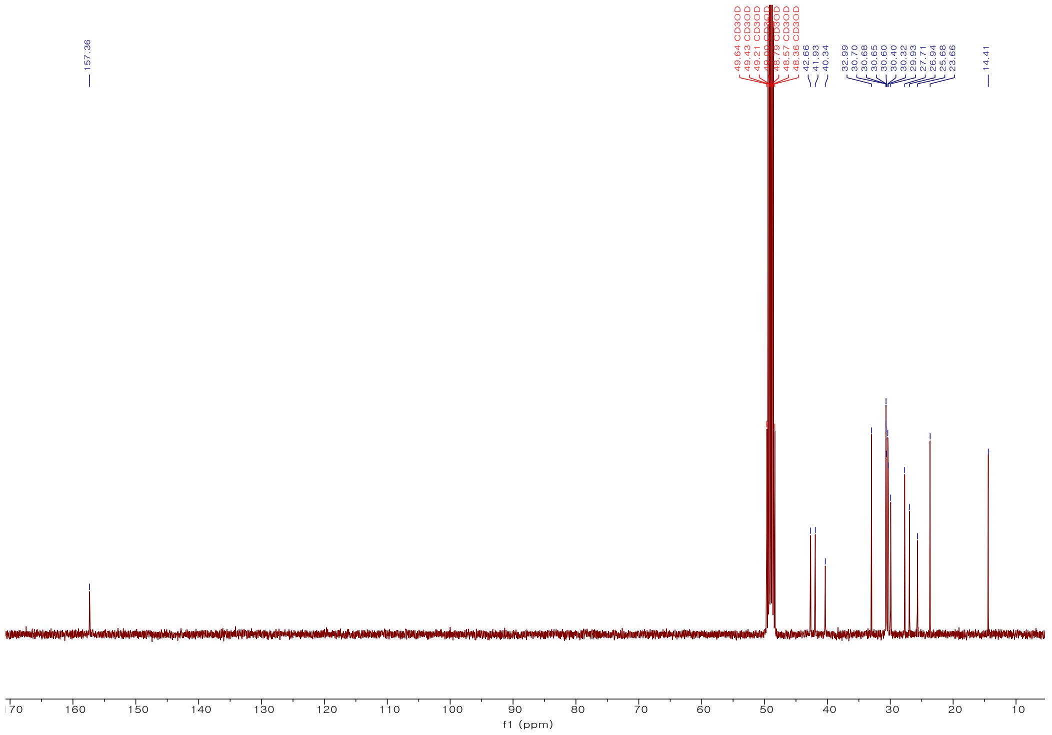

Figure S32. ^{13}C NMR spectrum of compound **5h** (101 MHz, CD_3OD)

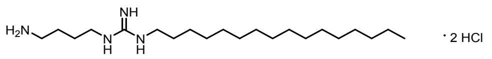

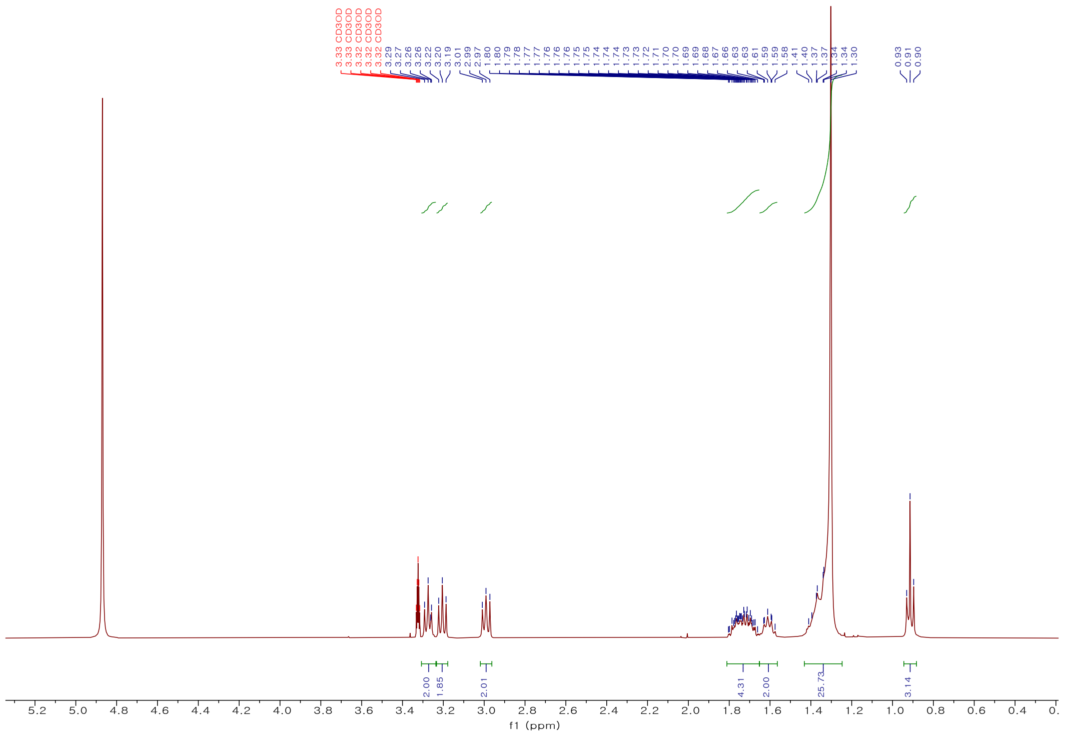

Figure S33. ^{1}H NMR spectrum of compound **5i** (400 MHz, CD_3OD)

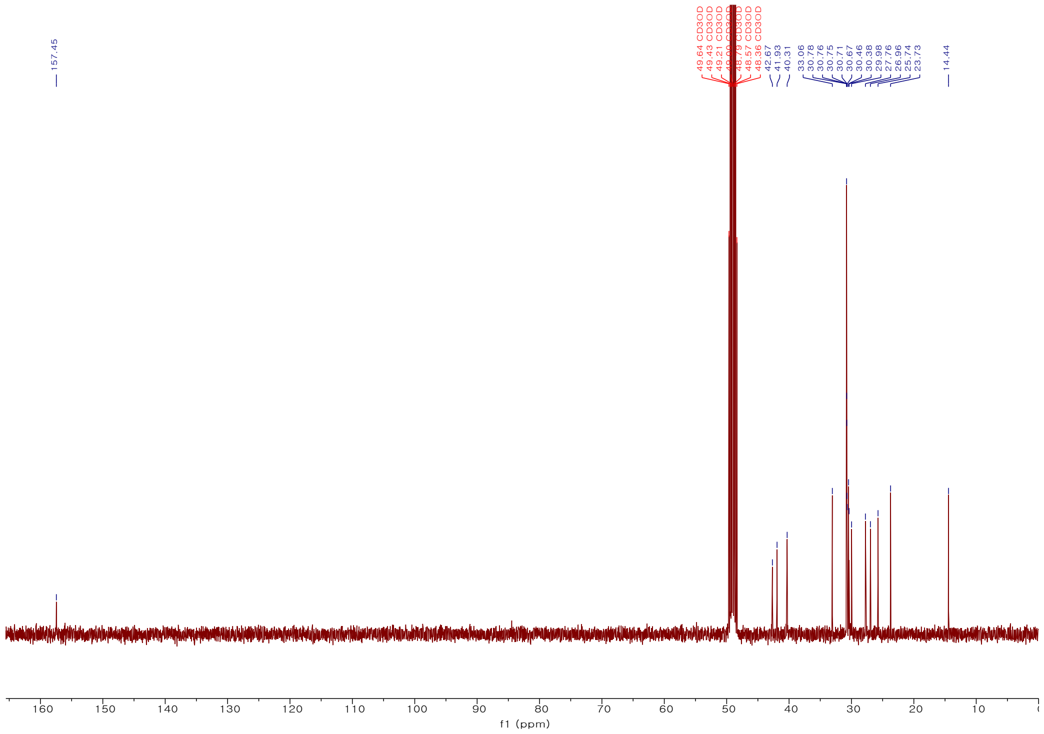

Figure S34. ^{13}C NMR spectrum of compound **5i** (101 MHz, CD_3OD)

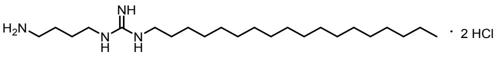

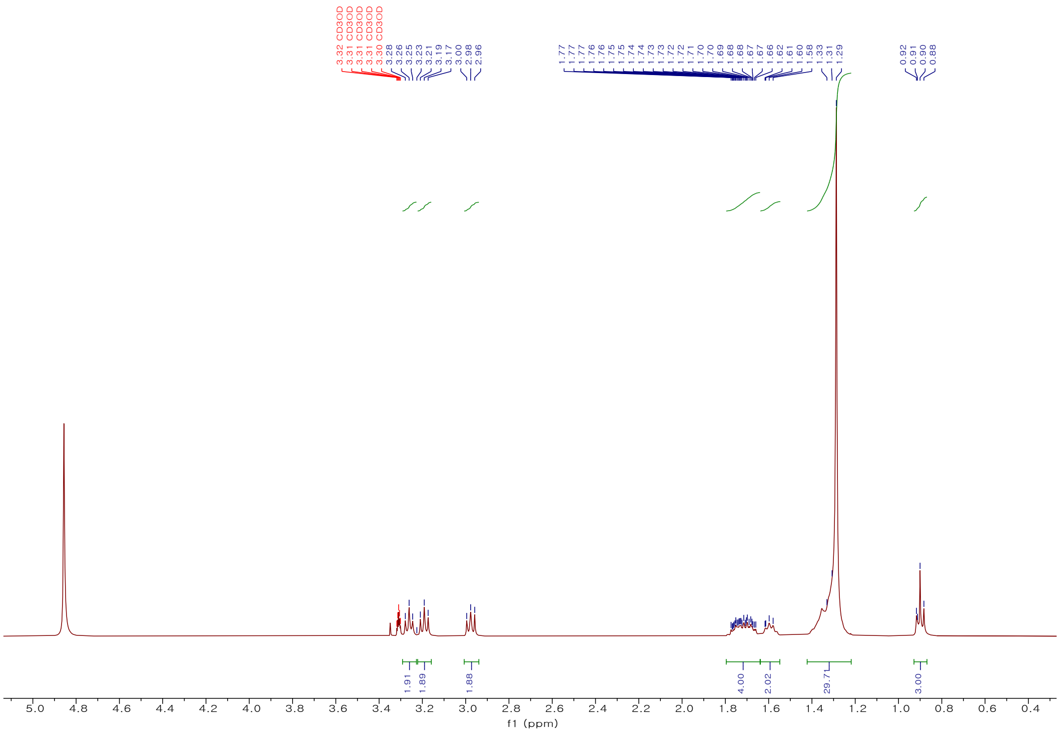

Figure S35. ^{1}H NMR spectrum of compound **5j** (400 MHz, CD_3OD)

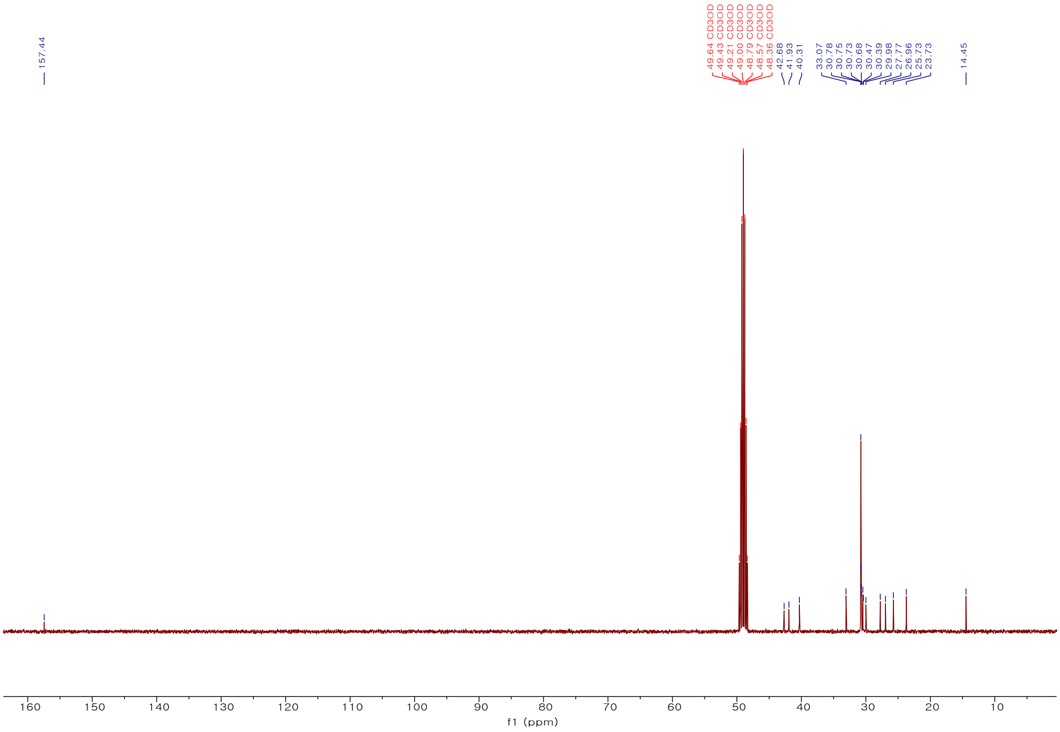

Figure S36. ^{13}C NMR spectrum of compound **5j** (101 MHz, CD_3OD)

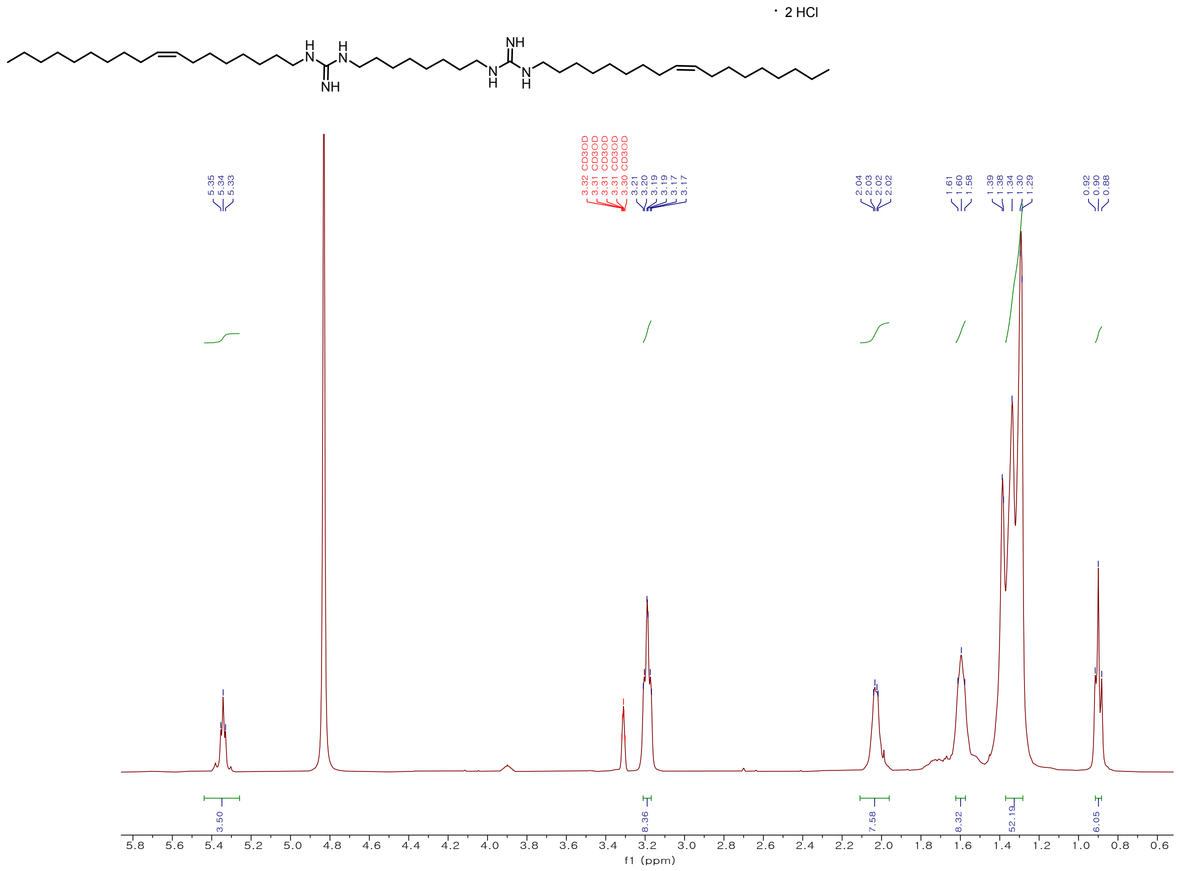

Figure S37. ^{1}H NMR spectrum of compound **6** (400 MHz, CD_3OD)

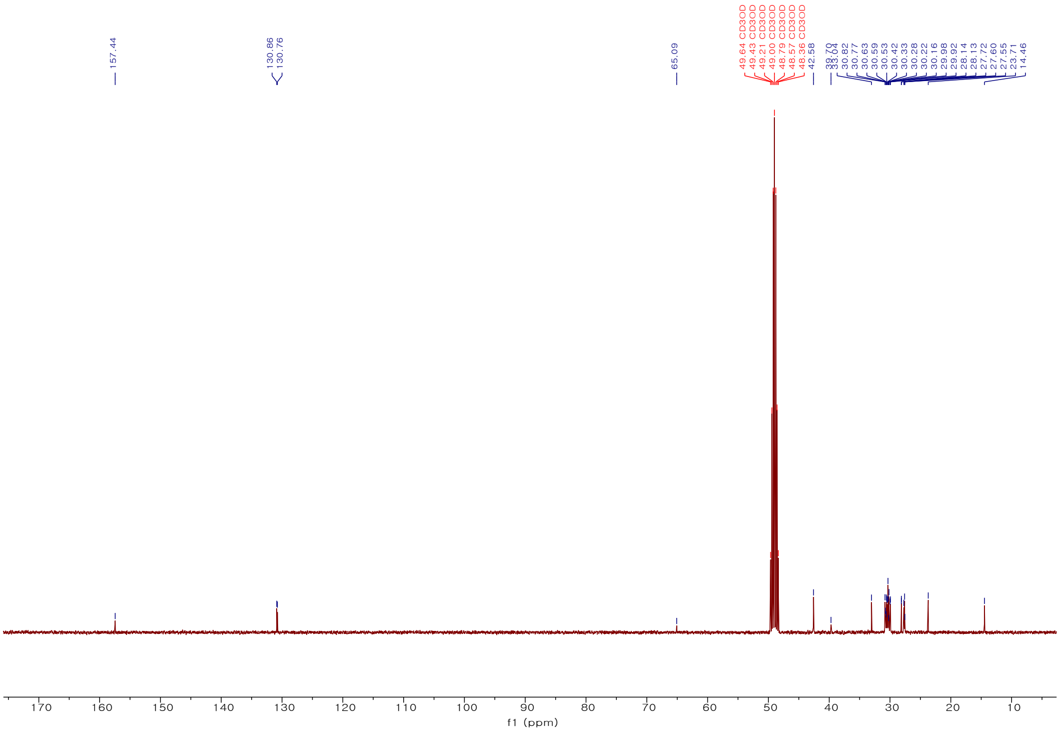

Figure S38. ^{13}C NMR spectrum of compound **6** (101 MHz, CD_3OD)

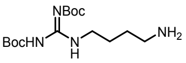

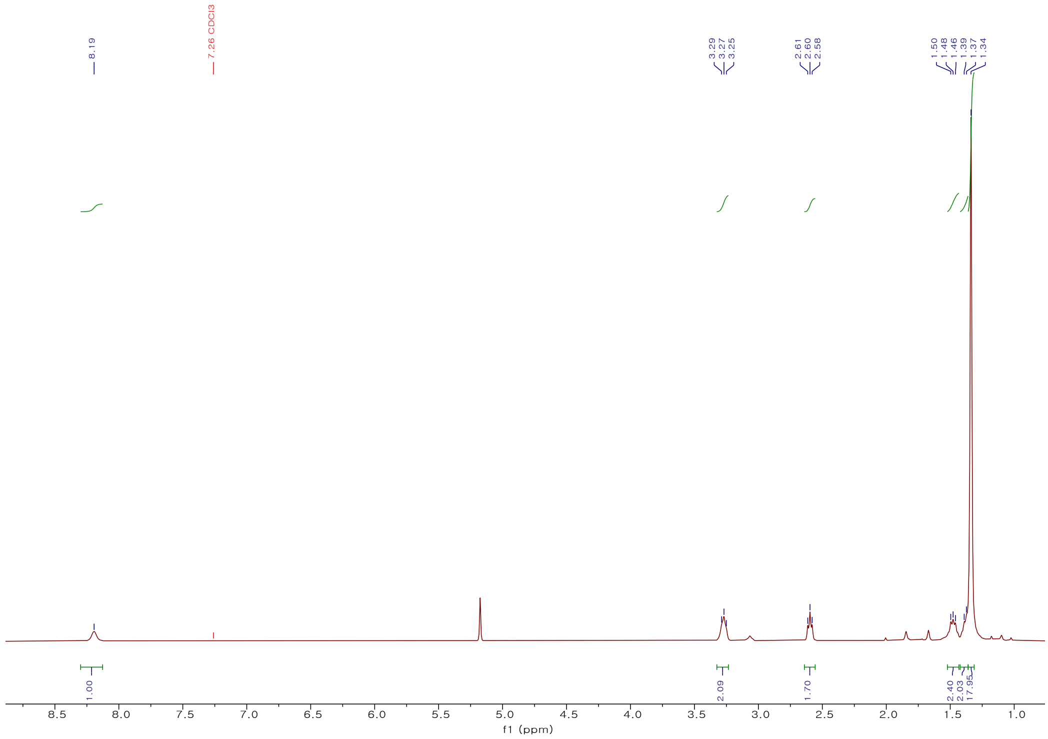

Figure S39. ^{1}H NMR spectrum of compound **7a** (400 MHz, $CDCl_3$)

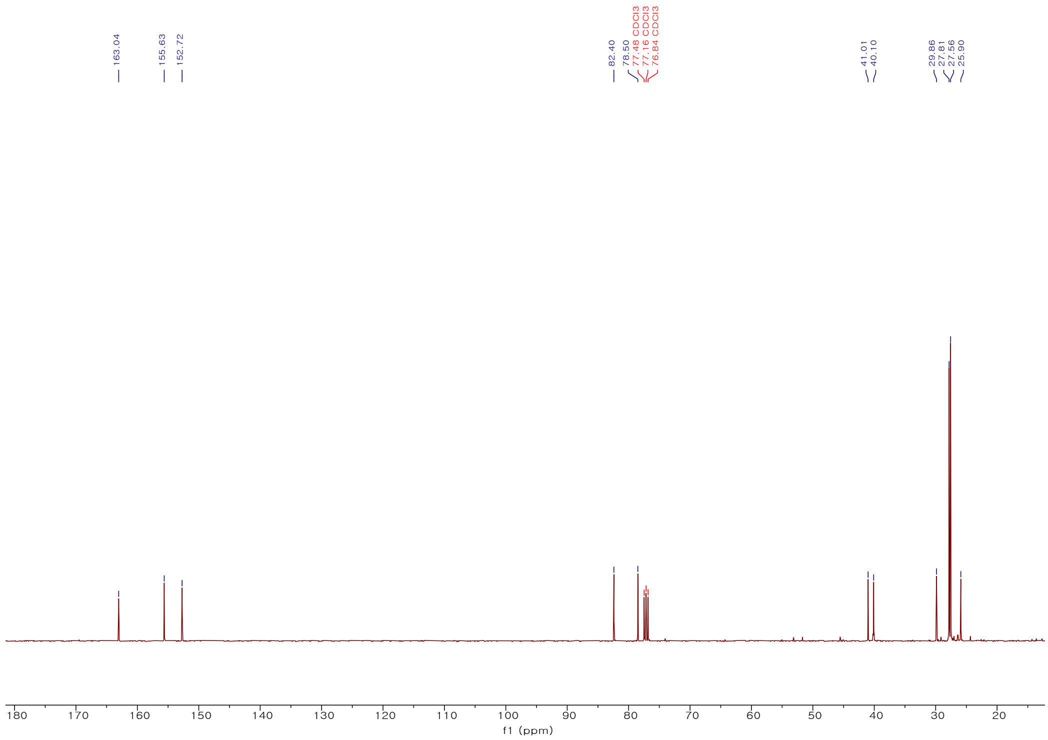

Figure S40. ^{13}C NMR spectrum of compound **7a** (101 MHz, $CDCl_3$)

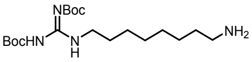

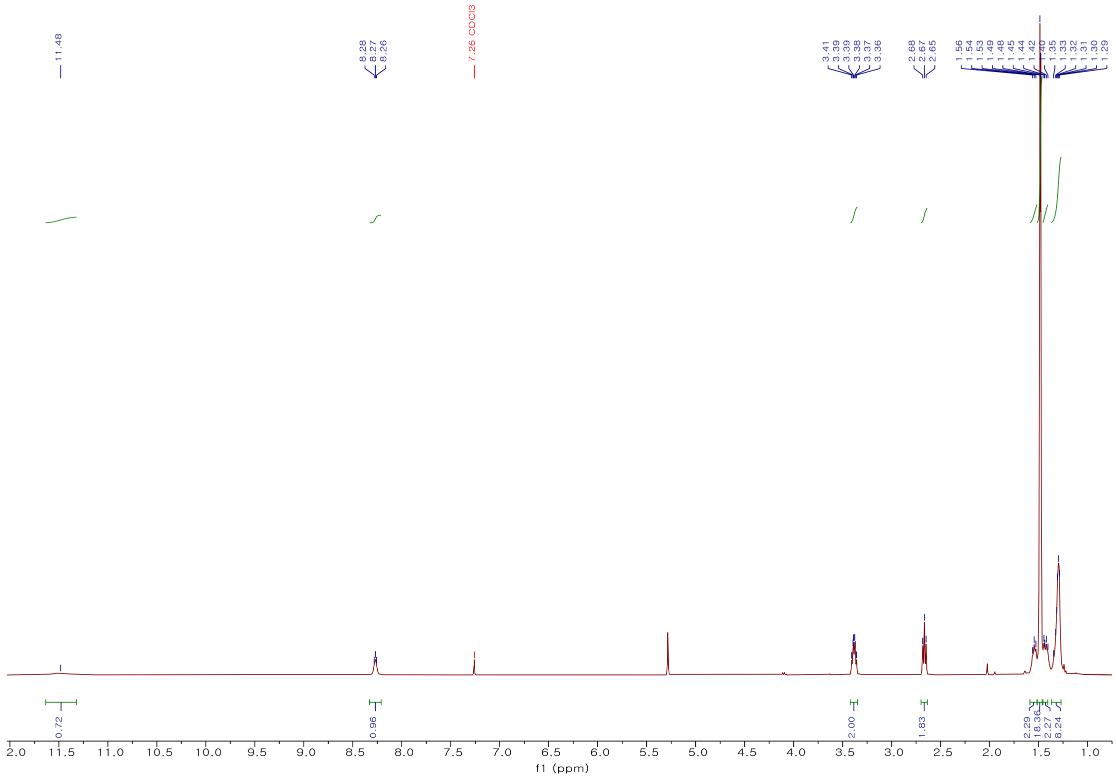

Figure S41. ^{1}H NMR spectrum of compound **7b** (400 MHz, $CDCl_3$)

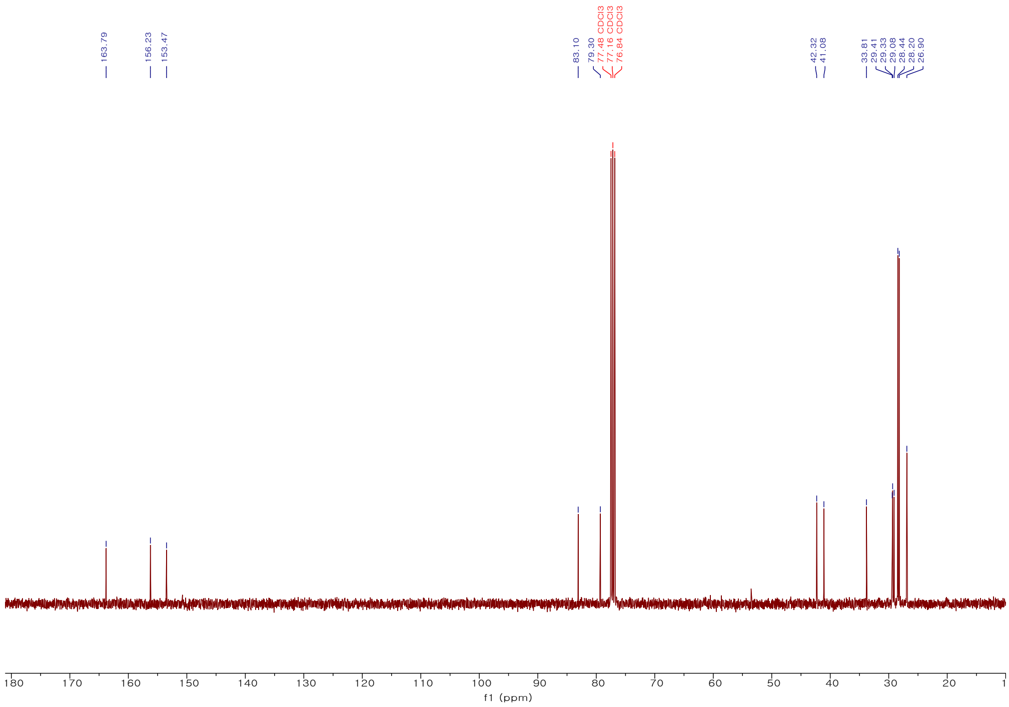

Figure S42. ^{13}C NMR spectrum of compound **7b** (101 MHz, $CDCl_3$)

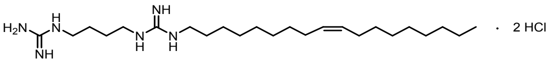

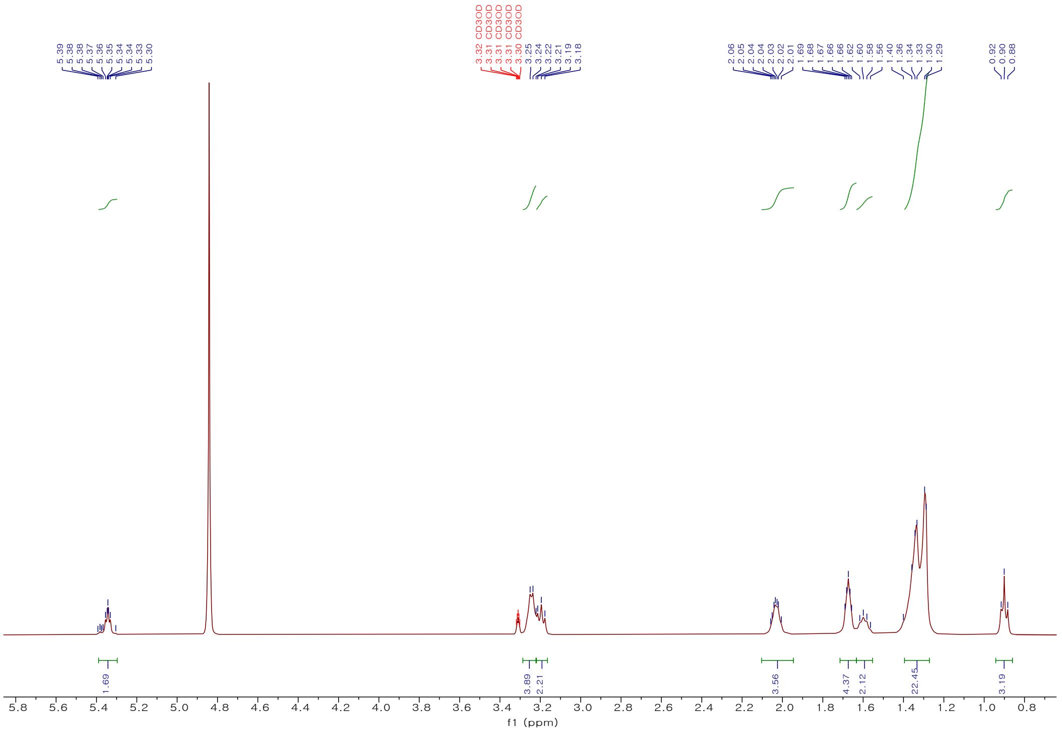

Figure S43. ^{1}H NMR spectrum of compound **8a** (400 MHz, CD_3OD)

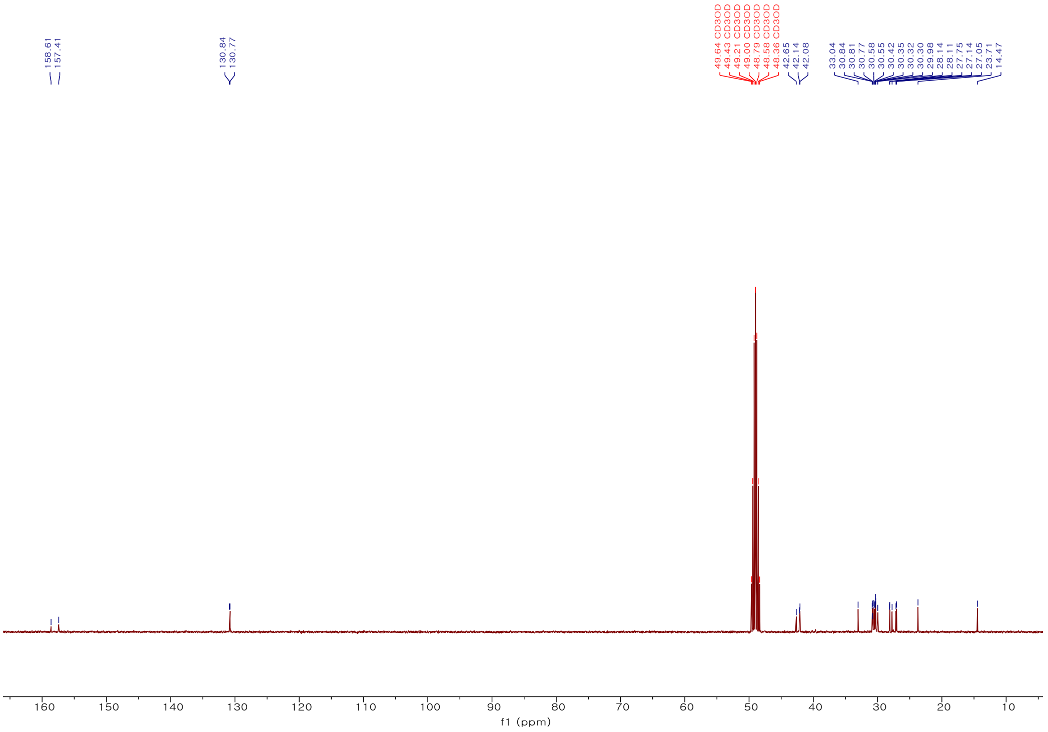

Figure S44. ^{13}C NMR spectrum of compound **8a** (101 MHz, CD_3OD)

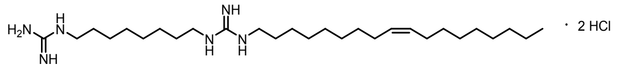

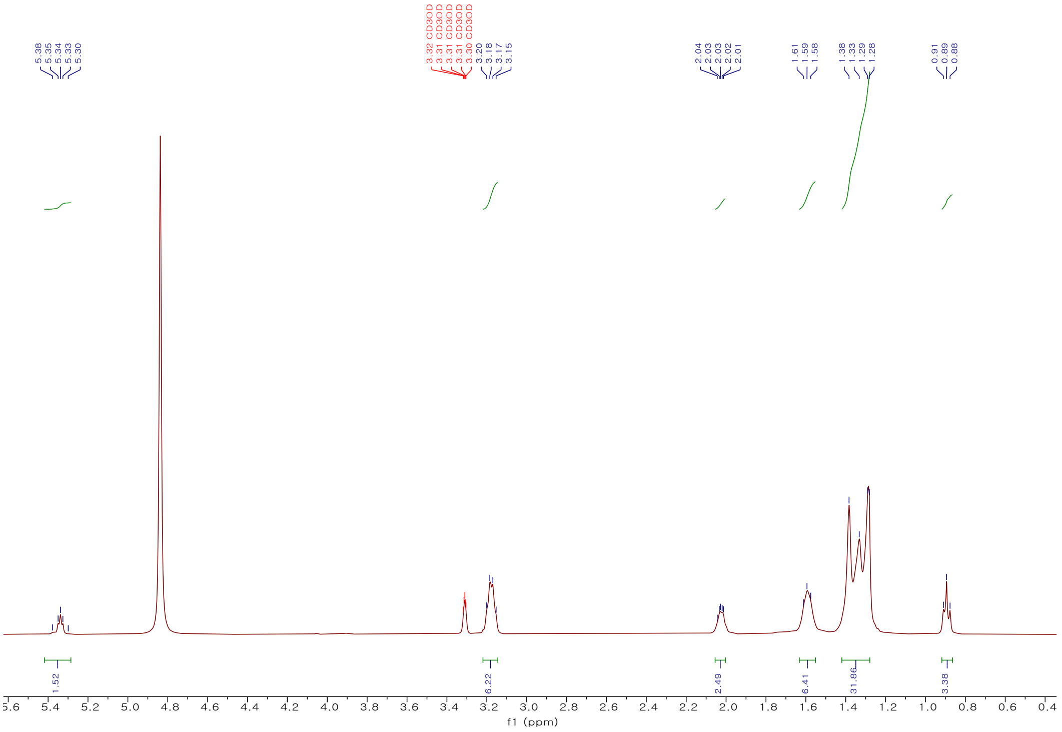

Figure S45. ^{1}H NMR spectrum of compound **8b** (400 MHz, CD_3OD)

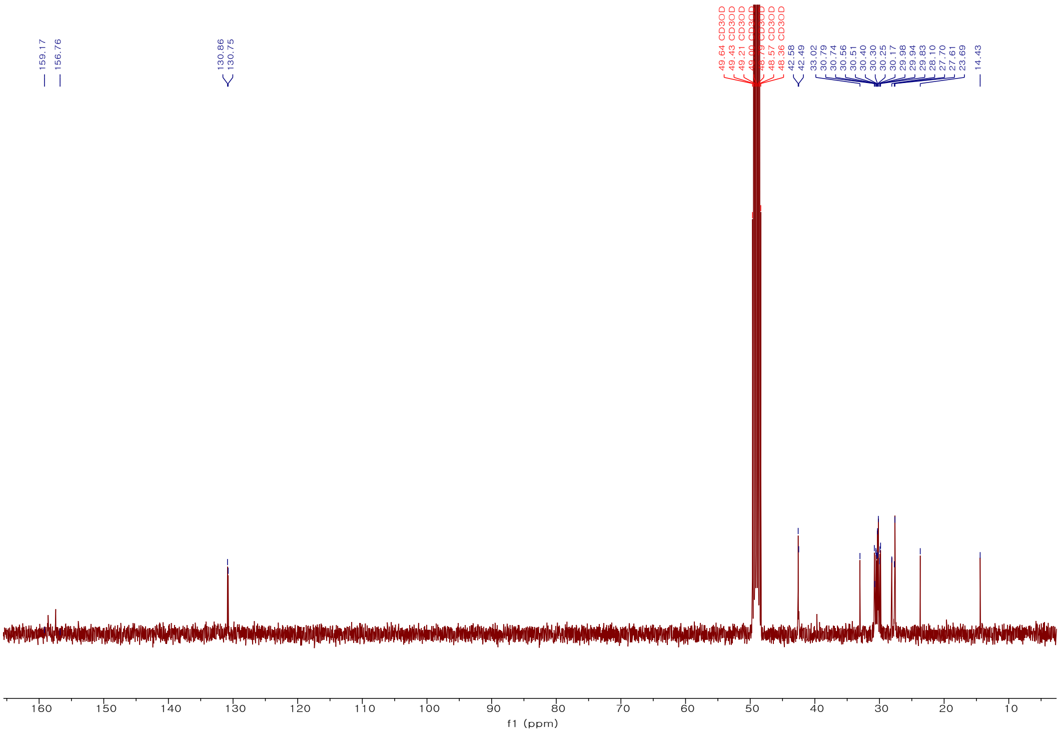

Figure S46. ^{13}C NMR spectrum of compound **8b** (101 MHz, CD_3OD)

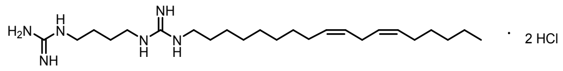

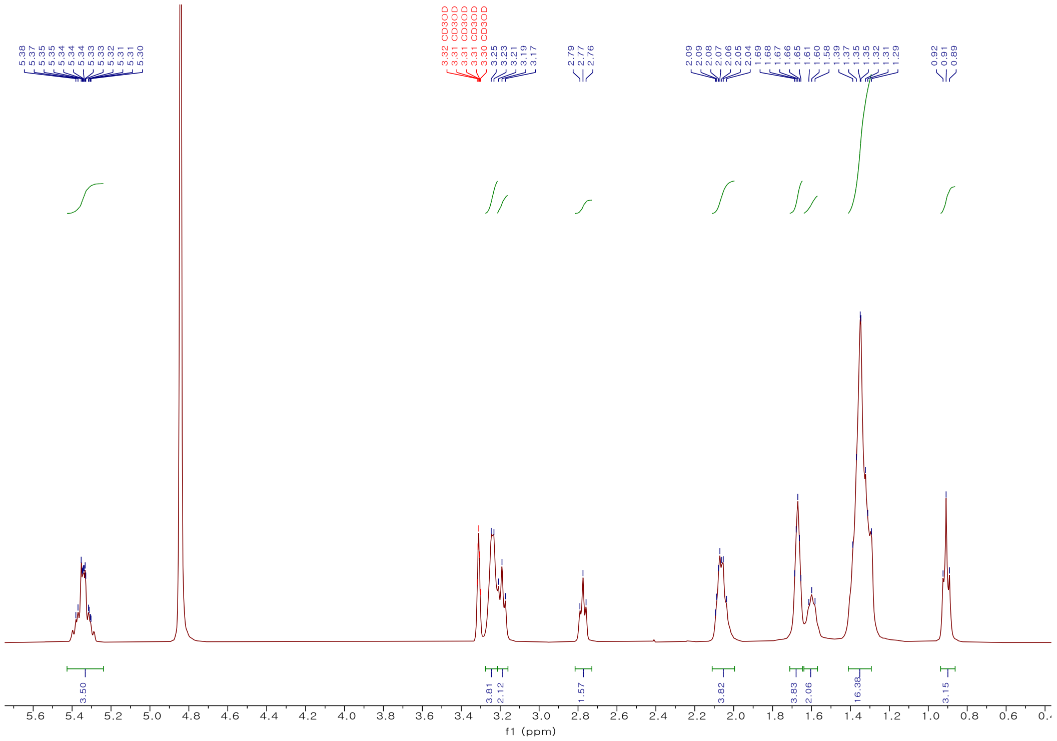

Figure S47. ^{1}H NMR spectrum of compound **8c** (400 MHz, CD_3OD)

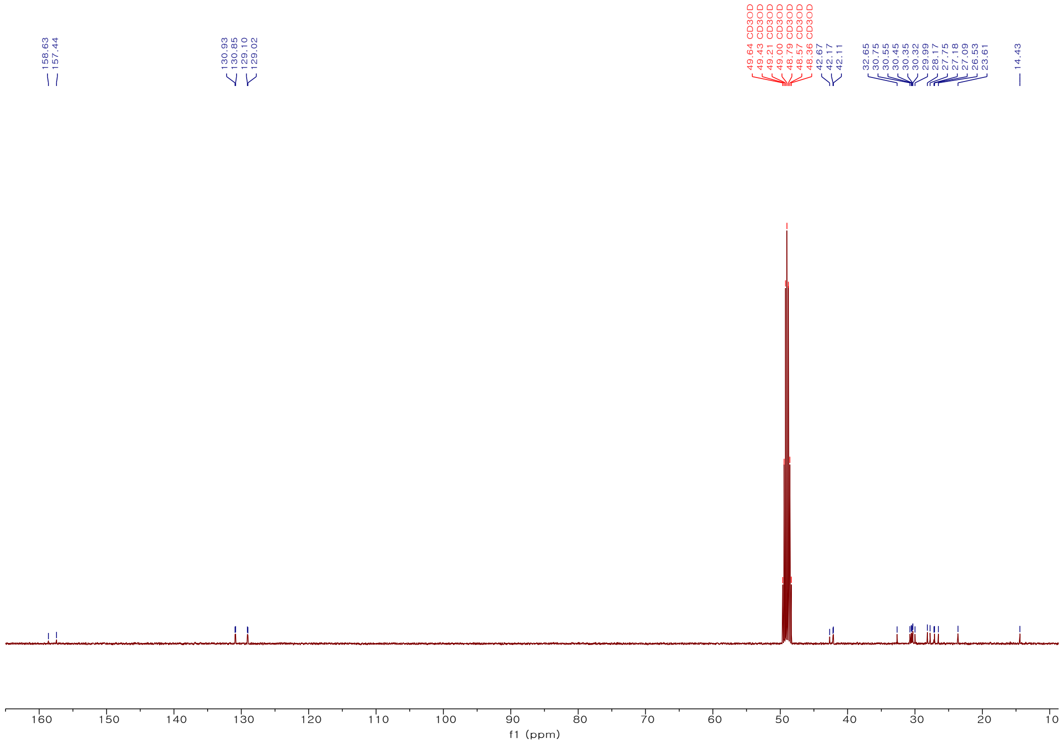

Figure S48. ^{13}C NMR spectrum of compound **8c** (101 MHz, CD_3OD)

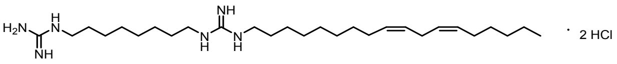

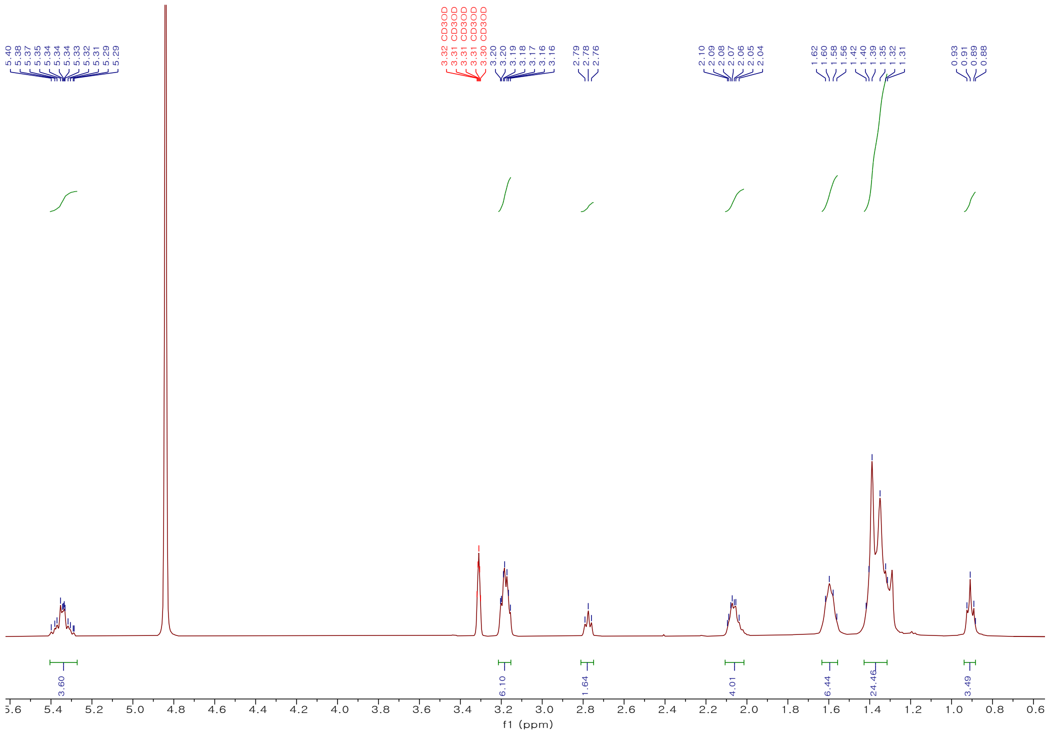

Figure S49. ^{1}H NMR spectrum of compound **8d** (400 MHz, CD_3OD)

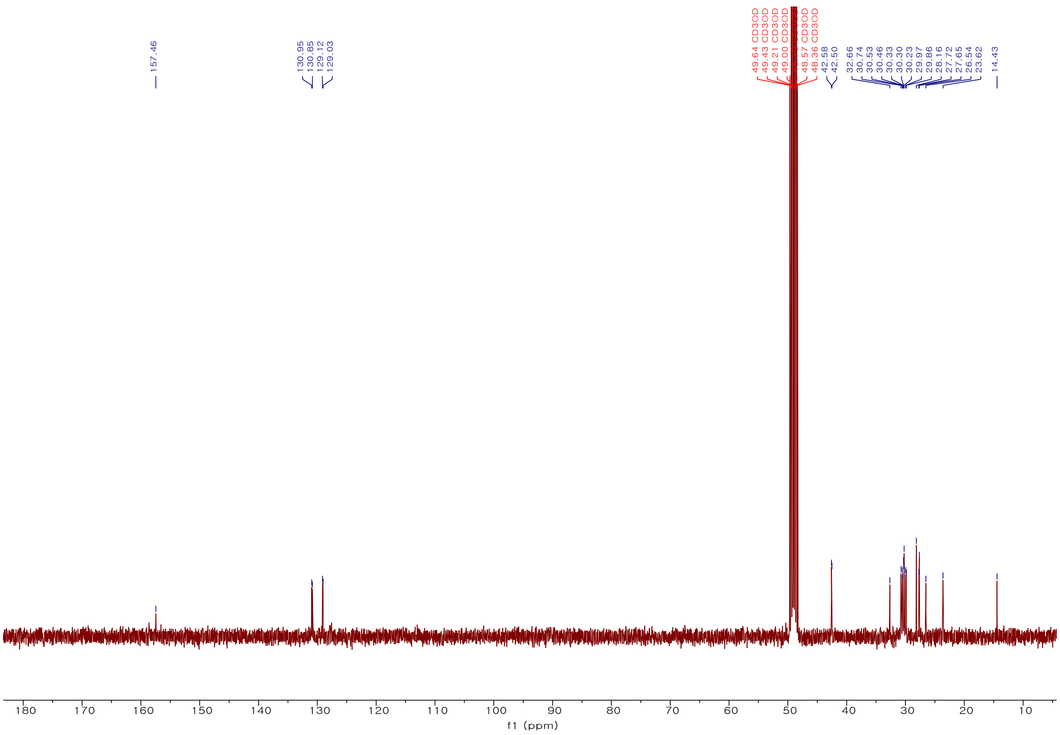

Figure S50. ^{13}C NMR spectrum of compound **8d** (101 MHz, CD_3OD)

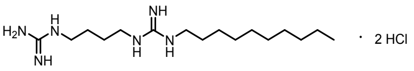

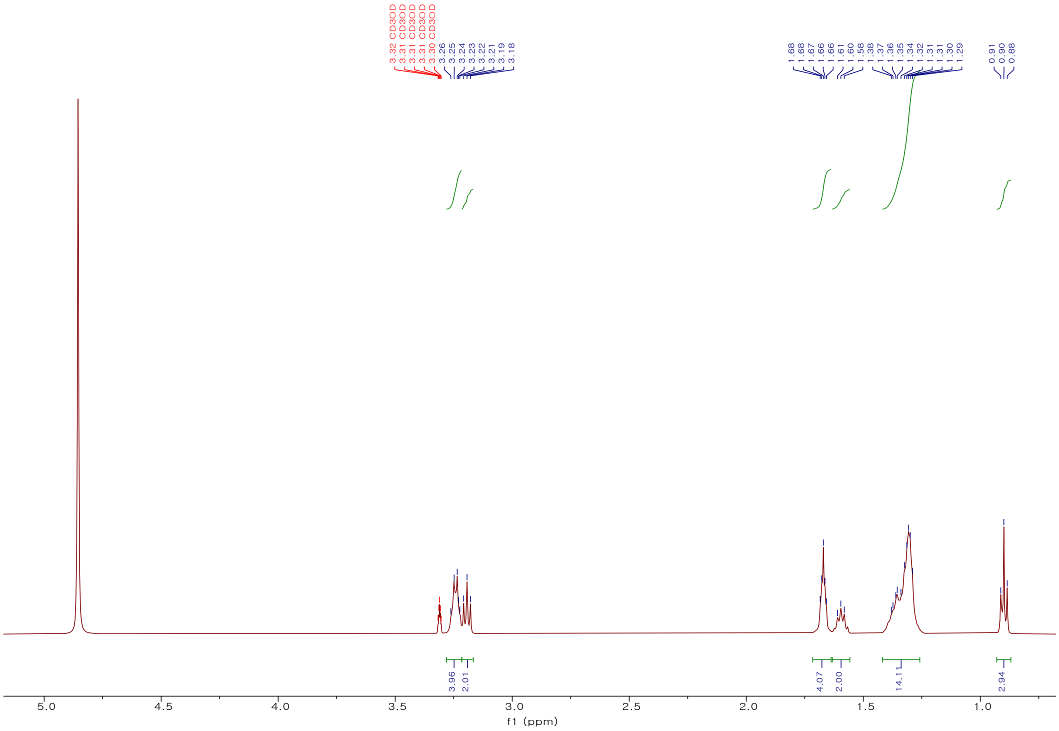

Figure S51. ^{1}H NMR spectrum of compound **8e** (400 MHz, CD_3OD)

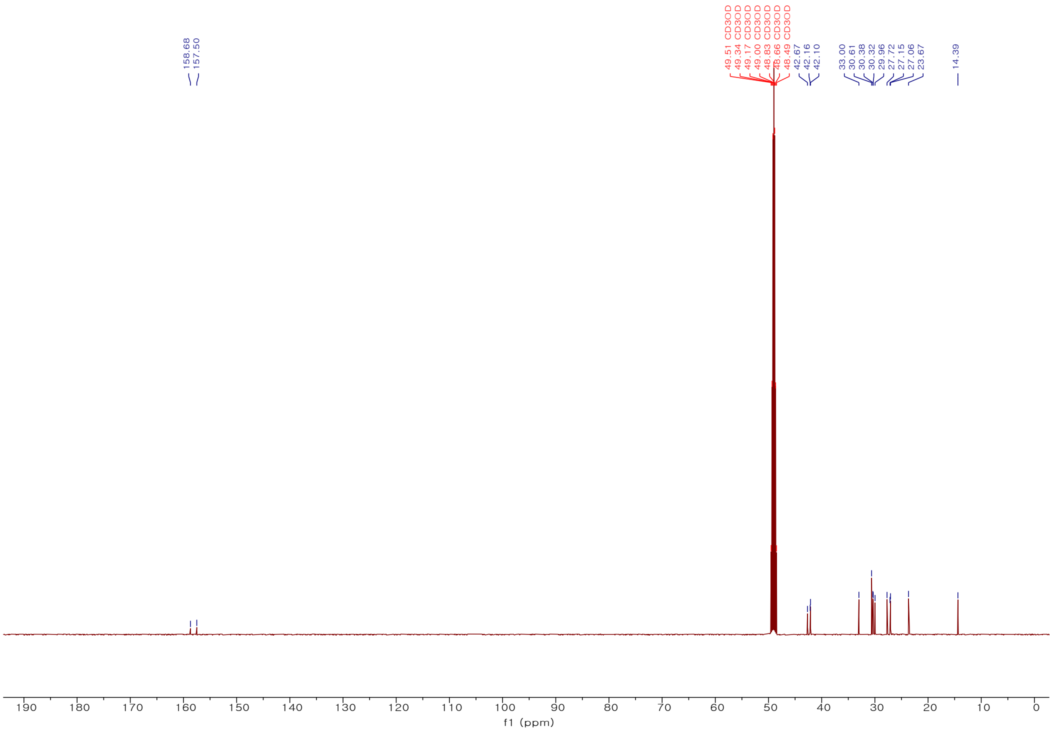

Figure S52. ^{13}C NMR spectrum of compound **8e** (101 MHz, CD_3OD)

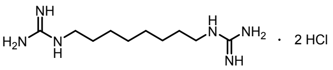

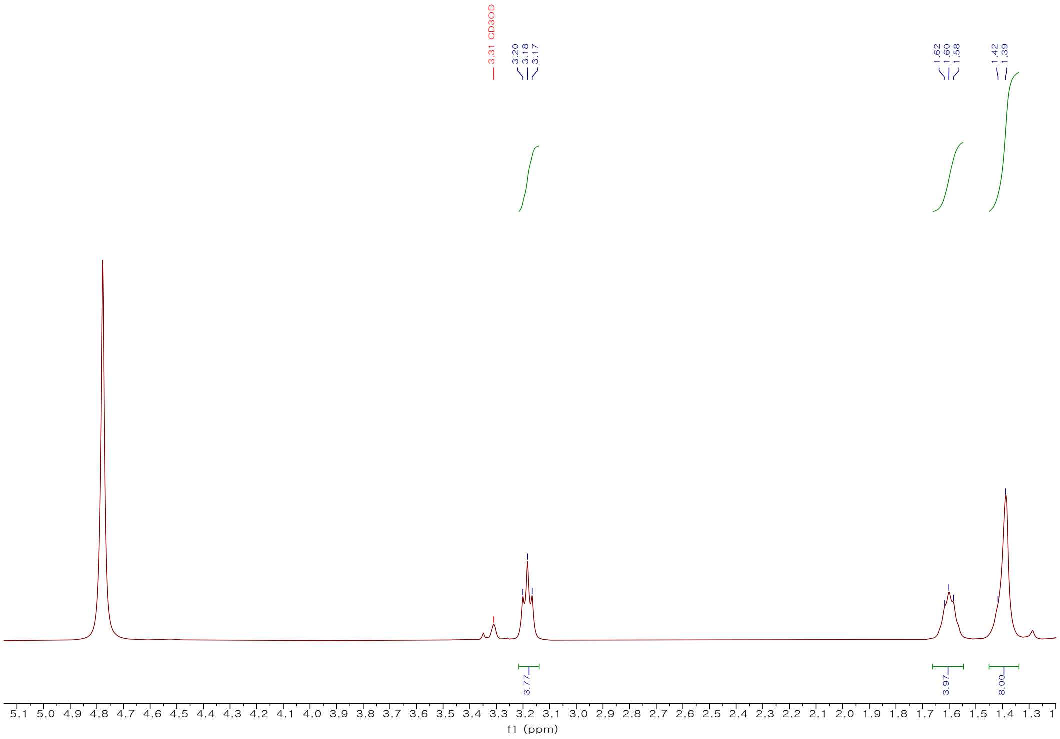

Figure S53. ^{1}H NMR spectrum of compound **8f** (400 MHz, CD_3OD)

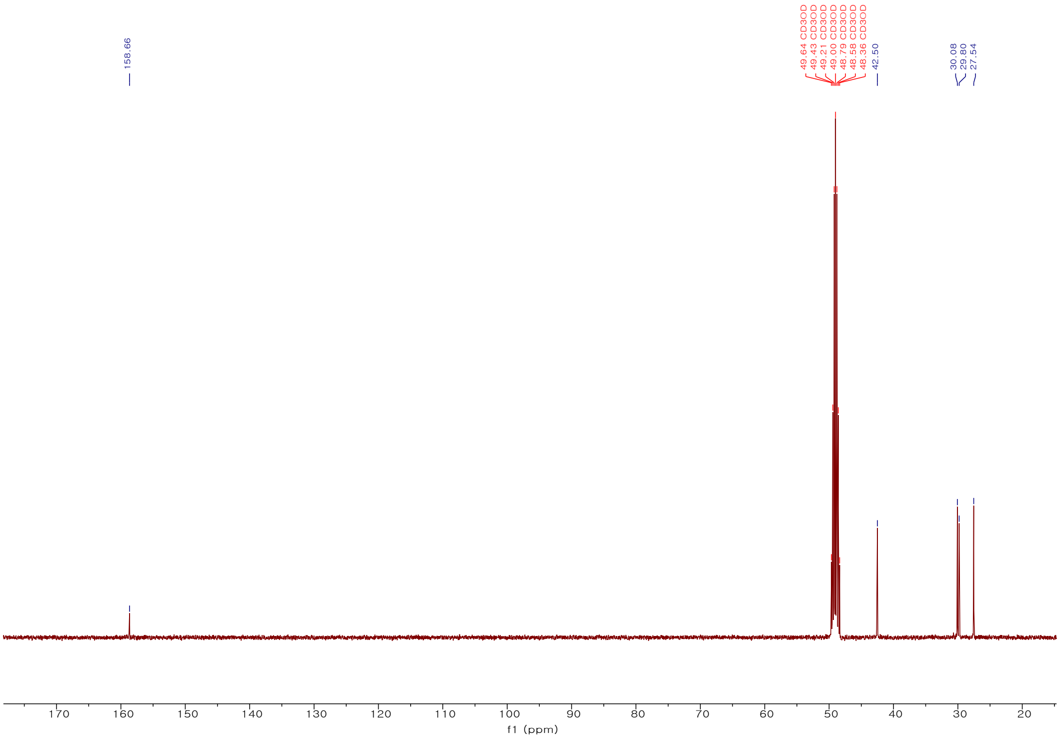

Figure S54. ^{13}C NMR spectrum of compound **8f** (101 MHz, CD_3OD)

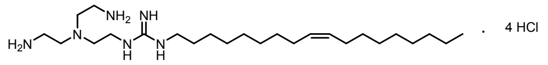

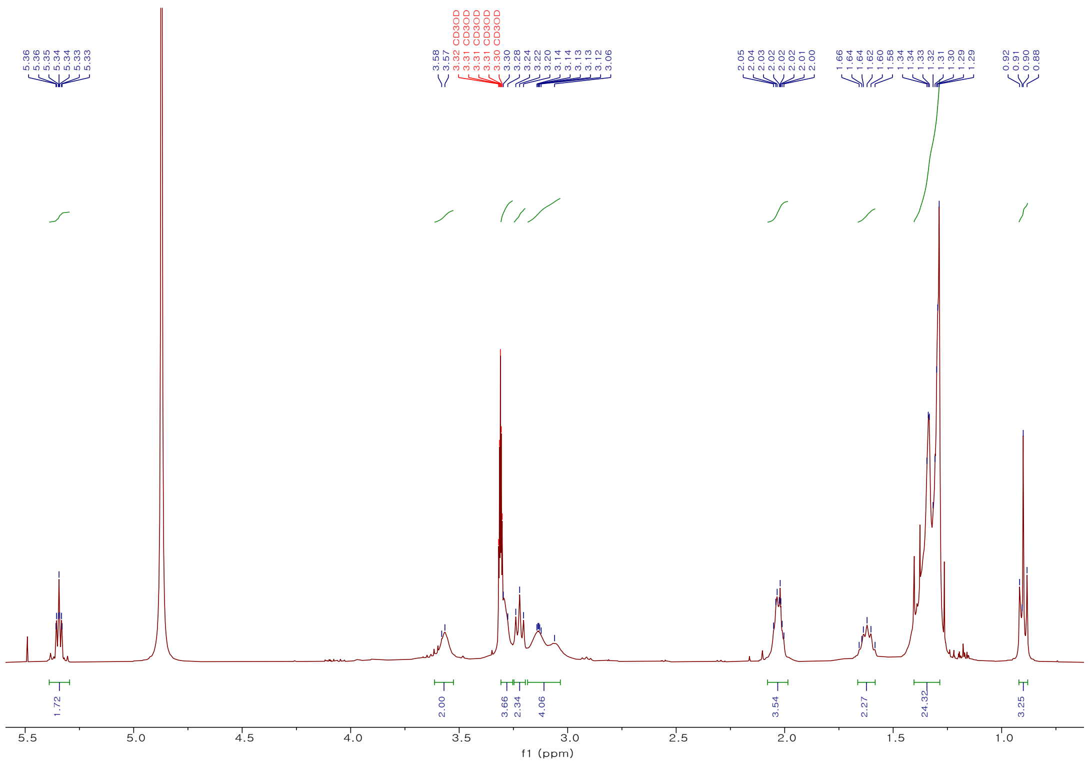

Figure S55. ^{1}H NMR spectrum of compound **10** (400 MHz, CD_3OD)

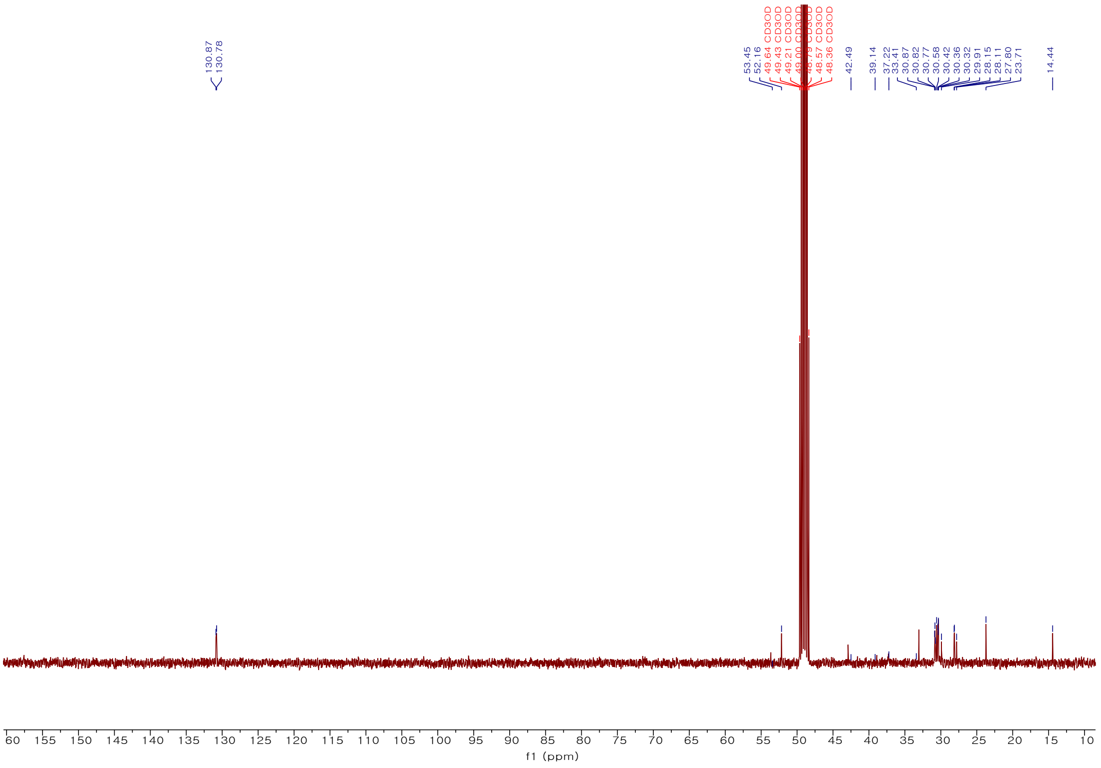

Figure S56. ^{13}C NMR spectrum of compound **10** (101 MHz, CD_3OD)

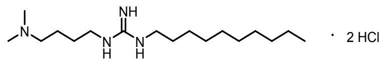

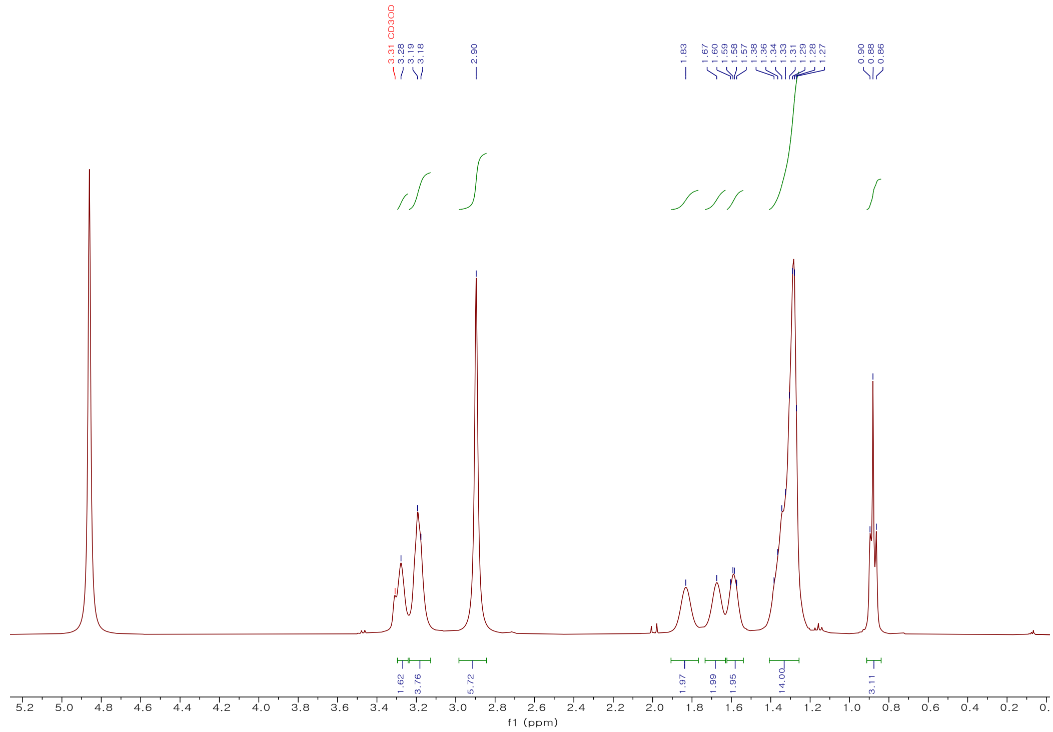

Figure S 57. ^{1}H NMR spectrum of compound **11a** (400 MHz, CD_3OD)

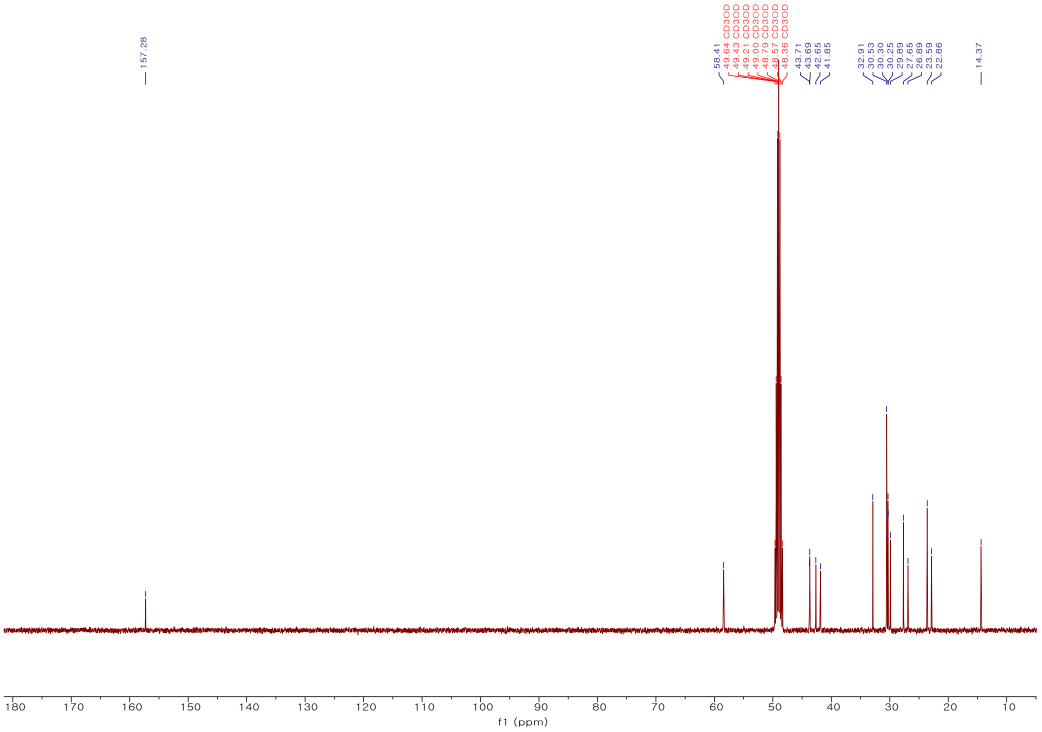

Figure S58. ^{13}C NMR spectrum of compound **11a** (101 MHz, CD_3OD)

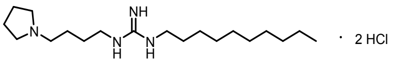

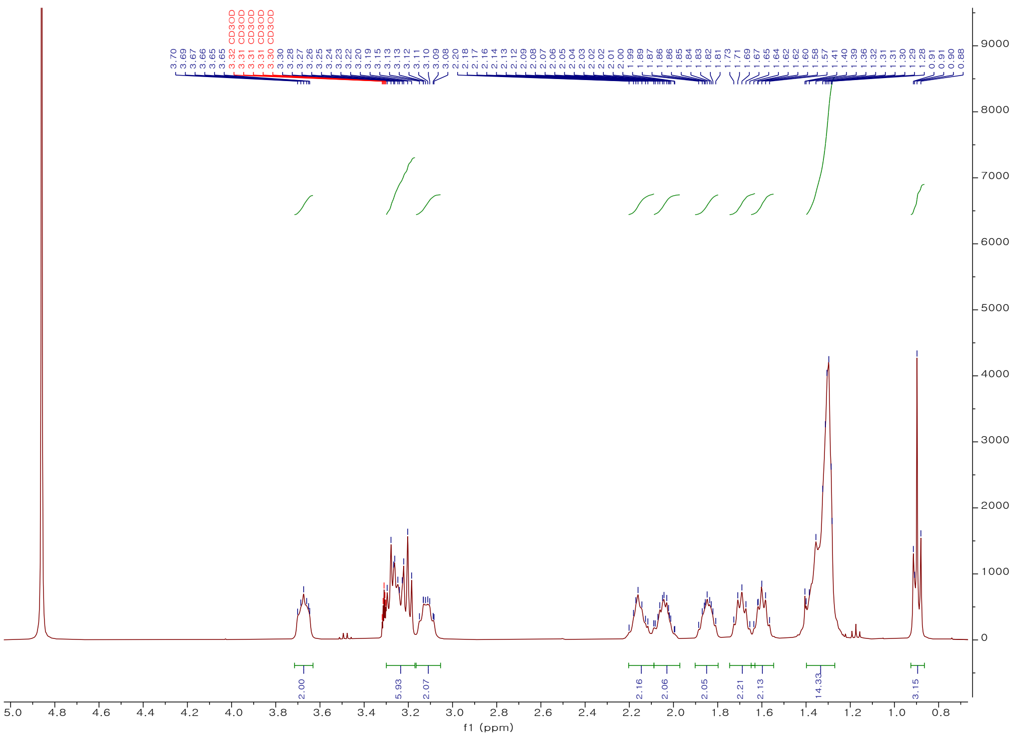

Figure S59. ^{1}H NMR spectrum of compound **11b** (400 MHz, CD_3OD)

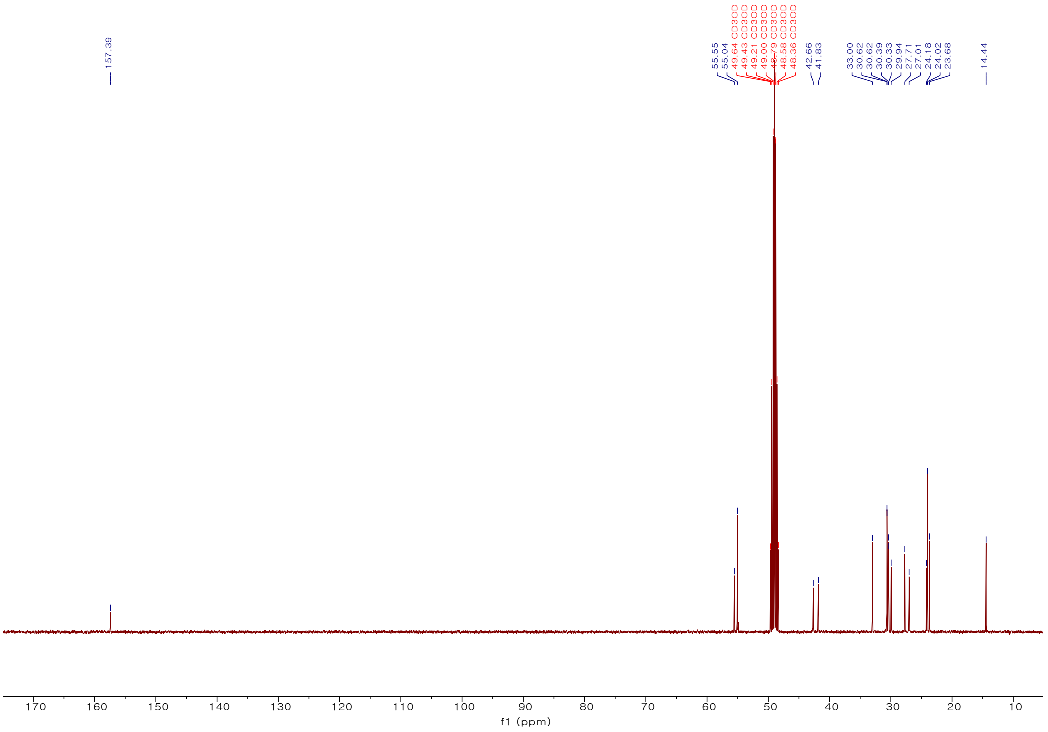

Figure S60. ^{13}C NMR spectrum of compound **11b** (101 MHz, CD_3OD)

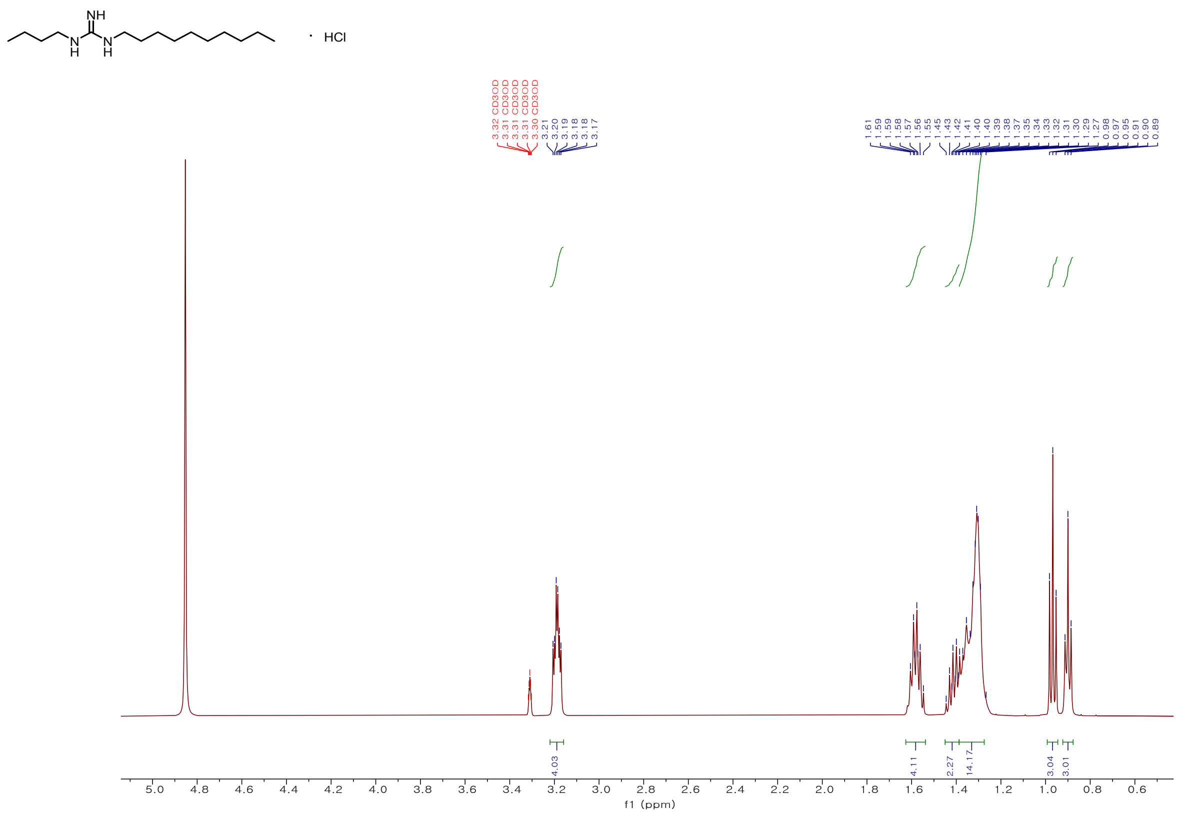

Figure S61. ^{1}H NMR spectrum of compound **11c** (400 MHz, CD_3OD)

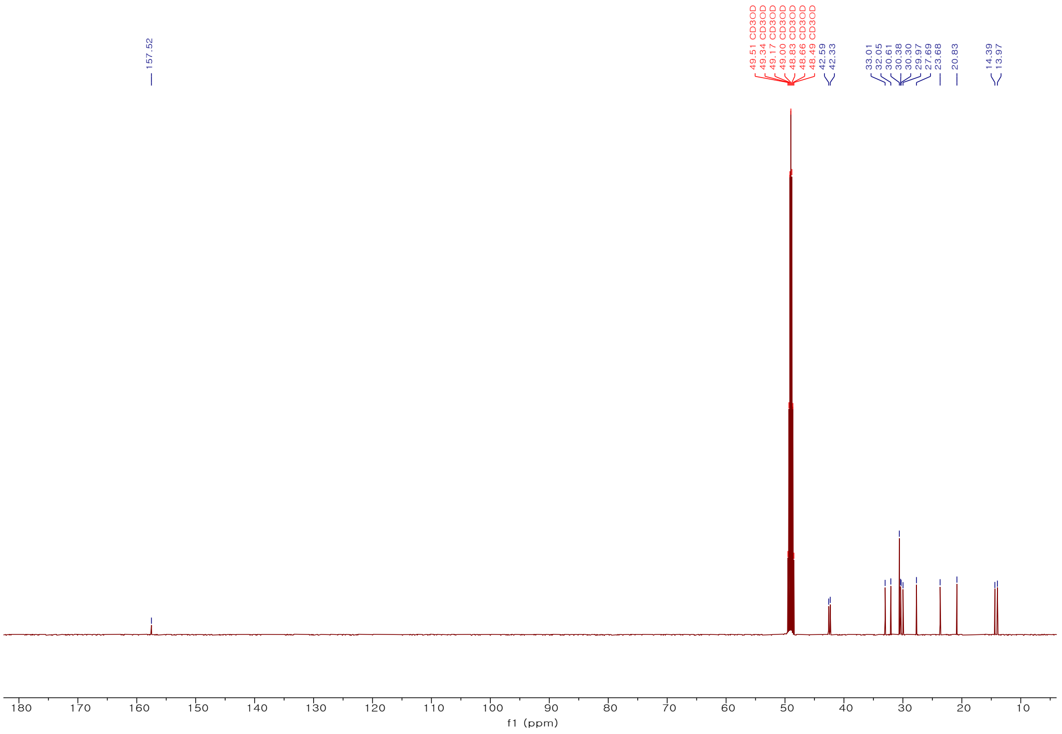

Figure S62. ^{13}C NMR spectrum of compound **11c** (101 MHz, CD_3OD)

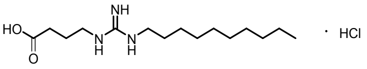

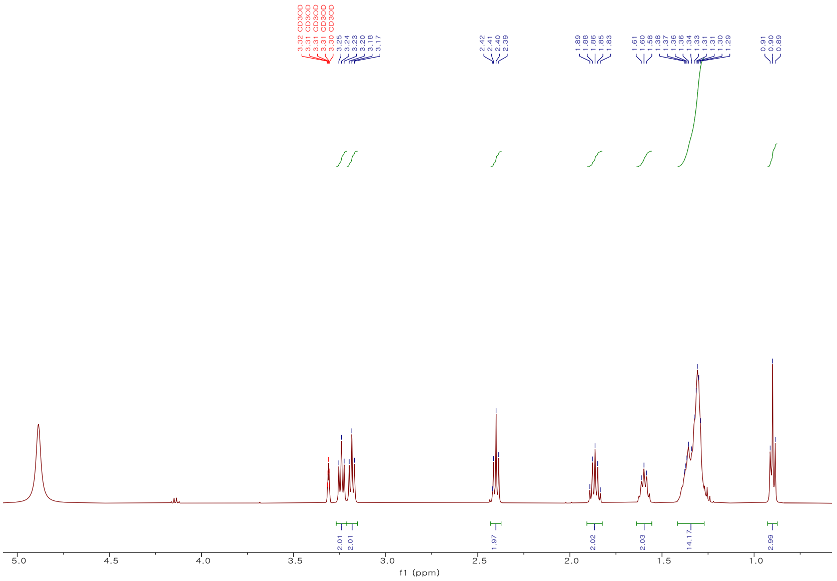

Figure S63. ^{1}H NMR spectrum of compound **11d** (400 MHz, CD_3OD)

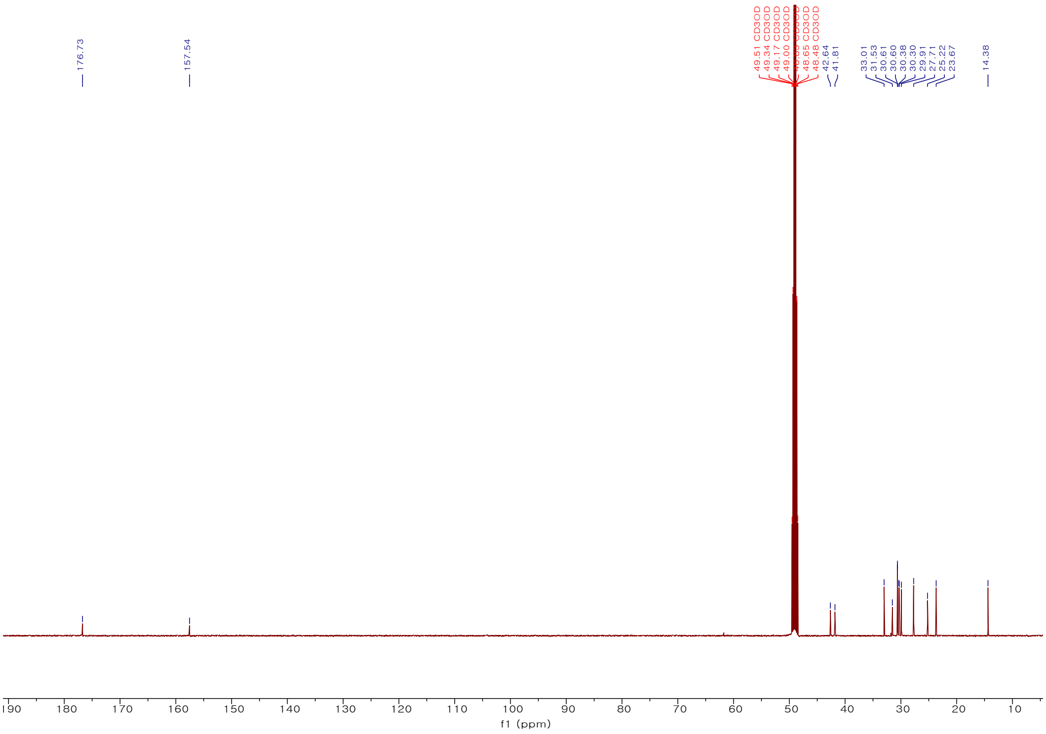

Figure S64. ^{13}C NMR spectrum of compound **11d** (101 MHz, CD_3OD)

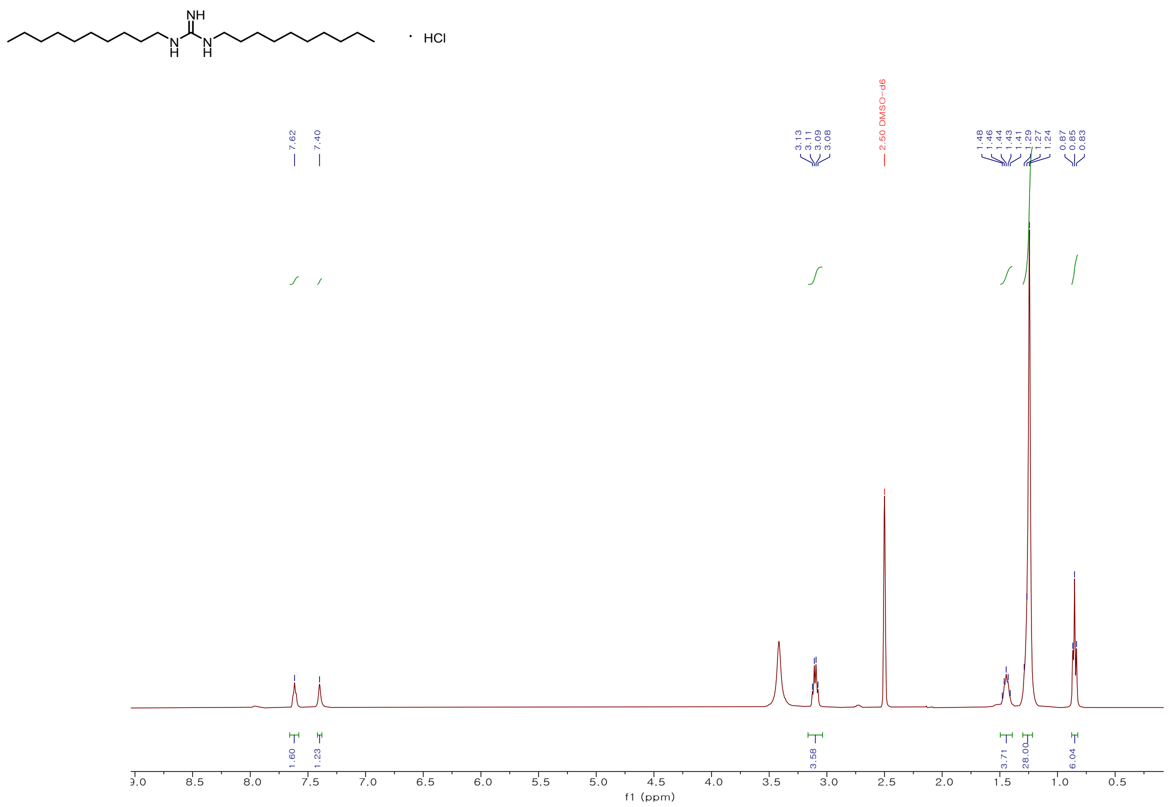

Figure S65. ^{1}H NMR spectrum of compound **11e** (400 MHz, DMSO-d_6)

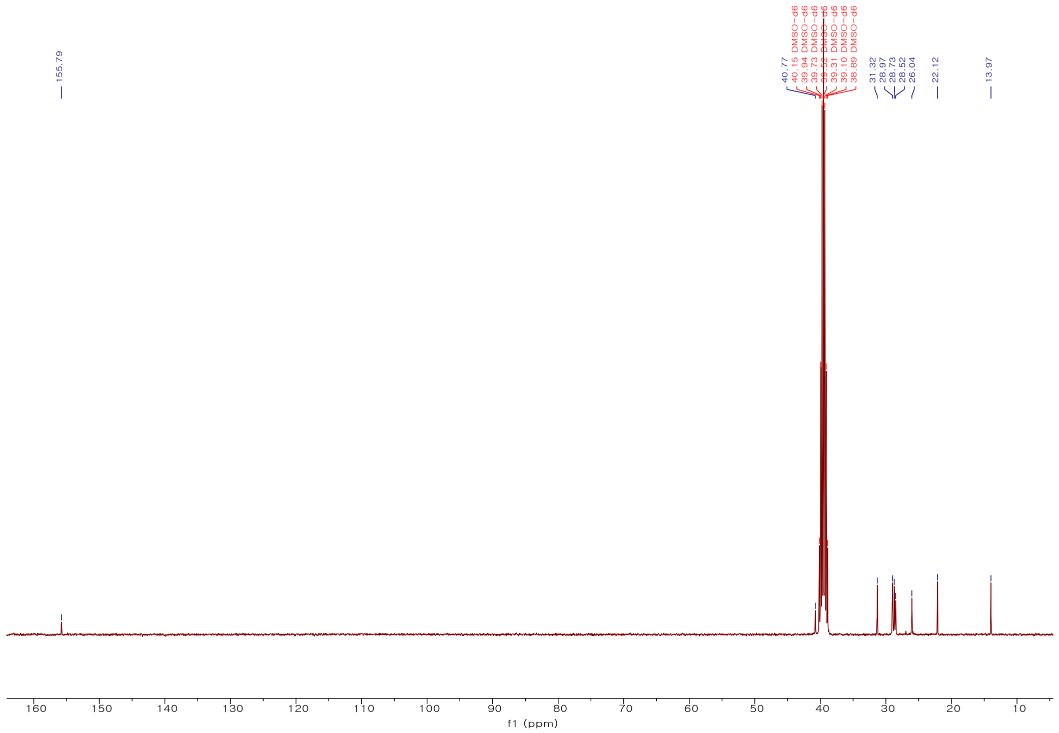

Figure S66. ^{13}C NMR spectrum of compound **11e** (101 MHz, DMSO-d_6)

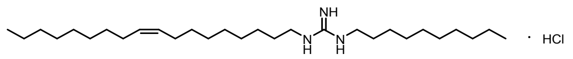

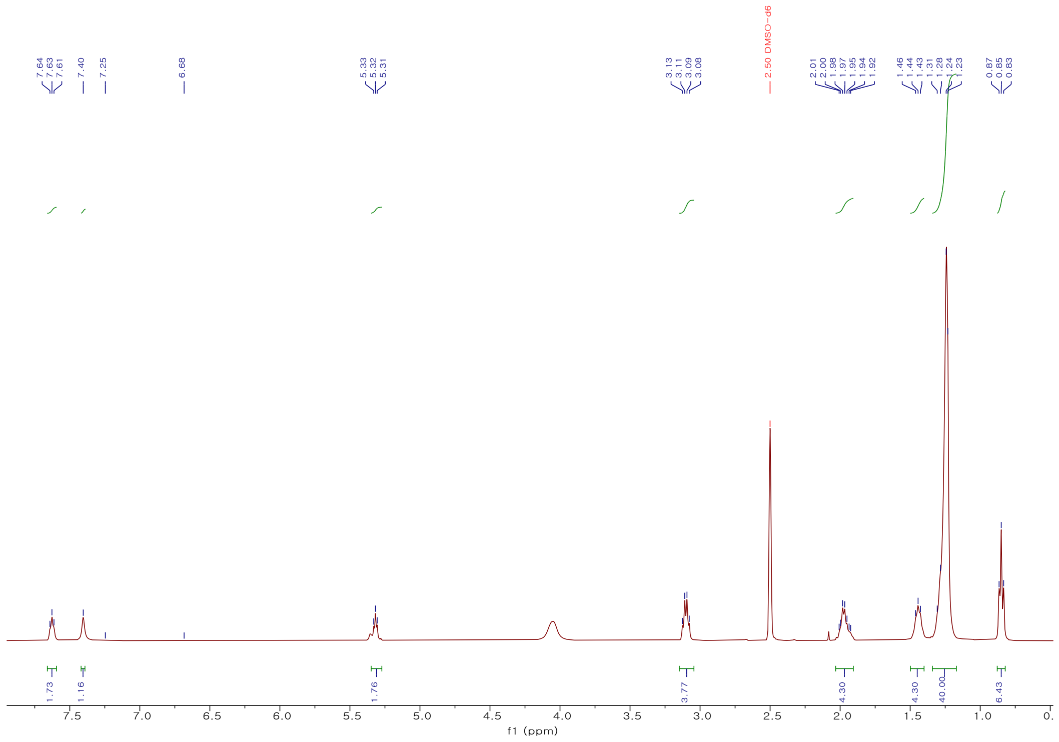

Figure S67. ^{1}H NMR spectrum of compound **11f** (400 MHz, DMSO-d_6)

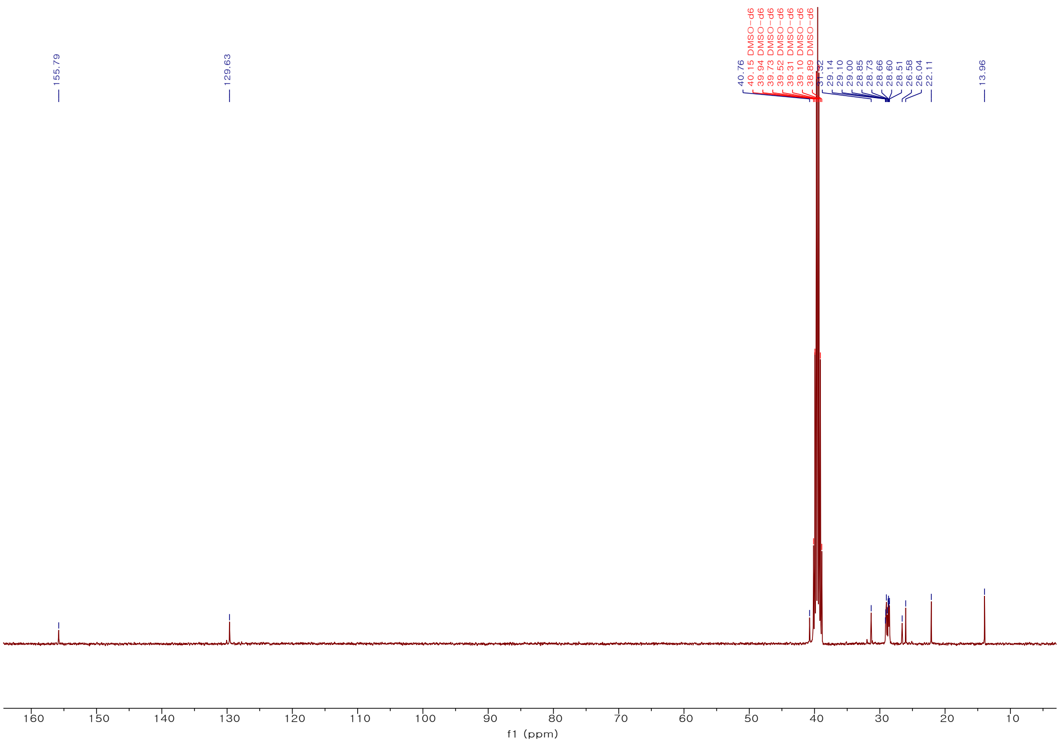

Figure S68. ^{13}C NMR spectrum of compound **11f** (101 MHz, DMSO-d_6)

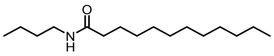

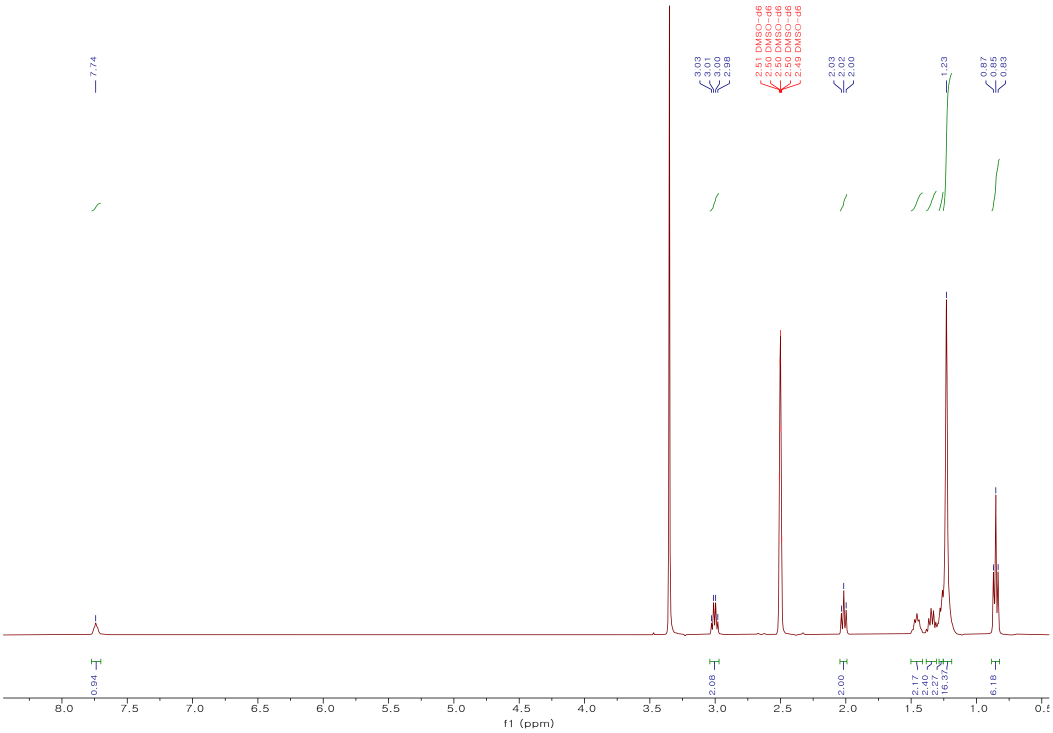

Figure S69. ^{1}H NMR spectrum of compound **13a** (400 MHz, DMSO-d_6)

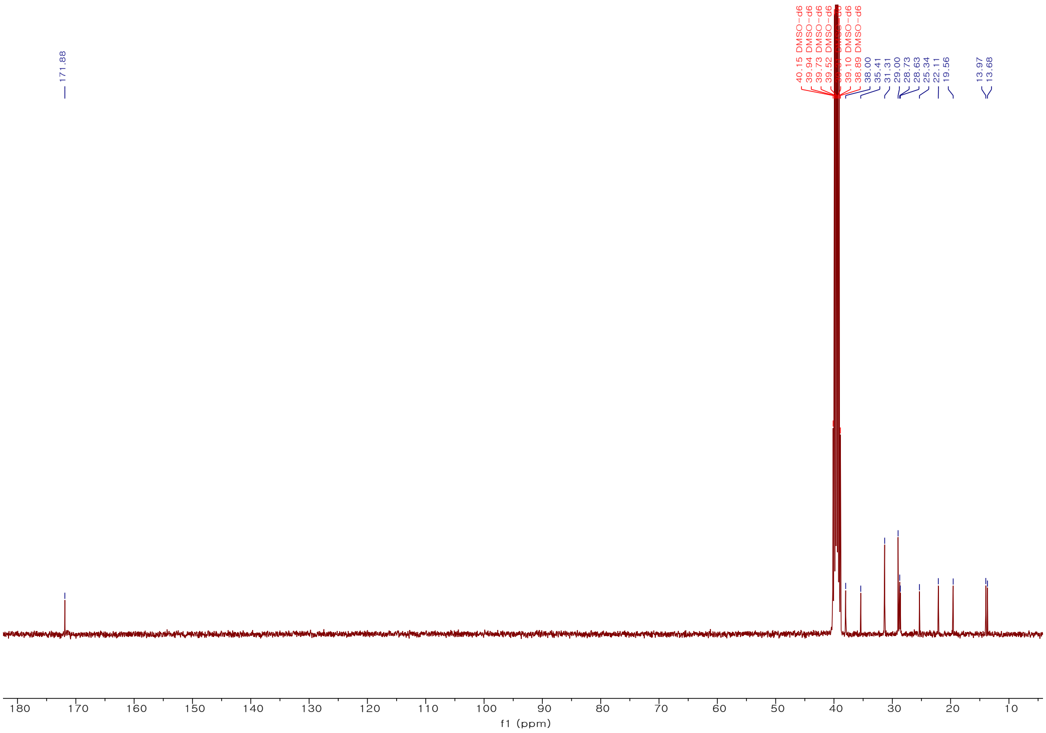

Figure S70. ^{13}C NMR spectrum of compound **13a** (101 MHz, DMSO-d_6)

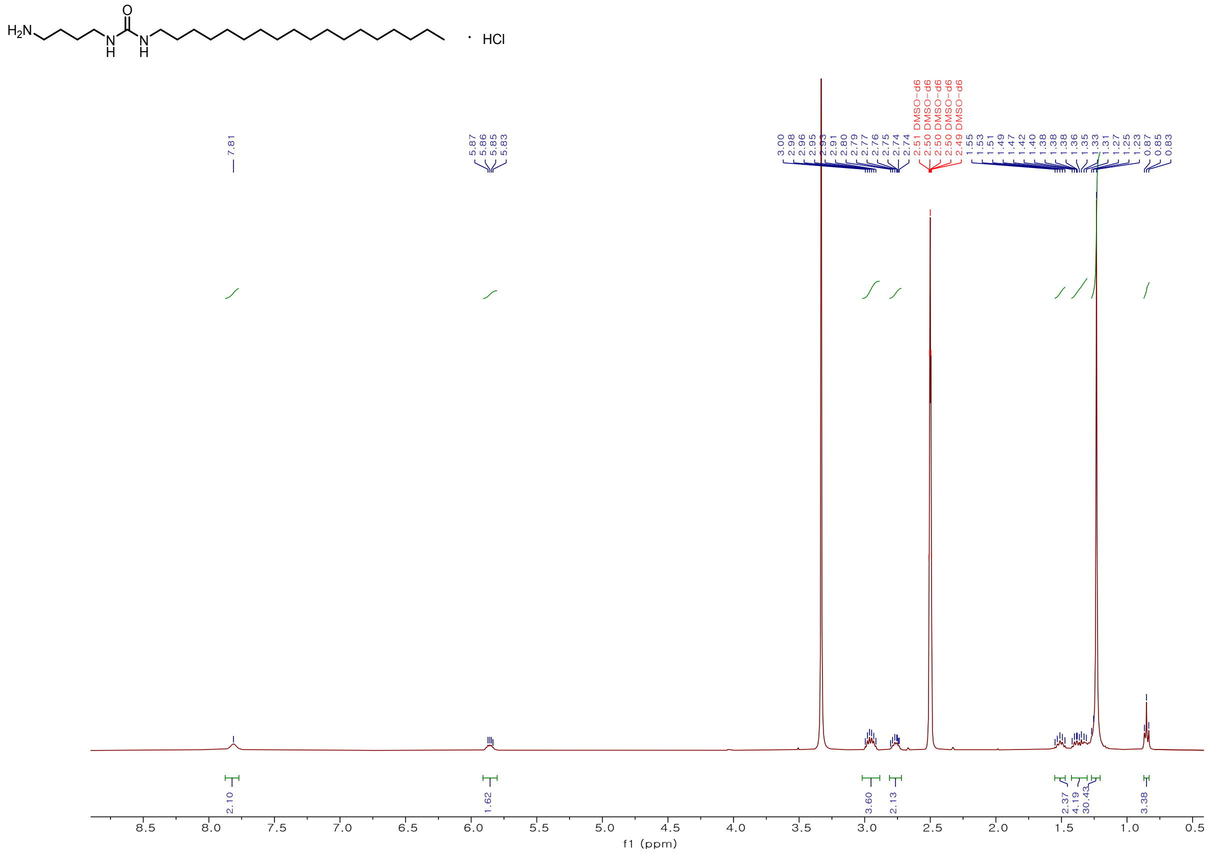

Figure S71. ^{1}H NMR spectrum of compound **15** (400 MHz, DMSO-d_6)

5. HPLC Chromatograms

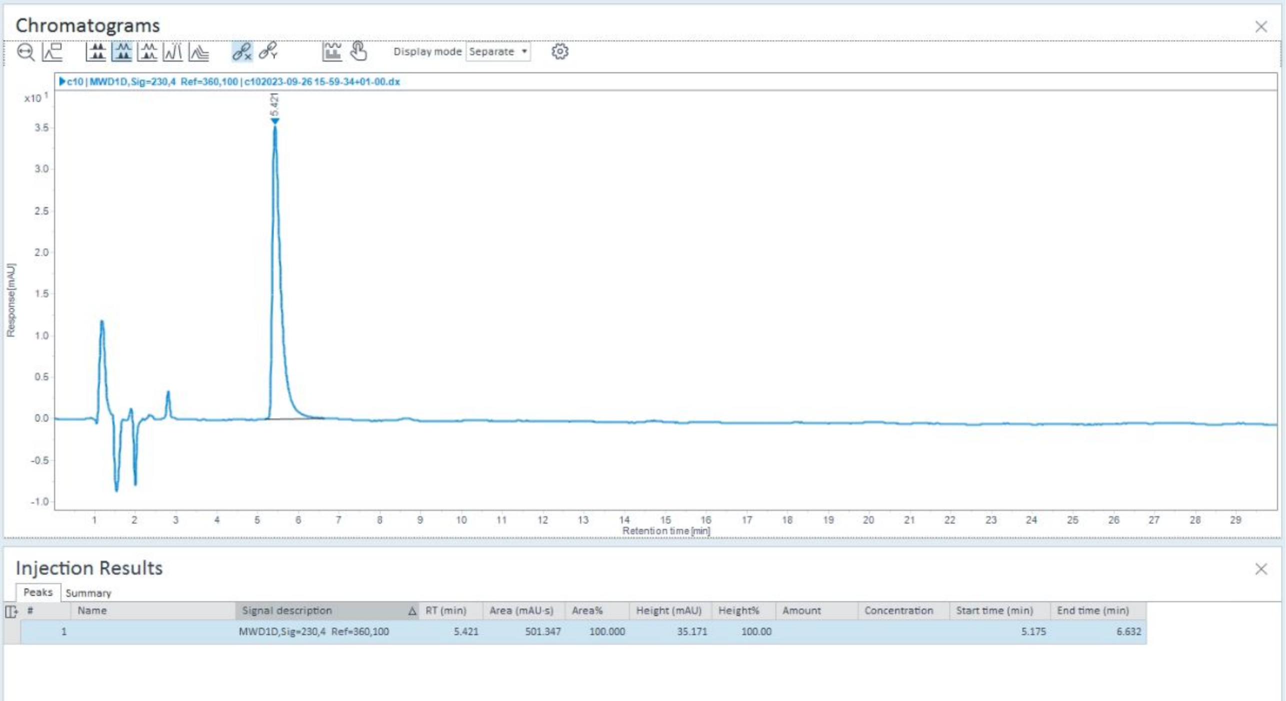

Figure S72. HPLC chromatogram of compound **5g**. Column: Agilent Eclipse Plus C18 column Eluent: H_2O (0.1% DEA was added in H_2O):ACN 50:50. Flow rate: 1 mL/min. Detection wavelength: 230 nm.
